# The four hexamerin genes in the honey bee: structure, molecular evolution and function deduced from expression patterns in queens, workers and drones

**DOI:** 10.1186/1471-2199-11-23

**Published:** 2010-03-26

**Authors:** Juliana R Martins, Francis MF Nunes, Alexandre S Cristino, Zilá LP Simões, Márcia MG Bitondi

**Affiliations:** 1 Faculdade de Medicina de Ribeirão Preto, Departamento de Genética, Universidade de São Paulo, Ribeirão Preto, SP, Brazil; 2 Faculdade de Filosofia, Ciências e Letras de Ribeirão Preto, Departamento de Biologia, Universidade de São Paulo, Ribeirão Preto, SP, Brazil; 3 The Queensland Brain Institute, The University of Queensland, Brisbane, Queensland, Australia

## Abstract

**Background:**

Hexamerins are hemocyanin-derived proteins that have lost the ability to bind copper ions and transport oxygen; instead, they became storage proteins. The current study aimed to broaden our knowledge on the hexamerin genes found in the honey bee genome by exploring their structural characteristics, expression profiles, evolution, and functions in the life cycle of workers, drones and queens.

**Results:**

The hexamerin genes of the honey bee (*hex 70a*, *hex 70b*, *hex 70c *and *hex 110*) diverge considerably in structure, so that the overall amino acid identity shared among their deduced protein subunits varies from 30 to 42%. Bioinformatics search for motifs in the respective upstream control regions (UCRs) revealed six overrepresented motifs including a potential binding site for Ultraspiracle (Usp), a target of juvenile hormone (JH). The expression of these genes was induced by topical application of JH on worker larvae. The four genes are highly transcribed by the larval fat body, although with significant differences in transcript levels, but only *hex 110 *and *hex 70a *are re-induced in the adult fat body in a caste- and sex-specific fashion, workers showing the highest expression. Transcripts for *hex 110*, *hex 70a *and *hex70b *were detected in developing ovaries and testes, and *hex 110 *was highly transcribed in the ovaries of egg-laying queens. A phylogenetic analysis revealed that HEX 110 is located at the most basal position among the holometabola hexamerins, and like HEX 70a and HEX 70c, it shares potential orthology relationship with hexamerins from other hymenopteran species.

**Conclusions:**

Striking differences were found in the structure and developmental expression of the four hexamerin genes in the honey bee. The presence of a potential binding site for Usp in the respective 5' UCRs, and the results of experiments on JH level manipulation in vivo support the hypothesis of regulation by JH. Transcript levels and patterns in the fat body and gonads suggest that, in addition to their primary role in supplying amino acids for metamorphosis, hexamerins serve as storage proteins for gonad development, egg production, and to support foraging activity. A phylogenetic analysis including the four deduced hexamerins and related proteins revealed a complex pattern of evolution, with independent radiation in insect orders.

## Background

Hexamerins essentially participate in the dynamics of amino acid storage and exploitation that occurs during insect development. These six-subunit proteins are primarily synthesized by the larval fat body and are massively stored in hemolymph as an amino acid source for development toward the adult stage [1]. They also may function as JH-binding proteins [2,3], and in addition, there is circumstantial evidence supporting the hypothesis that larval hexamerins are targeted for egg production [4-8].

While hexamerins have been the focus of numerous studies in solitary insects [9,10], the characterization of these proteins in social insects has received much less attention, in spite of the potential for discovering unique physiological functions linked to aspects of the social way of life. Workers of an ant species may use hexamerins as an amino acid source for brood nourishment, and there is circumstantial evidence that, by acting as a JH-binding protein, hexamerins regulate JH titer and caste differentiation in a termite species [11-15].

The highly eusocial honey bee hatches as a larva after a 72 h embryonic stage, and develops through a series of molts that define the five larval instars. This is a period of feeding, and the larva gains weight while it is continuously fed by worker bees. During the larval stage, queens, workers and drones have distinct nutritional requirements. Depending on the quality and quantity of nutrition, a diploid female larva develops as a queen or as a worker. A queen-destined larva is fed with secretions produced by worker hypopharyngeal and mandibular glands, the royal jelly, in a much higher proportion than a worker-destined larva. As a supplement to its nutritional regime, the worker larva also receives pollen, nectar and honey. Drone larval nourishment is composed of these same nutrients, but they are fed on a larger quantity of food, and their diet also differs in quality when compared to that given to workers [16]. Female and male larvae grow enormously because of these nutrients, and accumulate proteins, lipids and glycogen for use as structural materials and energy during the subsequent non-feeding pupal and pharate-adult stages. Duration of development from egg to adult eclosion differs considerably among queens, workers and drones, spanning 16, 21 and 24 days, respectively [17] with some differences among *A. mellifera *subspecies.

The single adult queen in the hive is adapted to egg production. When fertilized, the eggs will give rise to workers and occasionally to a new queen, while non-fertilized eggs become drones. The functionally sterile workers perform a series of flexible but age-correlated tasks, a phenomenon known as age polyethism. The younger worker bees usually stay inside the hive and are engaged in brood rearing, queen tending, nest building, nest cleaning, and food processing. Older workers take over the duties of foraging for pollen and nectar that are used to provision and maintain the hive. Drones do not have any known function other than mating with the queen [18].

Our goal was to determine whether these morphotypes (queen, worker, drone), which are so divergent in their developmental rate, size, morphology and other essential characteristics, and which perform very distinct functions as adults in the hive, also show hexamerin gene expression profiles that are correlated with their unique developmental trajectories. The current study was undertaken to deepen our knowledge of the four hexamerin genes found in the honey bee genome [19-21] by exploring their structures, expression patterns, and putative functions using a comparative approach. To this end we determined (1) the features of the full-length cDNA coding sequences and their conceptual translation products; (2) the potential regulatory sequences present in the respective 5' UCRs; (3) the expression patterns in the fat body and gonads of developing and adult queens, workers and drones; (4) the effect of JH on the expression in larval fat body; (5) the relative quantities of hexamerin transcripts in females and drones during the metamorphic molt and adult stage, and (6) the evolutionary relationships among the honey bee hexamerins and related members of the hemocyanin superfamily in other insect species. Consistent with the hypothesis that hexamerins have multiple functions in the honey bee, our findings disclosed striking structural differences among the hexamerin gene sequences and tissue-, caste- and sex-specific expression patterns. Additionally, the recognition of potential JH-target sites in 5' UCR of all hexamerin genes together with the observed JH-effect on the levels of hexamerin transcripts indicate regulation by this hormone.

## Results

### Structural characteristics of the hexamerin CDSs and respective translation products

**Figure 1:**
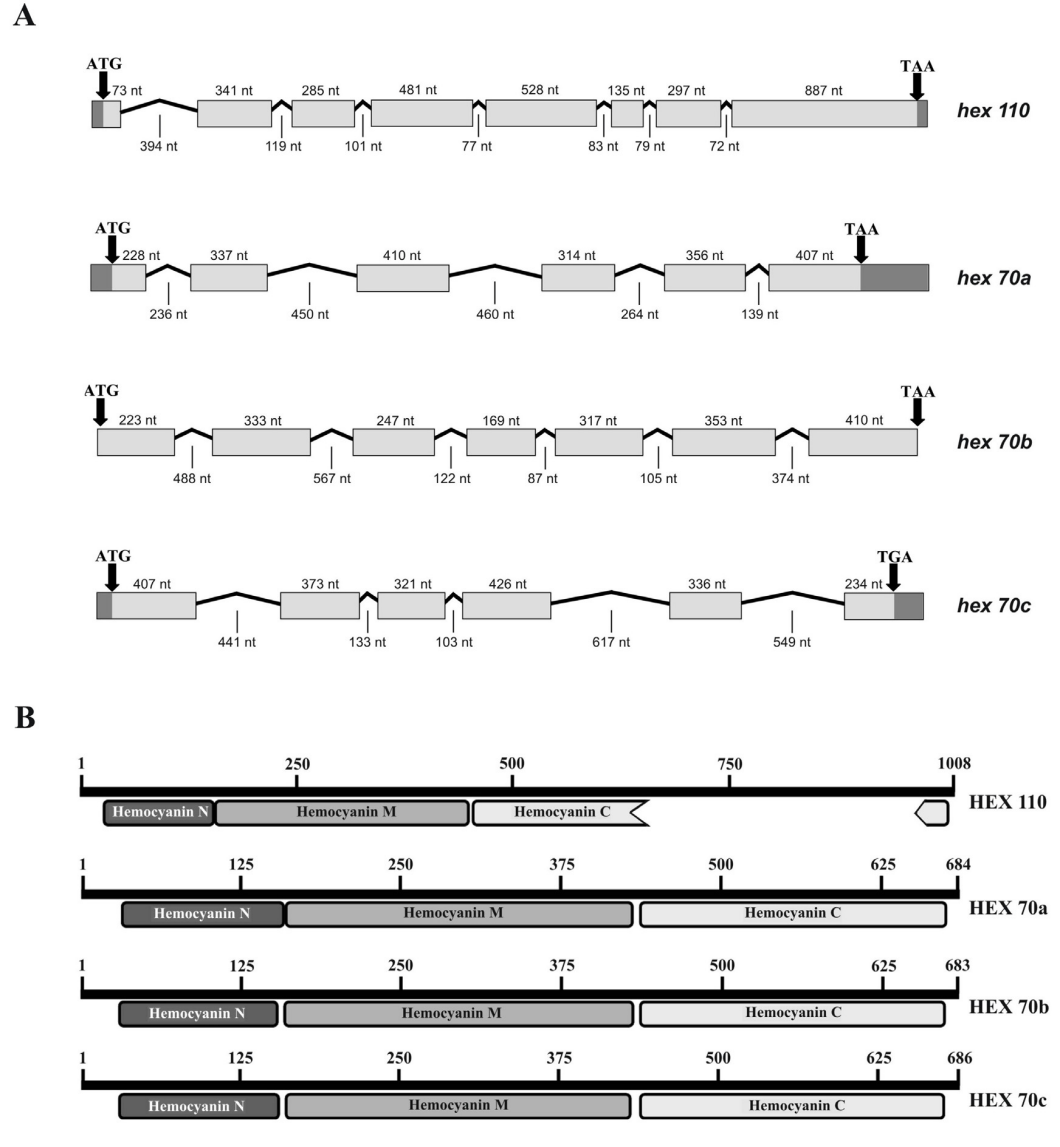
**Hexamerin genes and deduced protein subunits**. (A) Schematic diagram of hexamerin genes structure. Exons and introns are represented by boxes and lines, respectively. Arrows at the right and left of each gene indicate initiation and termination codons, respectively. Sequenced untranslated regions (UTR) are marked in dark gray. Number of nucleotides (nt) are indicated for exons and introns (exons and the primers used for hexamerin sequencing are marked in the nucleotide sequences shown in Additional files 1, 2, 3 and 4). (B) Diagrams of the deduced hexamerin proteins showing the N, M and C hemocyanin domains. Note that the C domain is interrupted in the HEX 110 sequence. Signal peptides and conserved motifs are marked in hexamerin sequences shown in Additional files 5, 6, 7 and 8.

The entire CDSs of *hex 70b *and *hex 70a*, as well as a portion of their respective 5' and 3' untranslated regions (UTRs), were previously sequenced by our research group [19,21]. Part of the *hex 110 *CDS (a cDNA fragment of 180 bp) also was previously cloned and sequenced in our laboratory [20]. In the current work, the sequencing of *hex 110 *was extended to the entire CDS and part of the 5' and 3' UTRs. In addition, we cloned and sequenced the *hex 70c *CDS as well as segments of its UTRs. Sequence analyses using the Artemis platform [22] allowed comparisons of the structural characteristics of *hex *CDSs (Figure 1A, Additional files 1, 2, 3 and 4). Each of these sequences is present as a single copy in the Honeybee Genome Assembly (version 4.0) as confirmed by BLAST searches. The *hex 70a*, *hex 70b *and *hex 70c *sequences are tandemly arrayed in GroupUn.53, whereas *hex 110 *is separately positioned in Group 11.32.

The translation products contain the N-terminal sequences determined by Danty et al [23] using automated Edman degradation. The conserved N, M and C hemocyanin domains (Figure 1B) were identified in all hexamerin subunits (HEX 70a, HEX 70b, HEX 70c and HEX 110), but as previously observed [20], the hemocyanin C domain of HEX 110 is interrupted by a 291 amino acid insertion. This insertion is very rich in glutamine and glutamic acid (Glx) and contributes significantly to the total Glx content (20.9%) of HEX 110.

Some features of the honey bee hexamerin genes and of the respective deduced subunits are compiled in Table 1. The four hexamerin subunits are also characterized by the presence of glycosylation sites, a conserved histidine and motifs typically found in other insect hexamerins (Additional files 5, 6, 7 and 8). We used the software http://phobius.sbc.su.se for prediction of signal peptides and transmembrane topology from the amino acid sequences of each of the hexamerin subunits in the honey bee, HEX110, HEX70a, HEX 70b and HEX 70c. Hydropathy profiles were produced for each of them and included in the Additional file 9. The subunits are predicted to contain each a signal peptide (Table 1, hydrophobic amino acids specified in Additional file 9) that directs transport of the protein through the secretory pathway. As expected, none of the subunits contain transmembrane helices. With respect to amino acid composition, HEX 70a and HEX 70c contain a relatively high quantity of phenylalanine, tryptophan and tyrosine (18.2% and 16.9%, respectively), and thus belong to the class of aromatic amino acid-rich hexamerins (or arylphorins) [24]. With their relatively high methionine content, HEX 70b (4.4%) and HEX 70c (6.4%) can be included in the class of methionine-rich hexamerins, which are composed of 4 to 11% methionine [10].

**Table 1:** Characteristics of hexamerin genes and respective subunits in the honey bee.

	Gene structure			Conceptual product		
	
Hexamerin genes/subunits	ORF (nt)	Exon number	Amino acid Number	Molecular mass (kDa)	pI	Signal peptide length
*hex 70a*/HEX 70a	2,055	6	684	79.19	6.45	21
*hex 70b*/HEX 70b	2,052	7	683	77.26	6.64	21
*hex 70c*/HEX 70c	2,061	6	686	79.44	7.67	19
*hex 110*/HEX 110	3,027	8	1008	110.21	6.37	24

Molecular mass and pI calculated using http://expasy.org/cgi-bin/protparam and http://phobius.sbc.su.se/

The overall amino acid identity shared among the deduced honey bee hexamerins varies from 30% to 42%. A multiple alignment using ClustalW 1.83 (Additional file 10) revealed that HEX 70a, HEX 70b and HEX 70c are more similar to each other (39 to 42% identity) than they are to HEX 110 (30 to 32% identity).

### Overrepresented motifs in upstream control regions (UCRs)

**Figure 2:**
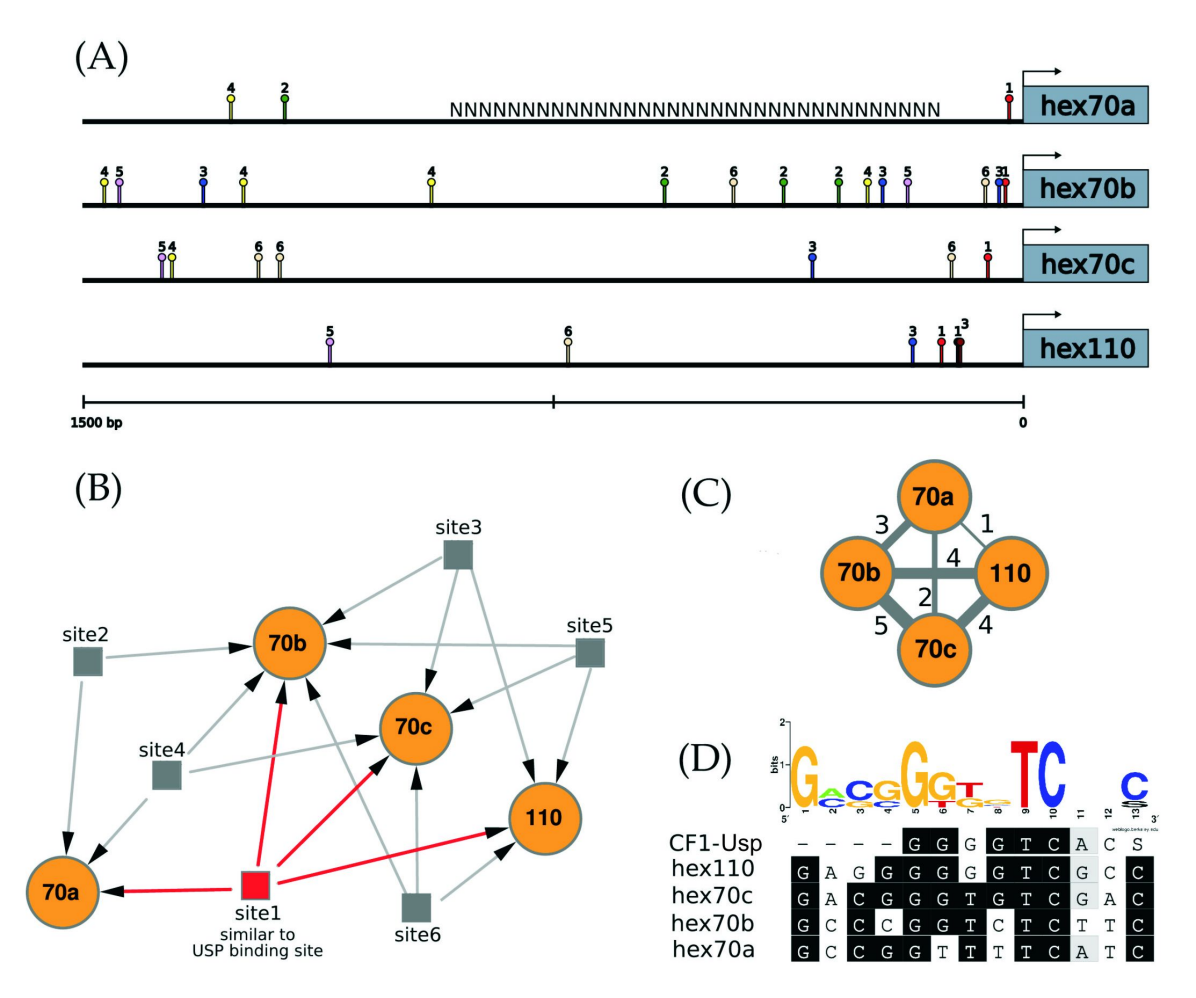
**Overrepresented motifs in the upstream control regions of the hexamerin genes**. (A) Spatial distribution of the six overrepresented DNA motifs in the 1.5 kb-long upstream control regions (UCRs) of the four honey bee hexamerin genes. Motif sequences are specified in Table 2. The N series in the *hex 70a *UCR indicates that this DNA region is still undefined in the honey bee genome. (B) Putative co-regulatory network showing the relationship between the six overrepresented motifs and hexamerin genes (orange circles). The site 1 motif (red square) is similar to the Usp binding element (also named CF1) in *D. melanogaster*. Sites 2 to 6 (grey squares) are not similar to any of the *D. melanogaster *binding site sequences described in the TRANSFAC database, and may be specific to hexamerin genes; (C) Based on the co-regulatory network, a scheme was constructed using the number of sites shared among hexamerin gene UCRs. The thickness of the bars linking hexamerin genes (orange circles) is directly proportional to the quantity of putative co-regulatory sites (the number of sites is indicated) shared by the four genes. (D) Alignment of the target sites similar to CF1-Usp found in the 5' UCR of honey bee hexamerin genes. The graph shows the sequence conservation at each position while the height of symbols indicates the relative frequency of each nucleotide at that position.

Motif analyses of the UCRs were carried out with two goals in mind: to search for potential JH response elements, and to search for hexamerin-specific conserved regions. Table 2 shows six DNA motifs, here named site1 to site6, that are overrepresented in the UCRs of the four hexamerin genes. All of the six motifs were mapped on an extension of the UCR corresponding to 1.5 kb from the translation start codon (Figure 2A). Site1 is 80% identical to the *D. melanogaster *Ultraspiracle (Usp) binding site, also known as chorion factor-1 (CF1, [Flybase ID: FBgn0003964]) [25], and is located very close to the 5' end (Figure 2A and 2D). Two site1 motifs enrich the *hex 110 *UCR, whereas only one was found in each of the UCRs of the three *hex 70 *genes. None of the other five motifs (site2 to site6) are similar to any binding site described to date in the TRANSFAC database, and may be specific to hexamerin gene UCRs. The *hex 70b *UCR showed the greatest complexity because it is the only one containing all six motifs. Few of these motifs were found in the *hex 70a *UCR, possibly because it is still a gapped DNA region (Figure 2A).

**Table 2:** DNA motifs discovered in the 5' UCRs of the honey bee hexamerin genes.

Motif name	Motif	Church	Roc-auc	P-value
site1	GnnGwnnmCsskC	9.3E-09	0.98	3.5E-06
site2	TTGyGngnnrrnAawk	3.8E-06	0.87	4.4E-04
site3	GsnsakwCgmnmG	3.9E-06	0.97	2.2E-04
site4	gwnAtnnnanwnwTCGAwAwys	3.8E-06	1.00	8.0E-07
site5	awCGAwAAAwnnknnwwRnA	9.3E-09	1.00	2.1E-05
site6	ATykCGAaTnmrannA	4.1E-06	0.99	9.5E-06

Site 1 motif is 80% similar to the USP binding site of *D. melanogaster*. The other motifs (sites 2 to 6) do not have similarity to any binding site sequences in TRANSFAC database, and may be specific of hexamerin genes.

Based on Figure 2A, we graphically represented the types (Figure 2B) and quantities (Figure 2C) of the potential regulatory sites shared by the four honey bee hexamerin UCRs. Site1 is shared by all four hexamerin genes; site2 is shared by *hex 70a *and *hex 70b*; site3 is present in *hex 70b*, *hex 70c *and *hex 110*; site4 is specific to the *hex 70 *genes (it is absent from the *hex 110 *UCR); and site5 and site6 were both detected in *hex 70b*, *hex 70c *and *hex 110 *(Figure 2B). Therefore, the *hex 70b *and *hex 70c *UCRs share a maximum of five of these motifs whereas the *hex 70a *and *hex 110 *UCRs share only one motif (Figure 2C). This relationship suggests that at least some of the hexamerin genes are co-regulated.

### Effect of JH on the expression of hexamerin genes

**Figure 3:**
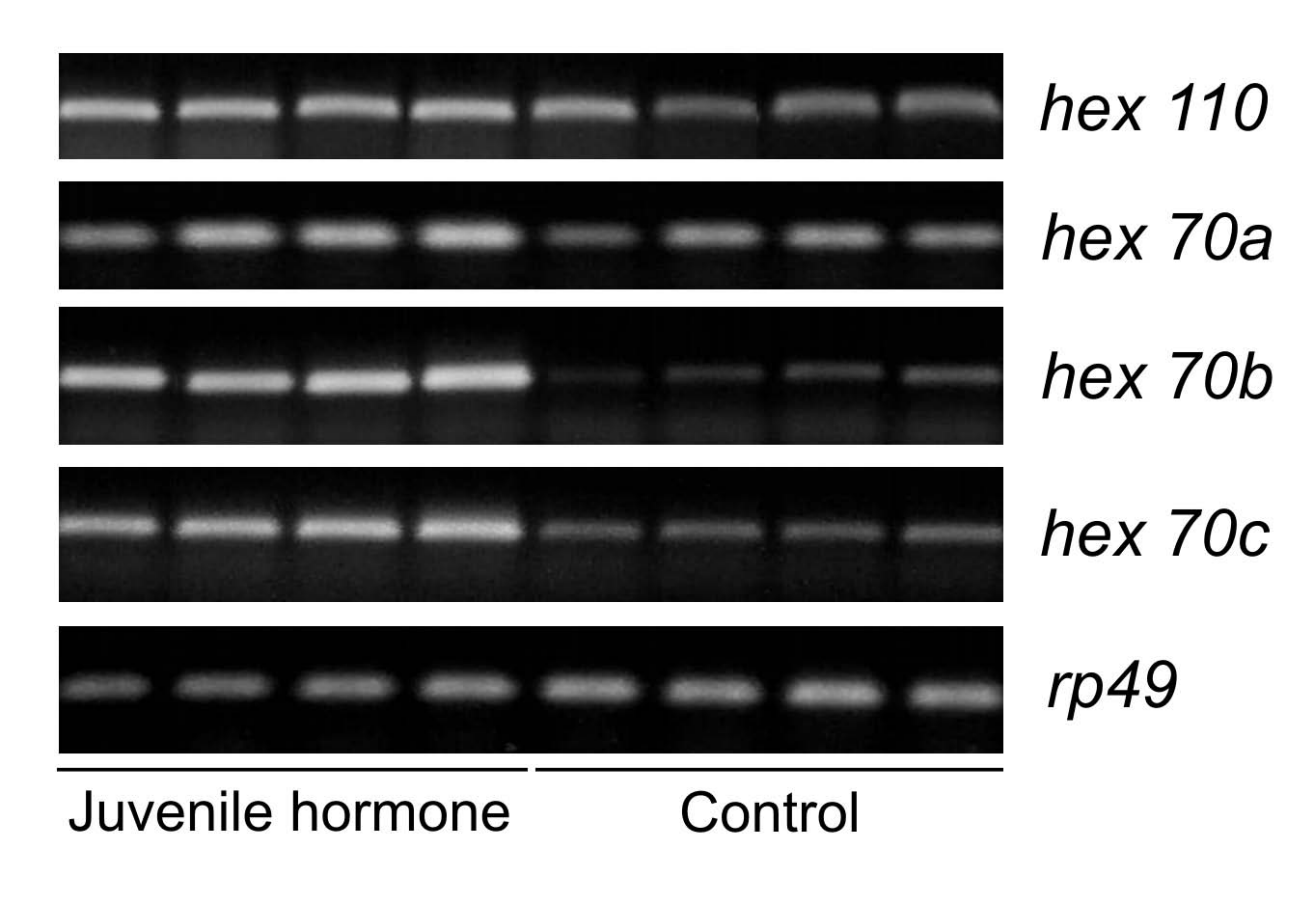
**Effect of juvenile hormone on the expression of the four hexamerin genes**. The hormone (diluted in acetone) was topically applied on the dorsum of 5^th ^instar larvae (feeding phase, L5F). Controls were treated with acetone only. Gene expression was analyzed 24 h after treatment. Hexamerin transcript abundance analyzed by RT-PCR followed by electrophoresis of the amplified cDNA on ethidium bromide-stained agarose gels. The *A. mellifera rp49 *gene was used as a loading control.

JH-treatment was performed at the feeding phase of the 5^th ^larval instar. During this developmental phase, larvae have a high titer of JH in hemolymph [26]. The treatment with exogenous hormone aimed to maintain JH titer at a high level for a prolonged period of time, thus circumventing the normal decay that normally occurs at the transition from the feeding to the spinning phase [26]. Figure 3 shows that expression of the genes *hex 70b *and *hex 70c *is higher in JH-treated larvae than in the controls, although the JH-effect on the expression of *hex 70a *and *hex 110 *is more discrete. Together, these results support a function of JH in inducing the expression of hexamerin genes in honey bee larvae.

### Evolutionary relationship among the honey bee hexamerins and related proteins

**Figure 4:**
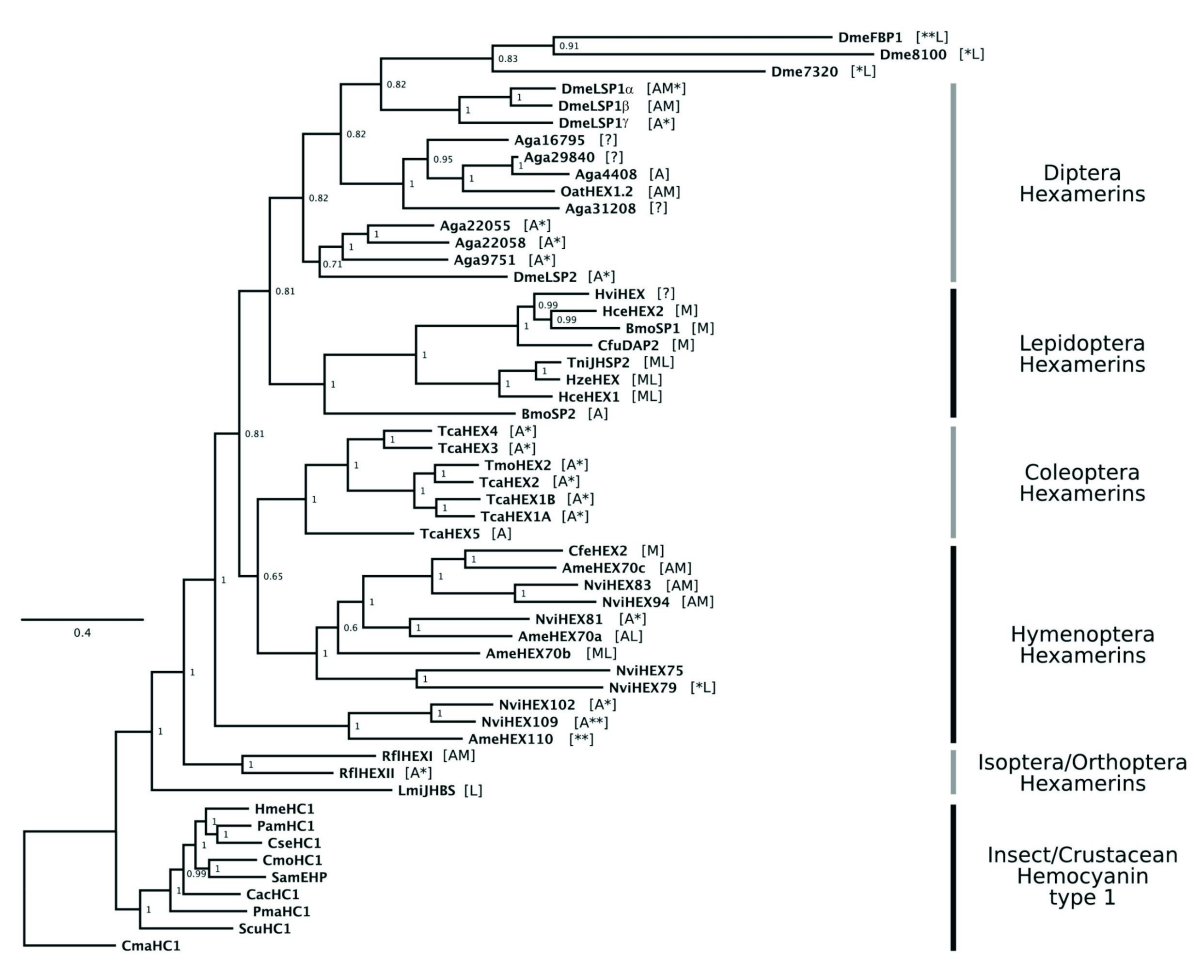
**Evolutionary relationships among insect hexamerins and hemocyanins**. The phylogenetic tree was inferred by bayesian method and the posterior probabilities are represented at each node. [See Additional data for the accession numbers of hexamerins and hemocyanins included in the tree (file 14), and for the alignment use in tree reconstruction (file 15)]. Symbols and letters in brackets indicate: ** very high-Glx content (>15%); * high-Glx content (between 10% and 15%); A: arylphorin; M: methionine-rich; L: leucine-rich; ? indicates that amino acid composition could not be determined. The first three letters of the protein's abbreviation represent the species: Cma:*Cancer magister; *Scu: *Sinella curviseta*; Pma:*Perla marginata; *Cac: *Chelidurella acanthopygia; *Sam: *Schistocerca americana*; Cmo: *Carausius morosus; *Cse: *Cryptotermes secundus; *Pam: *Periplaneta americana; *Hme: *Hierodula membranacea*; Rfl: *Reticuliformis flavipes*; Lmi: *Locusta migratoria*; Ame: *Apis mellifera*; Nvi: *Nasonia vitripennis*; Cfe: *Camponotus festinatus*; Tca: *Tenebrio castaneum*; Tmo: *Tenebrio molitor*; Bmo: *Bombyx mori*; Hce: Hyalophora cecropia; Hze: *Helicoverpa zea*; Tni: *Trichoplusia ni*; Cfu: *Choristoneura fumiferana*; Hce: *Hyalophora cecropia*; Hvi: *Heliothis virescens*; Dme: *Drosophila melanogaster*; Aga: *Anopheles gambiae*; Oat:*Ochlerotatus atropalpus*.

We investigated the evolutionary relationships among 45 hexamerin amino acid sequences from six insect orders (Hymenoptera, Diptera, Lepidoptera, Coleoptera, Isoptera, and Orthoptera), and hemocyanins from 8 insect species and from a crustacean. The tree structure mainly reflects the molecular relationship at the level of insect order (Figure 4). Within the hymenopteran cluster, the honey bee HEX 110 (named AmeHEX 110 in the tree) grouped with two hexamerins from *Nasonia vitripennis *wasps, NviHEX102 and NviHEX109. Like the honey bee HEX 110 (with ~110 kDa and 20.9% Glx), these wasp hexamerins have a molecular mass higher than that typically exhibited by hexamerins (102 kDa and 109 kDa, respectively) and are composed of a high or very high proportion of Glx (here defined as 10% to 15%, and >15%, respectively). Explicitly, NviHEX102 and NviHEX109 are composed of 14.2% and 15.1% Glx, respectively. The other hymenopteran hexamerins [NviHEX79, NviHEX75, NviHEX81, NviHEX94, NviHEX83; CfeHEX2; AmeHEX70a (HEX 70a), AmeHEX70b (HEX 70b), AmeHEX70c (HEX 70c)], which are in the range of 70-95 kDa, generally display an intermediary Glx content (7%<Glx<10%). Exceptions in this group are NviHEX79 and NviHEX81, which have higher amounts of Glx (10.1% and 11.8%, respectively).

Most of the hymenopteran hexamerins (seven of twelve) contain more than 15% aromatic amino acids, and are therefore considered arylphorins. Five of twelve meet the criterion for inclusion in the methionine-rich class. A wasp hexamerin (NviHEX79) and two of the honey bee hexamerins (HEX 70a, HEX 70b) contain more than 10% leucine and are here defined as leucine-rich.

Hexamerins from the coleopterans *Tribolium castaneum *and *Tenebrio molitor *form a well-defined group (Figure 4). Without exception, they are all arylphorins. Aside from TcaHEX5, they all have a high Glx content (at 9.8% Glx, TcaHEX5 fails to meet our criterion for inclusion among the high-Glx hexamerins by a small margin).

Among lepidopterans (Figure 4), the arylphorin BmoSP2 is positioned separately from the methionine-rich hexamerins HceHEX1, HzeHEX, TniJHSP2, CfuDAP2, BmoSP1 and HceHEX2, which are organized in two branches, one of them also including HviHEX. As only part of the HviHEX sequence is available in data Bank, we could not classify it as an arylphorin or a methionine-rich hexamerin. All lepidopteran hexamerins contain intermediary or low Glx (< 7%) content, and only three of them (HceHEX1, HzeHEX and TniJHSP) are rich in leucine. Interestingly, these leucine-rich hexamerins were grouped in a single branch.

A branch of dipteran hexamerins (Figure 4) included the very high Glx (20%)/high molecular mass DmeFBP1 and two other hexamerins from *D. melanogaster*, Dme7320 and Dme8100, both containing a high Glx content (13.7% and 10.8%, respectively), but a typical molecular mass ~70 kDa. All hexamerins in this branch are rich in leucine. The other three main branches consist of hexamerins in the range of 82-99 kDa. One of them clustered the DmeLSP1 isoforms (α, β and γ). The other two branches grouped some *Anopheles gambiae *hexamerins with OatHEX 1.2 from the mosquito *Ochlerotatus atropalpus*, and some *A. gambiae *hexamerins with DmeLSP2. Except for some incomplete sequences (Aga29840, Aga16795, and Aga31208) for which we could not determine the exact amino acid composition, all the hexamerins forming these three branches are arylphorins, and OatHEX1.2, DmeLSP1α and DmeLSP1β are also rich in methionine. Several of them have a high Glx content. None is rich in leucine.

The only orthopteran hexamerin used in tree construction, LmiJHBS (Figure 4), is distinguished by a high proportion of leucine. The basal position of this hexamerin is evident. Two isopteran hexamerins, RflHEX1 and RflHEX2 clustered in a single branch. Both are arylphorins, but RflHEX1 is also rich in methionine. RflHEX2, but not RflHEX1, has a high Glx content (10.4%).

As expected, the insect hemocyanins clustered separately from the hexamerins (Figure 4), and formed a well-supported monophyletic clade (1.0 Bayesian posterior probability), with ScuHC1 at the most basal position.

### Expression of hexamerin genes in the fat body of developing and adult workers, queens and drones

**Figure 5:**
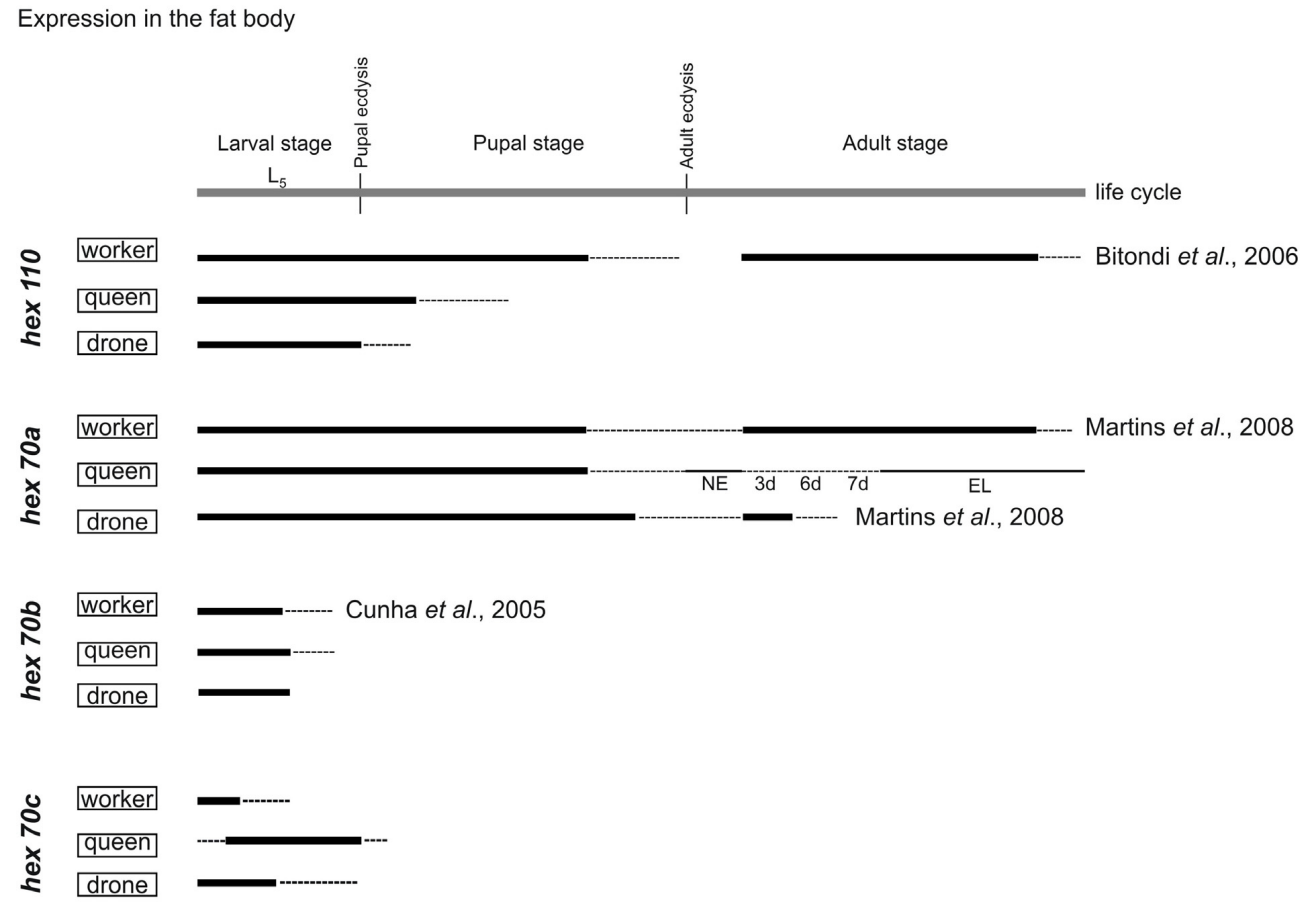
**Patterns of hexamerin gene expression in the fat body of immature and adult stages of workers, queens and drones**. The simplified diagrams represent expression patterns based on hexamerin transcript abundance as detected by RT-PCR followed by electrophoresis of the amplified cDNA on ethidium bromide-stained agarose gels using *A. mellifera *actin as a loading control (see Additional file 11). The thick, thin and dashed lines in the diagrams represent high, intermediary and low transcript levels, respectively. L5: 5th larval instar. L5F and L5S: feeding and spinning phases of the 5th larval instar. NE: newly emerged queens. EL: egg-laying queens. 3d, 6d, and 7d: adult age in days. Data previously published by our laboratory are indicated by the respective bibliographic reference.

Figure 5 schematically represents RT-PCR transcriptional profiles from the current and previous studies of the four hexamerin genes in the fat body of workers, queens and drones (see Additional file 11). Relative expression is shown from the 5th larval instar throughout the pupal and adult stages. Because the expression data on earlier larval phases (2nd to 4th larval instars) was mostly obtained using workers instead of queens and drones, they are not shown here.

In the fat body of workers, *hex 110 *transcripts were abundant from the 5th larval instar throughout the pupal stage, with a decrease in the amount of transcripts nearby the time of adult eclosion. But expression increased again in adult workers. In contrast, in the fat body of drones and queens, *hex 110 *expression was found basically in the 5th larval instar, extending up to the early pupal stage in queens. The expression of *hex 70a *differs from *hex 110 *mainly in adults, which showed sex- and caste-specific patterns of *hex 70a *transcription. Workers and drones showed high levels of *hex 70a *transcripts up to the ages of 30 and 5 days, respectively. In 3- to 7-day-old virgin queens, *hex 70a *expression was reduced in comparison to the newly emerged ones, but increased again in older, egg-laying queens. Comparatively, the expression of *hex 70a *is lower in adult queens than in adult workers. The expression of the other two hexamerin genes, *hex 70b *and *hex 70c*, was detectable only in the 5th larval instar of females and males.

Together, the data summarized in Figure 5 highlight that: (1) all the honey bee hexamerin genes are highly expressed in the larval fat body of workers, queens and drones, and (2) *hex 110 *and *hex 70a *were transcribed in a caste- and sex-specific fashion in pupal and adult fat body. These expression patterns suggest that in addition to their primary role as storage proteins that supply amino acids during non-feeding pupal development, hexamerins have different functions in the adult stage.

**Figure 6:**
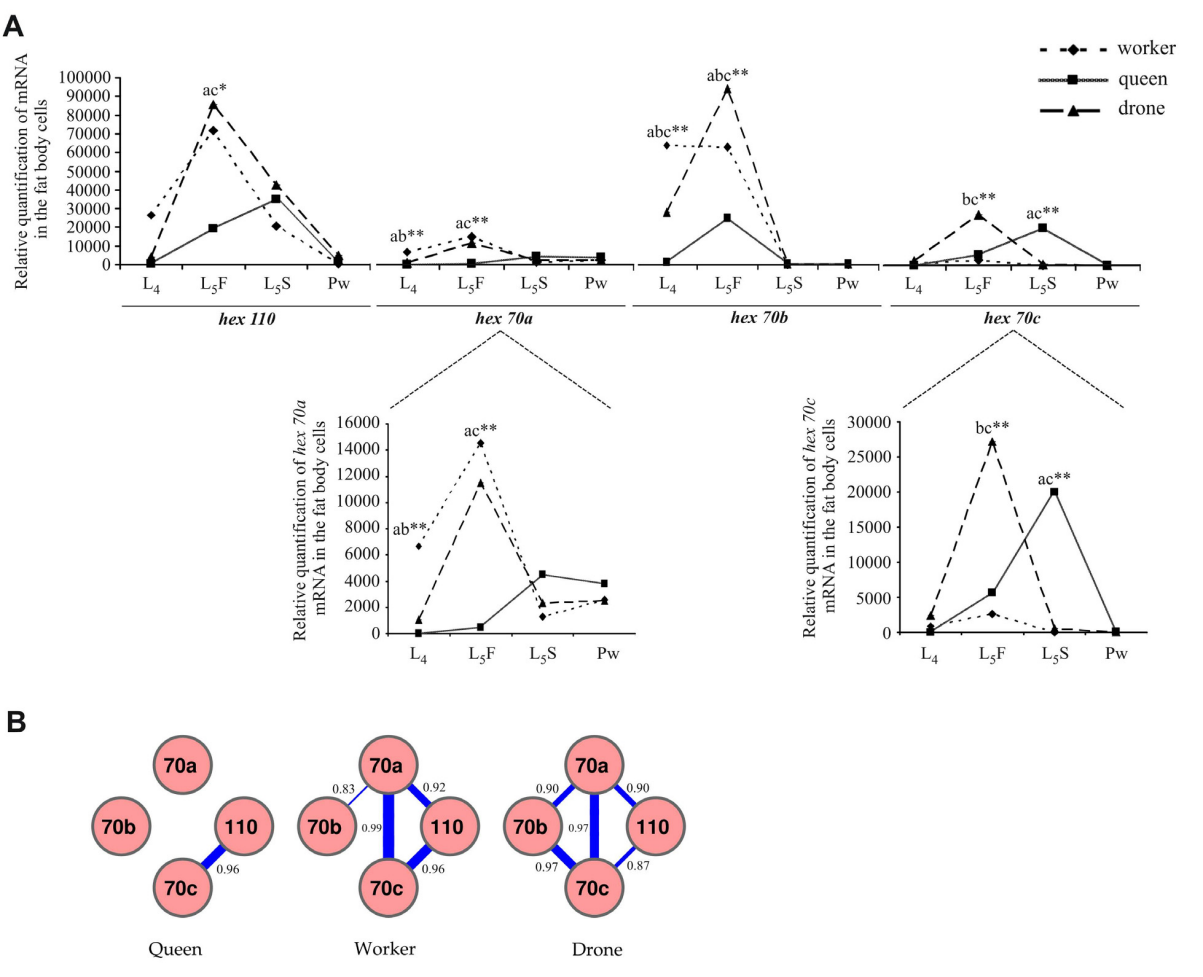
**Relative quantification of hexamerin transcripts in the larval and pupal fat body**. (A) Levels of *hex 70a*, *hex 70b*, *hex 70c *and *hex 110 *mRNAs measured by real time RT-PCR in workers, queens and drones during the 4th larval instar (L4), the feeding (L5F) and spinning (L5S) phases of the 5th larval instar and in newly ecdysed pupae (Pw). All points in the curves represent means and standard errors of three biological samples each prepared twice (experimental replicates). For a better visualization of the data, the Y axis scale was extended in the graphs representing *hex 70a *and *hex 70c *expression. Letters indicate significant differences: (ab) workers ≠ queens/drones; (ac) queens ≠ workers/drones; (bc) drones ≠ workers/queens; (abc) workers ≠ queens ≠ drones. Statistical analysis was carried out with Jandel SigmaStat 3.1 software (Jandel Corporation, San Rafael, CA, USA), using two way ANOVA with post-hoc comparisons by the Holm-Sidak multiple comparison test. * p = 0.04, ** p ≤ 0.001. (B) Correlation among transcriptional profiles of the four honey bee hexamerin genes in queens, workers and drones during larval-pupal transition. The greater the thickness of the bar linking the encircled hexamerin genes, the higher the Pearson's correlation coefficient and association among expression profiles.

Using real-time RT-PCR we quantified the levels of the four hexamerin transcripts in the fat body at two periods of the honey bee life cycle: during larval-pupal transition and in adults. Figure 6A shows that the transcriptional profiles are similarly modulated, with an abrupt increase in the quantity of transcripts in the 5th larval instar, followed by a marked decrease in newly ecdysed pupae. However, at definite points of this developmental period, we found interesting differences among the honey bee morphotypes. In workers and drones, the four hexamerin transcripts reached maximal levels during the feeding phase of the 5th larval instar (L5F). In queens, maximal transcript levels were detected at the subsequent spinning phase (L5S) (except for *hex 70b *transcripts, which reached maximal levels in L5F). Moreover, the maximal expression in workers and drones was significantly higher than the maximal expression in queens (except for *hex 70c*). Figure 6A also shows that in workers and drones at the L5F phase, when expression of the four hexamerin genes reached a maximum, the levels of *hex 110/hex 70b *transcripts were much higher than the levels of *hex 70a/hex 70c *transcripts.

Pearson's correlation coefficient (R) was used to evaluate the relationship among these expression profiles. Figure 6B graphically represents the hexamerin genes (orange circles) linked by bars. The greater the thickness of the bar, the greater the R value. In queens, the expression profiles of *hex 110 *and *hex 70c *were the only positively correlated. In drones, by contrast, the expression profiles of all the hexamerin genes except *hex 70b*/*hex 110 *were positively correlated. Similarly, in workers the expression profiles of all but *hex 70b*/*hex 110 *and *hex 70b*/*hex 70c *were positively correlated. Therefore, workers and drones share similar transcriptional profiles during the larval-pupal transition that are distinct from those exhibited by queens. In other words, workers and drones differ from queens in the patterns of co-expression of the four hexamerin genes.

**Figure 7:**
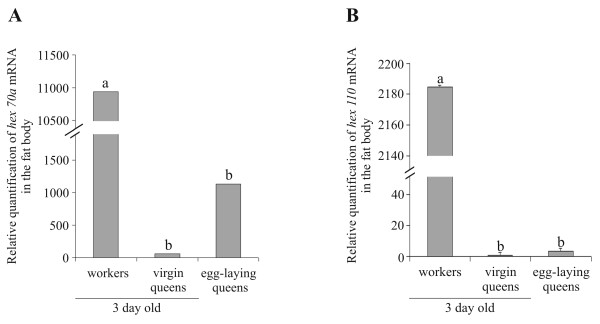
**Relative quantification of hexamerin transcripts in the fat body of adult females**. Levels of **(A) ***hex 70a *and **(B) ***hex 110 *transcripts in 3-day-old workers and virgin queens, and in egg-laying queens. Different letters indicate significant differences between groups (p ≤ 0.001; one-way ANOVA with post-hoc comparisons by the Holm-Sidak test; SigmaStat 3.1 software; Jandel Corporation, San Rafael, CA, USA).

Fat body from adult females was also used to quantitatively compare the levels of *hex 70a *and *hex 110 *transcripts (the only ones found at this stage). We compared age-matched workers and queens (3-day-old), but to investigate the effect of mating in transcript levels, we also included egg-laying queens in this analysis. Figure 7 shows that 3-day old workers have a significantly higher quantity of both transcripts than queens, independent of their reproductive status.

### Expression of hexamerin genes in the gonads of developing and adult workers, queens and drones

**Figure 8:**
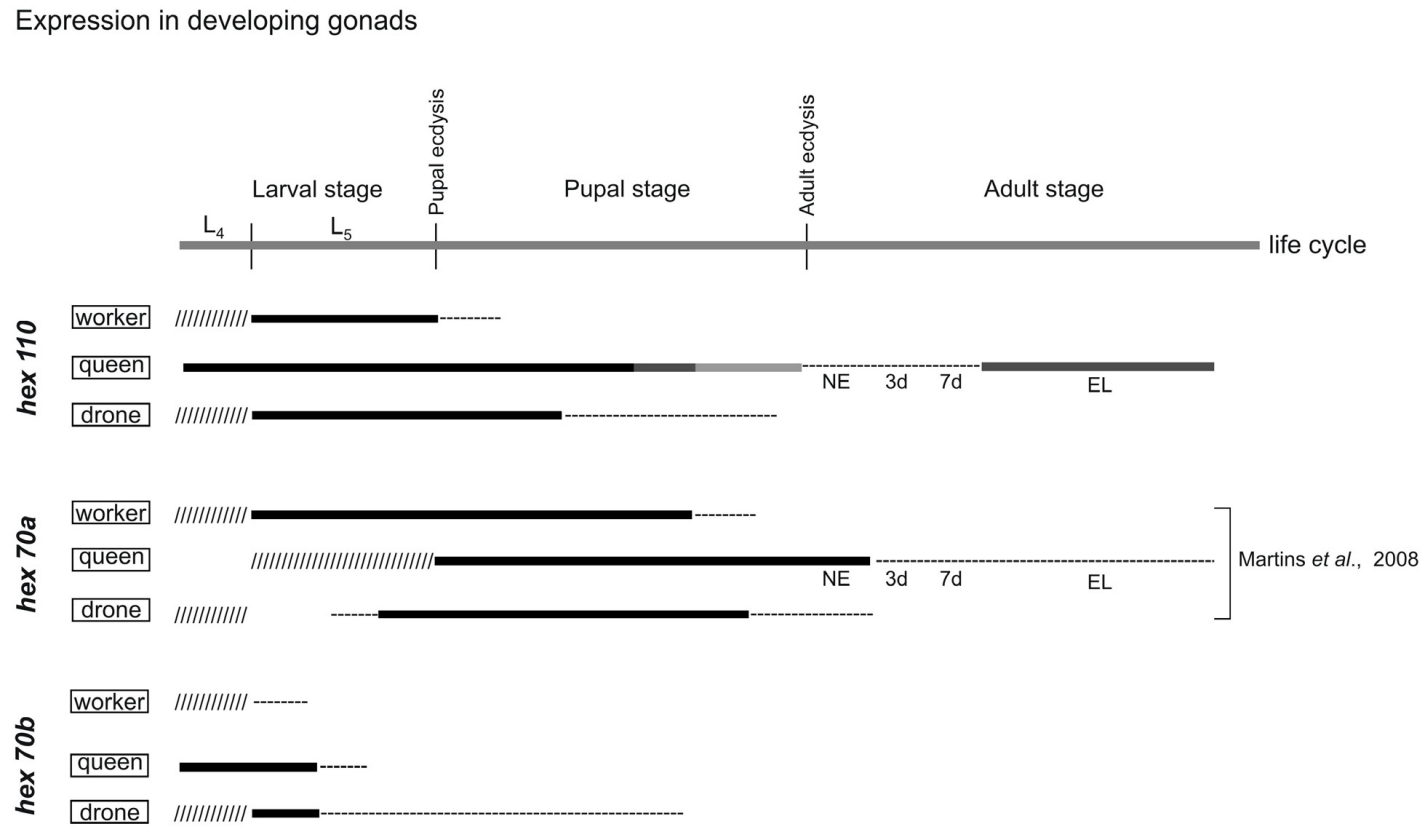
**Patterns of hexamerin genes expression in the gonads of immature and adult stages of workers, queens and drones**. The simplified diagrams represent expression patterns based on the hexamerin transcript abundance as detected by RT-PCR followed by electrophoresis of the amplified cDNA on ethidium bromide-stained agarose gels using *A. mellifera *actin as a housekeeping gene (see Additional file 12). The black, dark-grey, light-grey and dashed lines in the diagrams represent high, intermediary, low and very low transcript levels, respectively. Data not shown are indicated (/////). L4 and L5: 4th and 5th larval instars. NE: newly emerged queens. EL: egg-laying queens. 3d and 7d: adult age in days. Data previously published by our laboratory are indicated by the respective bibliographic reference.

The expression of hexamerin genes was also investigated in developing and adult female and male gonads using RT-PCR (Figure 8; see Additional file 12). The only hexamerin gene apparently inactive in ovaries and testes is *hex 70c*, as presumed from the complete absence of its transcript in these organs. The levels of *hex 70b *transcripts were abundant, but only in the larval gonads of queens and drones. A high level of *hex 110 *mRNA was found in the larval gonads of workers, queens, and drones. Expression then decreases during the pupal stage to be resumed exclusively in the ovaries of egg-laying queens. Similarly, a relatively high level of *hex 70a *transcripts was found in the gonads of workers and drones at the larval/pupal stages, and in the ovaries of queens at the pupal/early adult stages. This is followed by transcript depletion in the gonads of workers and drones, but not in the ovaries of virgin and egg-laying queens where *hex 70a *expression is maintained, although at a low level, throughout the adult stage.

In summary, the presence of hexamerin transcripts in larval and pupal gonads of workers, drones and queens suggests roles in ovary and testis development, and in spermatogenesis, which occurs during the pupal stage. The higher expression of *hex 110 *in the ovaries of egg-laying than virgin queens is remarkable, and suggests a function in reproduction.

## Discussion

### Hexamerin genes and deduced proteins revealed striking structural differences

The tandem organization of the *hex 70a*, *70b *and *70c *genes in the honey bee genome supports the hypothesis of origin by gene duplication, a common phenomenon among insect hexamerins [27]. The separately located *hex 110 *gene exhibits unusual features throughout its sequence. It encodes a subunit that is longer than those usually found, and which carries a very high proportion of Glx (20.9%). Because these features are also displayed by hexamerins found in some species of ants [28] and wasps [29], it had been previously thought that they were restricted to hymenopterans. In fact, two hexamerins in the *N. vitripennis *wasp (NviHEX102 and NviHEX109) exhibit such characteristics (see Figure 4). However, a receptor in *Drosophila*, DmFBP1 (which is closely related to its own ligand DmLSP1) [30], is also composed by a very high Glx content (20%) and has a high molecular mass (116 kDa). We also identified hexamerins containing slightly lower Glx percentages (between 10% and 15%) among the dipterans, coleopterans and in the isopteran included in the tree (see Figure 4). The physiological significance of such a high proportion of glutamine and glutamic acid in hexamerins remains to be elucidated.

A conserved histidine residue was identified in each of the four amino acid sequences. In the ancestral hemocyanins, six copper-liganding histidines confer the ability to bind and transport oxygen. The insect hexamerins lost most or all of the histidine residues and, thus, the oxygen-binding function [31].

The ~70 kDa hexamerins in the honey bee were classified as arylphorins and/or methionine-rich, according to their particular amino acid composition. Due to the importance of aromatic amino acids in sclerotization, the HEX 70a and HEX 70c arylphorins may contribute to exoskeleton hardening and differentiation during pharate adult development. HEX 70c is also rich in methionine, and like HEX 70b (also a methionine-rich hexamerin) it may act as a sulfur reserve for development toward the adult stage.

The search for *cis*-acting elements in the UCR of each hexamerin gene using bioinformatic analyses revealed a total of six overrepresented DNA motifs. It was very interesting to find out that all four hexamerin genes exhibit potential binding sites for the protein Usp, thus suggesting regulation by JH and/or ecdysteroids. Usp has been primarily studied as part of the 20-hydroxyecdysone (20E)-binding nuclear receptor complex [32,33]. However it was recently also identified as a potential target for compounds based on a methyl farnesoid structure, like JH [34]. The potential Usp binding motif is located near the start codon of each hexamerin gene (see Figure 2A), a conserved pattern that increases the likelihood of its functionality. Using a hormone manipulation experiment, we recently demonstrated that *hex 70b *is induced by JH and repressed by 20E [19]. In the current study we expanded this experiment to investigate the action of JH on the expression of the other honey bee hexamerin genes. To make this approach comparative, we re-tested the expression of *hex 70b *in the same fat body samples used for the analysis of the other genes. The results showed a strong and positive JH-influence on the expression of *hex 70b *and *hex 70c*, and a weaker effect on the expression of *hex 70a *and *hex 110*. The observed differential effect of JH on expression levels suggests that the JH titer that induces a hexamerin gene for maximal expression may differ from the JH-threshold needed for maximal expression of their homologous. Further studies using a series of JH doses for treating aged-synchronized honey bee larvae may confirm (or refute) this hypothesis. These results are in accord with the proposed functionality of the Usp regulatory element in UCRs.

In addition to the potential Usp binding motif, five additional motifs (site2 to site6) were overrepresented in the UCRs of all four hexamerin genes, suggesting co-regulation. In the context of metamorphosis, or larval-pupal transition, this would assure the massive and synchronized production of hexamerins in late larvae for using during pupal and pharate-adult development. In fact, all four hexamerin genes reached their highest expression level during the very same stage, the 5th larval instar (see Figure 6A). Lending support to the idea of co-regulation, we found that the transcriptional profiles of the hexamerin genes in each caste and sex during the larval-pupal transition are in general positively correlated. Regulatory factors could interact with the common sequence motifs, thus influencing the correlated expression. However, the transcriptional profiles of *hex 70b/hex 110 *in workers and drones, and of *hex 70b/hex 70c *in workers, did not show a positive correlation (see Figure 6B). Furthermore, in metamorphosing queens, only the *hex 70c *and *hex 110 *transcriptional profiles showed a significant Pearson's correlation coefficient. Such differences might be due to structural features in the architecture of each hexamerin gene UCR, such as the type, number and spatial distribution of the overrepresented motifs, which raises the possibility of nuanced differences in the co-regulatory mechanism.

### Diversification of hexamerins associated with independent radiation within insect orders

Based on amino acid sequence similarities and hexameric structure, it has been proposed that arthropod hemocyanins gave rise to the insect hexamerins [31,35], which lost the ability to bind Cu2+ ions. Therefore, in contrast to the ancestral molecule, hexamerins do not bind and deliver hemolymph oxygen, but mainly have a role as storage proteins. The molecular phylogeny shown in Figure 4 shows a complex pattern of hexamerin evolution, with independent radiation in each of the selected insect orders. In general, our analysis resulted in a similar tree topology as those published before [36], with the hemimetabola (orthopteran and isopteran) hexamerins in a basal position, followed by hymenopteran and coleopteran hexamerins, which are basal to the lepidopteran and dipteran ones. As previously noticed, these evolutionary relationships are in good agreement with phylogeny of insect orders [37].

Most of the information on hexamerin evolution derives from studies on dipteran and lepidopteran species, with a few studies on other holometabolous and hemimetabolous orders, although data from an evolutionarily less-derived order (Plecoptera) have been recently published [38]. The main contribution of the current phylogenetic analysis was to explore the evolutionary relationship among the honey bee hexamerins and their homologue sequences in other insect orders. It is evident in our phylogenetic tree that the high molecular mass AmeHEX110 is located at the most basal position among the holometabola hexamerins. AmeHEX110 shares this position with two probable orthologues, NviHEX102 and NviHEX109. Similarly, the gene encoding AmeHEX70a apparently has an orthologue, NviHEX81, in the genome of *N. vitripennis*. Other potential orthology relationship was exhibited by the honey bee AmeHEX70c, and the ant CfeHEX2.

The molecular phylogeny also revealed a potential orthology relationship outside the hymenopteran clade, between the hexamerins from *T. molitor*, TmoHEX2, and *T. castaneum*, TcaHEX2, but a definitive conclusion would only be possible if *T. molitor *had its genome sequenced. For the lepidopteran hexamerin sequences it is more complicated to infer orthology relationships, at least until they were available in public databases. Orthology may be a reflection of functional equivalence, although it is important to consider that in view of gene loss and other events in evolution of a group of organisms, genes from different species sharing high sequence similarity may be not orthologues at all [39]. Functional studies are therefore decisive to elucidate this complex relationship among hexamerin genes.

A relevant observation taken from our molecular phylogeny is the well-supported split between dipteran hexamerins and the group formed by the hexamerin receptor DmeFBP1 and Dme8100 plus Dme7320 (0.82 posterior probability; Figure 4). It has already been suggested that dipteran hexamerin receptors, which also belong to the hemocyanin-hexamerin superfamily, form a separate group in phylogenetic trees [37]. Whether these other two DmeFBP1 paralogs have a similar function is still unknown.

It is also obvious that most of the lepidopteran hexamerins used in tree reconstruction are methionine-rich proteins, thus suggesting that they derived from an ancestral molecule containing a high proportion of this amino acid. We also observed that, except for AmeHEX110 every basal hexamerin in each insect order is an arylphorin, thus suggesting that insect hexamerins have evolved from an ancestral arylphorin. Our phylogenetic analysis also supports the hypothesis of gene duplication events taking place mainly after the split of insect orders.

### Expression of hexamerin genes in larval fat body: the well-known role in metamorphosis and a putative role in binding JH and regulating caste differentiation

The four hexamerin genes were highly transcribed in the larval fat body of workers, queens and drones, and are probably involved in hexamerin synthesis for amino acid storage to support metamorphosis and development toward the adult stage. However, these genes were differentially co-expressed in the honey bee morphotypes, as revealed by transcript quantification during the critical period of larval-pupal transition. The most evident difference occurred during the feeding phase of the 5th larval instar (L5F), where we observed a higher level of *hex 110*, *hex 70a *and *hex 70b *transcripts in workers and drones than in queens. This is a very interesting finding if contrasted to the caste and sex-specific JH titer at this stage, which is higher in queens than in workers and drones [26,40]. It is known that JH plays a central role in phenotypic caste differentiation in *A. mellifera*. This hormone triggers specific physiological responses in the bipotent female larvae, a high titer inducing development of a queen and a low titer specifying the worker phenotype [41,42]. The inverse relationship between levels of hexamerin transcripts and JH titers leads us to speculate that, as was proposed for the termite *Reticulitermis flavipes *[14,15,43,44], the honey bee hexamerins may function as JH-binding proteins. By binding and controlling JH levels, hexamerins were implicated in the process of JH-dependent caste differentiation in this termite. By applying this model to the honey bee, we may hypothesize that if JH titer exceeded the binding capacity of hexamerins, it would interact with target receptors and therefore cause the bipotent female larva to develop as a queen. If not, the larva would develop as a worker. Interestingly, all the four hexamerin genes showed a significantly higher expression in drone feeding larvae (low JH titer) than in queens at the same stage (high JH titer), thus reinforcing our hypothesis on a role for the honey bee hexamerins in binding and controlling JH action. But this needs further validation.

To our knowledge, hexameric JH-binding proteins have been characterized only in orthopterans [2,3,45]. The cDNA and predicted amino acid sequences of the high affinity JH-binding hexamerin from *L. migratoria *(LmiJHBS) show peculiar structural features. It is clear that LmiJHBS is not closely related to any other hexamerin, including the potential JH-binding hexamerin from *R. flavipes *(RfHEX1) and the honey bee hexamerins (see Figure 4). The lack of homology clearly indicates that these hexamerins have evolved independently, and it is unlikely that comparisons among their primary sequences will bring to light amino acid regions or domains that could be involved in JH binding. Such regions or domains could not be identified in the LmJHBS sequence [3]. Thus, functional binding assays and determination of dissociation constants are required to assess the potential role of hexamerins in binding JH and, by extension, to verify the role of the honey bee hexamerins in caste determination.

An interesting finding is that insect hexamerins may not directly bind JH but may be part of a multiprotein complex engaged in JH sequestration and transport. The first demonstration of a physical interaction among JH-binding proteins (both free and in a complex with JH), two hexamerins (one of them is an arylphorin), and an apolipophorin was recently provided [46]. This interaction implies an important participation of hexamerins in regulating JH levels, and action, even if they do not directly bind to JH.

### Re-induction of hexamerin gene expression in the adult fat body: differential roles in workers and queens

In agreement with previous reports on hemolymph proteins in the honey bee [23,47], lower levels of hexamerin gene expression were found in adult workers in comparison to larvae. Of the four hexamerin genes, *hex 70a *and *hex 110 *were the only ones with detectable expression in the adult fat body. Their transcriptional profiles differed conspicuously among adult queens, drones and workers. Levels of *hex 70a *transcripts were very low in queens and abundant in drones and workers. However, in drones, the abundance is limited to the five first post-emergence days, whereas, in workers, it is extended up to the 30th day of adult life. These transcriptional profiles in workers and drones match the respective HEX 70a profiles in hemolymph [21], and are closely connected with adult life duration. Workers live longer than drones [48,49], and their respective patterns of *hex 70a *expression reflects fat body metabolic activity and lifespan. But the higher expression of *hex 70a *in the fat body of adult workers in comparison to queens (see Figures 5 and 7) suggests an important role in worker physiology. This is discussed below.

The expression of *hex 110 *is somewhat different when compared to that of *hex 70a*. Like *hex 70a*, the *hex 110 *gene is considerably active in the fat body of adult workers, but it is practically silent in queens (see Figures 5 and 7) and drones (see Figure 5). Yet, in spite of the evident presence of *hex 110 *transcripts in adult workers, a negligible amount of HEX 110 subunits were detected in hemolymph [20], indicating that this hexamerin does not function as a storage protein at this stage. But a potential function in the fat body of adult workers cannot be ruled out, given its specific and abundant expression in this tissue.

Our results are consistent with re-induction of *hex 70a *and *hex 110 *expression in adult bees, although only the product of *hex 70a *accumulates in hemolymph at this stage. The presence of hexamerins in adult insects may occur either because they were carried over from the larval stage, or due to a specific induction in adults. As examples, some lepidopterans that do not feed on protein as adults may use the larval store of hexamerins for egg production [5]; mRNAs for an adult-specific hexamerin appears in *Musca domestica *females only after induction by a rich protein meal [50]. Differently, the presence of HEX 70a in the adult honey bee results from gene re-induction after a drastic reduction in transcript levels during adult ecdysis (see Figure 5).

The honey bee queen continuously receives, via trophallaxis, a proteinaceous glandular secretion, the royal jelly, which is produced by nurse workers [51]. She may not need to allocate amino acids from larval hexamerins for egg production, since such compounds are continuously derived from her protein-enriched diet. The structural nutrients and energy contained in royal jelly administered to queens are not used for the synthesis and storage of hexamerins (levels of hexamerin transcripts are very low, or undetectable, in the fat body of adult queens; see Figures 5 and 7) but must be mainly directed to vitellogenesis and egg production. In contrast to the queen, workers normally do not produce eggs, although they also have access to a rich source of dietary proteins. They actively consume pollen when they are younger, i.e., during the first two weeks of adult life [52]. Pollen consumption increases the expression of *hex 70a *and *hex 110 *in the fat body, and increases the abundance of HEX 70a in hemolymph [20,21]. This is consistent with dietary protein consumption causing reinduction of both hexamerin genes in adult workers, and thereby enabling the storage of HEX 70a. Whether *hex 110 *mRNAs are translated and, if so, why their subunits do not accumulate in hemolymph are questions that remain unanswered.

Pollen consumption also causes the accumulation of vitellogenin, the yolk protein precursor, in the hemolymph of young workers [53]. Vitellogenin is continuously produced and stored in hemolymph during the first two weeks of adulthood to be subsequently depleted as worker bees get older and become foragers [54-57]. HEX 70a follows a similar pattern.

Since workers normally do not reproduce and in general have a short life, the question that remains unanswered is: why do they store proteins? We suggest that the consumption of pollen by young workers exceeds demand, and the excess is hoarded in the form of storage proteins to be consumed later, when they become foragers. Foragers rather eat nectar [58], which is composed primarily by carbohydrates [59]. By this means, stored proteins could provide amino acids for sustaining worker basal metabolism during foraging. The well documented observations that HEX 70a [21], vitellogenin [55-57], and total hemolymph protein titers [60] decrease gradually in foragers is consistent with this hypothesis.

The destination of proteins stored in worker hemolymph would not be fixed, but dependent on the social context. In case there is queen loss, workers may activate their ovaries for drone production. Their protein reserves would then be directed to meet reproduction demands. Interestingly, workers accumulate storage proteins when they are younger and more prone to activate their ovaries if separated from the queen. Also in the ant *Camponotus festinatus*, hexamerins are possibly involved in important facets of sociality. It was demonstrated that in the presence of larvae, adult workers do not store, but apparently make use of hexamerins to nourish larvae. Conversely, hexamerins accumulate in hemolymph of workers with no larvae to feed. It was also observed that hexamerins exist in great amounts in virgin queens and are depleted when they seal themselves in a chamber to lay eggs to found a new colony, thus indicating utilization for egg production and rearing the first brood [11,12]. The high levels of hexamerins stored by certain species of termites were also found to be related to colony founding and to the production of initial broods [13].

In adult honey bee workers, vitellogenin and HEX 70a may be part of the above mentioned multiprotein complex engaged in JH binding and sequestration [46], since the presence of both in hemolymph coincides with a low JH titer (in younger workers), and their depletion occurs in synergy with JH titer increase (in foragers). If so, as proposed for vitellogenin [61], HEX 70a also may be a player in the physiological process of JH-regulated transition to forager.

Yet, HEX 70a may have developed another role in adult workers. A protein fragment found in the honey bee venom [62] matched HEX 70a N-terminal sequence. Its function in the venom, and whether it is synthesized by the venom gland or sequestered from hemolymph, was not yet determined.

### Hexamerin gene expression in gonads: a proposed role in ovary and testis development and activity

Among solitary insects there is circumstantial evidence supporting the hypothesis that hexamerins are also used for reproduction. In lepidopteran species, for example, it was established a correlation between egg production and depletion of the larval reserve of hexamerins [4,5]. Autogenous mosquitoes that produce their first batch of eggs without a feeding may use larval storage proteins, mainly hexamerins, as amino acid source for this purpose [6,7]. It has also been suggested that amino acids held in storage proteins are used for provisioning eggs of *Schistocerca americana *[8].

To shed light on whether the "adult" honey bee hexamerins are important or not for reproduction, we checked for the presence of transcripts in the gonads. Except for *hex 70c*, hexamerin transcripts were abundant in the gonads of larvae and pupae, suggesting roles in ovary differentiation, and also in spermatogenesis, which in drones occurs during pupal stage and is finalized before adult emergence. Two of the hexamerin genes, *hex 70a *and *hex 110*, were also expressed in adult gonads, and this was exclusively in queen ovaries. Interestingly, the expression of *hex 110 *is higher in the ovaries of mated, egg-laying queens than in the young, virgin ones.

It is known that mating triggers changes in gene expression in *Drosophila *females [63]. Mating also elicits physiological and behavioral changes in honey bee queens, and although the molecular mechanisms underlying such responses are largely unknown, it was verified that they involve differential gene expression into the ovaries. Based on gene ontology annotation, a function in oogenesis and reproduction was attributed to these differentially expressed genes [64]. The high expression of *hex 110 *in the ovaries of egg-laying queens suggests a role linked to ovary activity and reproduction.

Ovaries of egg-laying queens have a lower level of *hex 70a *transcripts than ovaries of newly emerged ones. But HEX 70a subunits exist in equivalent amounts in the ovaries of both, newly emerged and egg-laying queens, as confirmed by Western blots using a specific antibody [21]. Therefore, ovarian HEX 70a molecules seem to have a dual origin. It is produced by the fat body of egg-laying queens (see Figure 5) and secreted in hemolymph [21,23], implying that it could be incorporated into the ovaries in addition to being synthesized by them. The function of HEX 70a in the ovaries of virgin and egg-laying queens is to be determined.

## Conclusions

Our study revealed dramatic differences in structure, organization and expression of the four hexamerin genes of the honey bee, where these differences might have arisen concurrently with their functional diversification. The amino acid composition, motifs and conserved regions were identified in the deduced protein subunits, which were also used in a phylogenetic analysis to explore their evolutionary relationship with homologue sequences of other insect species. Analyses of the UCR of each hexamerin gene revealed a total of six overrepresented DNA motifs, indicating co-regulation. One of these motifs is a potential binding site for the protein Usp, and suggested gene regulation by JH. This hypothesis was reinforced by manipulating JH-levels in experiments in vivo, which resulted in JH-induction of the expression of hexamerin genes in larval fat body. Apparently under a high dietary protein input as occurring during larval stage, JH induces hexamerins for a high expression.

The detailed expression studies using fat body, ovaries and testes revealed that: (1) the four hexamerin genes are highly transcribed in the larval fat body, and are likely involved in hexamerin synthesis for amino acid storage and use during pupal stage; (2) in young adult workers, the expression of *hex 70a *in the fat body is in accordance with the idea of amino acid storage for a later support of foraging; (3) the expression of hexamerin genes in larval and pupal gonads suggests a role in ovaries and testes differentiation, and in spermatogenesis; (4) the expression of *hex 110 *in the ovaries of egg-laying queens, was associated with ovary activity for egg production; (5) at definite points of the honey bee development, the inverse relationship between the fat body levels of hexamerin transcripts and JH titer suggests that hexamerins regulate JH availability and, consequently, may be involved in the processes of caste-differentiation and worker transition to foraging.

Together, the findings of the present study are significant in that they highlighted the potential participation of hexamerins in important aspects of the life cycle of a social insect, in addition to their primordial role in metamorphosis.

## Methods

### Honey bees

Africanized honey bees were collected from hives maintained at the apiary of the University of São Paulo in Ribeirão Preto, Brazil. Developing queens (reared by standard apicultural procedures), workers and drones were staged according to Rembold et al [65], Michelette and Soares [66], and Tozetto et al [67], respectively. Adult workers and drones of known ages were obtained by paint marking the newly emerged ones and returning them to their hives to be collected a specified number of days later. Adult virgin queens were used at the third day after emergence, and egg-laying queens of unknown ages were collected from colonies kept in our apiary.

### Fat body and gonad samples used in hexamerin expression studies

Transcripts for the four hexamerin genes were assessed in the fat body of workers, queens and drones collected at different ontogenetic stages (larval, pupal and adult). Larvae were collected at the 4^th ^and 5^th ^instars, and individually sampled for total RNA extraction. As at the 4^th ^instar the fat body removed by dissection frequently resulted in small quantities of total RNA, we opted to use the whole larvae as fat body source. To optimize comparisons within larval stage, the same was done for the 5^th ^instar larvae. The abdominal carcass (dorsal integument and subjacent fat body) from pupae, pharate-adults and adults was sufficient to obtain individual RNA amounts, and then it was used as fat body source. Expression was also investigated in the ovaries of queens and workers, and in the testes of drones, during pre-imaginal and adult stages. Ovaries and testes were carefully dissected and exhaustively washed in Ringer saline to eliminate contaminant fat body before being used for RNA extractions. Data from our laboratory concerning *hex 110 *[20], *hex 70b *[19] and *hex 70a *[21] gene expression in the worker fat body, and concerning that of *hex 70a *[21] in female and male gonads, were used for comparisons.

### Testing the effect of JH on the expression of hexamerin genes

To test the effect of JH on the expression of hexamerin genes, a commercial JH III (Fluka) was diluted in acetone to make 10 μg per μl, and 1 μl was topically applied on 5^th ^instar worker larvae at the feeding phase (L5F). Control larvae at the same age were topically treated with 1 μl acetone. To obtain age-controlled worker larvae, the queen was caged on a comb and left to lay eggs for 6 h. When age-synchronized larvae reached the L5F stage, the comb was retrieved from the hive and transported to the laboratory for hormone treatment. The hormone (or acetone only) was carefully deposited on each larva in its comb cell using a micropipette. In this occasion, the comb was mapped for further identification of treated and control larvae, and thereafter they were returned to the hive. Treated and control larvae were collected after 24 h for RT-PCR analyses.

### Characterization of hexamerin coding sequences (CDSs)

The previously described N-terminal sequences of two of the honey bee hexamerins, HEX 70c (AYYAGRHTADMFFLH) and HEX 110 (APNVKQRAADQDLLNKQQDVIQLLQKISQPIPNQELQNLG) [23], were individually aligned against the Official Gene Set database [68,69]. Matching predicted amino acid sequences (GB13613-PA and GB14361-PA) were identified, and the corresponding nucleotide sequences (GB13613-RA and GB14361-RA) were annotated in the Artemis 7.0 platform [22] and used to design primers for experimental determination of the complete *hex 70c *and *hex 110 *CDSs (a short *hex 110 *cDNA fragment of 180 bp had been previously cloned and sequenced by our research group, [20]). The primers [*hex 70c *(PIR and 3F; 2R and PIF), and *hex 110 *(5R and 2F; 2R and 0F)] (see Additional file 13) were combined for PCR amplification from first-strand cDNAs obtained by reverse transcription (see RT-PCR analysis below) of total RNA from 5th instar worker larvae.

Amplicons were purified and subcloned using the TOPO TA-cloning kit (Invitrogen). Insert-containing plasmids were subjected to sequencing reactions using the primers described in Additional file 1 and M13-forward and reverse universal primers. Dideoxy sequencing was performed in an automatic sequencer (ABI Prism 310, Applied Biosystems) using BigDye Terminator v3.0 Cycle Sequencing Reaction (Applied Biosystems). Sequences were analyzed using Sequencher (version 4.7, Gene Codes Corporation), Artemis software and BLAST algorithms.

For purposes of data comparison, we used the *hex 70b *CDS, and the *hex 70a *CDS plus part of its 5' and 3' UTRs previously cloned and sequenced by our research group ([19,21]; see Additional file 14 for the accession numbers).

### Characterization of potential regulatory sequences in UCRs

A pipeline for motif discovery was designed based on reliable strategies previously proposed by MacIsaac et al [70], and adapted to analyze the honey bee genome [71,72]. This pipeline integrates three motif-detection programs: AlignAce [73], MEME [74] and MDscan [75]. Honey bee intergenic databases were constructed for 1.5, 3 and 6 kb sequence sizes that were trimmed whenever another open reading frame (ORF) was found to be flanking these regions. These databases were exploited for score calculations using group specificity scores (Church scores) [76], ROC-AUC scores [77] and Enrichment scores [78]. Two additional specific score metrics, the MAP score from AlignAce and MDscan and the E-value from MEME, were also used as a first filter for selecting the most significant motifs (MAP > 5 and E-value ≤ 1e-05). The second filter was set up to decrease the amount of spurious hits among the identified DNA motifs (Church ≤ 1e-04, ROC-AUC ≥ 0.7 and P-value for enrichment ≤ 1e-04). The main criterion for identifying known regulatory sites among the six overrepresented motifs was the alignment of the PSSM (Position-Specific Scoring Matrix) for each hexamerin motif with the D. melanogaster sites as described in the TRANSFAC database, version 2008.2 [79]. Only the alignments passing a threshold of 80% identity for each PSSM were considered as significant matches. The correlation among hexamerin transcription profiles in developing queens, workers and drones, and the occurrence of DNA motifs in hexamerin gene UCRs were represented as networks based on concepts from graph theory [80] and complex networks [81,82].

### Molecular phylogenetic analysis

Hexamerins and hemocyanins were searched for in public databases of protein sequences (Additional file 14) by using HMMER [83] for identifying the hemocyanin C (PF03723.5), N (PF03722.5) and M (PF00372.10) domains as described in the Pfam database [84]. A multiple alignment was performed using Muscle [85] with default parameters (Additional file 15). The phylogenetic tree was reconstructed by bayesian inference (MrBayes v3.1.2) using the Blosum model and gamma distribution of substitution rates. Metropolis-coupled Markov chain Monte Carlo sampling was performed (with one cold and three heated chains) setting the number of generations to 300,000 and trees were sampled every 100^th ^generations. The average standard deviation of split frequency was 0.005 after 300,000 generations. The posterior probabilities were estimated by discarding the first 30% samples.

### RT-PCR analysis

The expression of hexamerin genes was evaluated in the fat body and gonads of developing queens, workers and drones by semi-quantitative RT-PCR using specific primers (*hex 110*: 2F and 2R; *hex 70b*: RT-PCR-F and RT-PCR-R; *hex 70c*: JUF and JUR, see Additional file 13) to generate 659, 456 and 171 bp cDNA fragments, respectively. Total RNA was extracted from fat body, ovaries and testes using Trizol reagent (Invitrogen). The RNA concentration of each extracted sample was measured using a GeneQuant spectrophotometer (Pharmacia). Purity was determined by the 260/280 nm ratio considering values between 1.8 and 2. RNA integrity was verified using denaturing agarose gel (1.2%) electrophoresis and ethidium bromide staining. RNA samples were incubated at 37°C in the presence of 3 units of RNase-free DNase (Promega) for 40 min to eliminate contaminant DNA, followed by 15 min at 70°C to inactivate the enzyme. First-strand cDNA was synthesized by reverse transcription using 2.5 μg of total RNA, SuperScript II reverse transcriptase and an oligo dT(12-18) primer (Invitrogen). Negative control reactions without the enzyme were also prepared in parallel. After establishing the adequate number of cycles to avoid saturation, aliquots of cDNAs diluted 1:5 (v/v) in water were subjected to PCR (27 cycles of 30 s at 94°C, 1 min at 58°C and 1 min at 72°C). The amplified products were analyzed by electrophoresis in 1.2% agarose gels containing ethidium bromide. An *A. mellifera actin *gene (GenBank accession number AB023025), which is constitutively expressed during development [86], was used to control for cDNA loading. The primers used for *actin *gene amplification were ACT - F and ACT - R (Additional file 13), and the thermal cycling program was the same as described above. Primer pairs used in RT-PCR analysis (as well as those specified in Additional file 13) were designed to span at least one intron. Therefore, possible contamination by genomic DNA in RT-PCRs could be easily identified by the detection of a distinct, larger, band following electrophoresis in ethidium bromide-stained agarose gels.

### Real-time RT-PCR analysis

In order to quantitatively compare the levels of hexamerin transcripts in queens, workers and drones during the larval-pupal transition and in adults, we used the ΔΔC_T _method where the relative amount of transcripts is given by 2^-ΔΔC^_T _(Applied Biosystems User bulletin #2; [87]). We previously performed validation experiments to verify the efficiencies of amplification of the targets and the endogenous reference. The *rp 49 *gene [GenBank:AF441189], which is expressed in similar levels during honey bee development [86] was used as the endogenous reference. Amplification of *rp 49 *was done with the primers R and F (Additional file 13). Specific primers were used to amplify hexamerin genes (*hex 70a*: RTR and RTF; *hex 70c*: 2F and 1R; *hex 110*: 3R and 4F; *hex 70b*: RTR and RTF; Additional file 13). Using serial cDNA dilutions, the efficiency (E) of the reactions was calculated (E = 10^(-1/slope)^) for each gene and showed to be approximately equal. Amplifications were conducted in a 7500 Real Time PCR System (Applied Biosystems) using 20 μL reaction volumes containing 10 μL SYBR Green Master Mix 2× (Applied Biosystems), 1 μL first-strand cDNA (0.25 μg/μL, prepared from total RNA extracted from the fat body as described above for RT-PCR analysis), 7.4 μL water and 1 pmol of each specific primer. Reactions not including the SuperScript II reverse transcriptase (Invitrogen), or cDNA template, were prepared as negative controls. We used an initial cycle of 50°C for 2 min, a denaturation step of 95°C for 10 min, followed by a two-step cycling condition (40 cycles of 95°C for 15 s, and 60°C for 1 min). Each run was followed by a melting curve analysis to confirm the specificity of amplification and absence of primer dimers. To check reproducibility, each SYBR green assay was done in duplicate and repeated with three independent samples. Baseline and threshold were correctly set to obtain accurate C_T _values, which were exported into an MS Excel spreadsheet (Microsoft Inc.) for 2^-ΔΔC^_T _calculations.

Pearson's Correlation Coefficient R was used to verify a possible association among the expression profiles of the four honey bee hexamerin genes in queens, workers and drones during the larval-pupal transition. The statistical significance of the R values was evaluated using a t-test, with R > 0.81 indicating 95% confidence that the variables are truly correlated.

## Abbreviations

cDNA: complementary DNA; RT-PCR: reverse transcription polymerase chain reaction; UCR: upstream control region; CDS: coding sequence; UTR: untranslated region; *hex*, hexamerin gene; HEX: hexamerin subunit; JH: juvenile hormone; DNAse: deoxyribonuclease; Glx: glutamine/glutamic acid; Usp: ultraspiracle.

## Authors' contributions

JRM conducted the bioinformatic analyses, directed laboratory activities, undertook statistical analyses, interpretation of results and assisted with preparation of this manuscript. FMFN assisted with primers design, bioinformatic analyses, and manuscript edits. ASC conducted the bioinformatic and phylogenetic analyses and authored the corresponding Method and Result sections. ZLPS assisted with project design and development. MMGB conducted project design with considerable input into direction of research, interpretation of results and preparation of this manuscript. All authors read and approved the final manuscript.

## Supplementary Material

Additional file 1 **Complete coding sequence of the *hex110 *gene. Exons are separated by asterisks. Primer sequences used for expression studies and gene sequencing are underlined**. *hex110 *complete coding sequence. Click here for file

Additional file 2 **Complete coding sequence of the *hex70a *gene. Exons are separated by asterisks. Primer sequences used for expression studies and gene sequencing are underlined. Dashed lines indicate 5' and 3' UTR regions**. *hex70a *complete coding sequence. Click here for file

Additional file 3 **Complete coding sequence of the *hex70b *gene. Exons are separated by asterisks. Primer sequences used for expression studies and gene sequencing are underlined**. *hex70b *complete coding sequence. Click here for file

Additional file 4 **Complete coding sequence of the *hex70c *gene. Exons are separated by asterisks. Primer sequences used for expression studies and gene sequencing are underlined. Dashed lines indicate 5' and 3' UTR regions**. *hex70c *complete coding sequence. Click here for file

Additional file 5 **HEX110 deduced amino acid sequence. Signal peptide is indicated by a dotted line, and a dashed line shows Danty's hexamerin motif **[23]. **Asterisks indicate glicosylation sites. The conserved histidine is double underlined**. HEX110 deduced amino acid sequence. Click here for file

Additional file 6 **HEX70a deduced amino acid sequence. Signal peptide is indicated by a dotted line, and a dashed line shows Danty's hexamerin motif **[23]. **Asterisks indicate glicosylation sites. The conserved histidine is double underlined. LSP signature-1 motif is indicated (+++). LSP signature-2 motif is in bold. Protease cleavage site is underlined. The exopterygote and endopterygote conserved glycine-14 is indicated (#)**. HEX70a deduced amino acid sequence. Click here for file

Additional file 7 **HEX70b deduced amino acid sequence. Signal peptide is indicated by a dotted line, and a dashed line shows Danty's hexamerin motif **[23]. **Asterisks indicate glicosylation sites. The conserved histidine is double underlined. LSP signature-1 motif is indicated (+++). LSP signature-2 motif is in bold. Protease cleavage site is underlined. The exopterygote and endopterygote conserved glycine-14 is indicated (#)**. HEX70b deduced amino acid sequence. Click here for file

Additional file 8 **HEX70c deduced amino acid sequence. Signal peptide is indicated by a dotted line, and a dashed line shows Danty's hexamerin motif **[23]. **Asterisks indicate glicosylation sites. The conserved histidine is double underlined. LSP signature-1 motif is indicated (+++). LSP signature-2 motif is in bold. The exopterygote and endopterygote conserved glycine-14 is indicated (#)**. HEX70c deduced amino acid sequence. Click here for file

Additional file 9 **Hydropathy profiles produced for HEX70a, HEX70b, HEX70c and HEX110 sequences using Phobius http://phobius.sbc.su.se**. Hydropathy profiles of the deduced hexamerin sequences of the honey bee. Click here for file

Additional file 10 **Multiple alignment including HEX 70a, HEX 70b, HEX 70c and HEX110 using ClustalW 1.83**. Multiple alignment of the deduced hexamerin sequences of the honey bee. Click here for file

Additional file 11 **Expression of hexamerin genes in the fat body of developing and adult workers, queens and drones. The abundance of *hex110*, *hex70a*, *hex70b *and *hex70c *transcripts as detected by semi-quantitative RT-PCR followed by electrophoresis of the amplified cDNA on ethidium bromide-stained agarose gel using an *A. mellifera *actin as a loading control. L5F and L5S: feeding and spinning phases of the 5th larval instar. PP: pharate pupae. Pw, Pp, Pdp, Pb, Pbl, Pbm and Pbd: successive phases of the worker and queen pupal stage. P1 to P13: successive phases of the drone pupal stage. NE: newly-emerged adults. Numbers indicate adult age in days**. Expression of hexamerin genes in the fat body of developing and adult workers, queens and drones. Click here for file

Additional file 12 **Expression of hexamerin genes in the gonads of developing and adult workers, queens and drones. The abundance of *hex110*, *hex70a*, *hex70b *and *hex70c *transcripts as detected by semi-quantitative RT-PCR followed by electrophoresis of the amplified cDNA on ethidium bromide-stained agarose gel using an *A. mellifera *actin as a housekeeping gene. L5F and L5S: feeding and spinning phases of the 5th larval instar. PP: pharate pupae. Pw, Pp, Pb, Pbl, Pbm and Pbd: successive phases of the worker and queen pupal stage. P1 to P13: successive phases of the drone pupal stage. NE: newly-emerged adults. EL: egg-laying queens. Numbers indicate adult age in days**. Expression of hexamerin genes in the gonads of developing and adult workers, queens and drones. Click here for file

Additional file 13 **Specific primers used for sequencing and expression analyses**. as title. Click here for file

Additional file 14 **Accession numbers and references concerning to hexamerins and hemocyanins included in molecular phylogeny analysis**. as title. Click here for file

Additional file 15 **Multiple alignment used for phylogenetic tree reconstruction**. as title. Click here for file
