# Supplementary figures and images for: The four hexamerin genes in the honey bee: structure, molecular evolution and function deduced from expression patterns in queens, workers and drones
Source: BMC Mol Biol. 2010 Mar 26;11:23. doi: 10.1186/1471-2199-11-23 (PMC2861669; doi:10.1186/1471-2199-11-23)

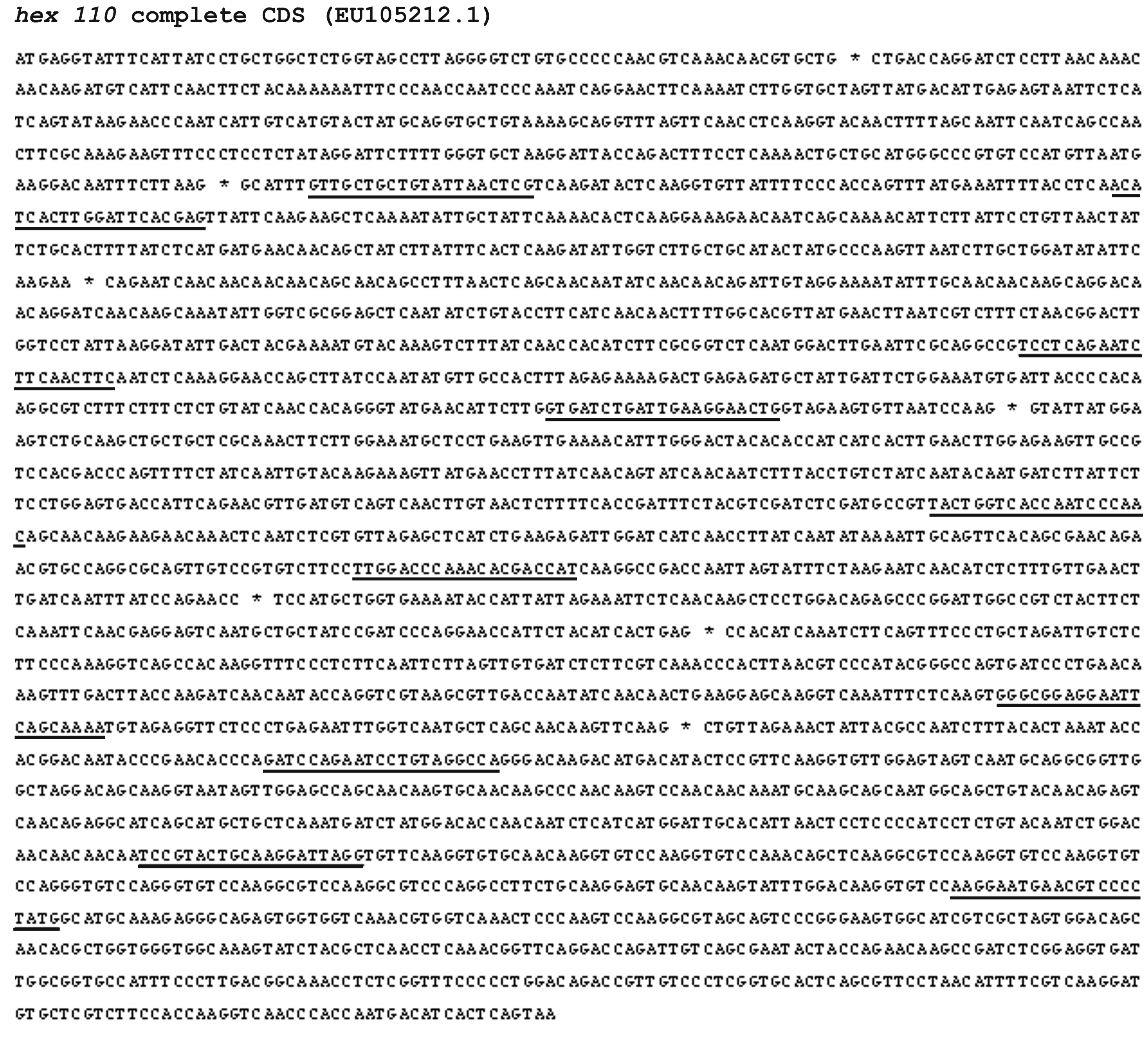

Supplement: Additional file 1 — Complete coding sequence of the hex110 gene. Exons are separated by asterisks. Primer sequences used for expression studies and gene sequencing are underlined. hex110 complete coding sequence. [file 1471-2199-11-23-S1.TIFF]

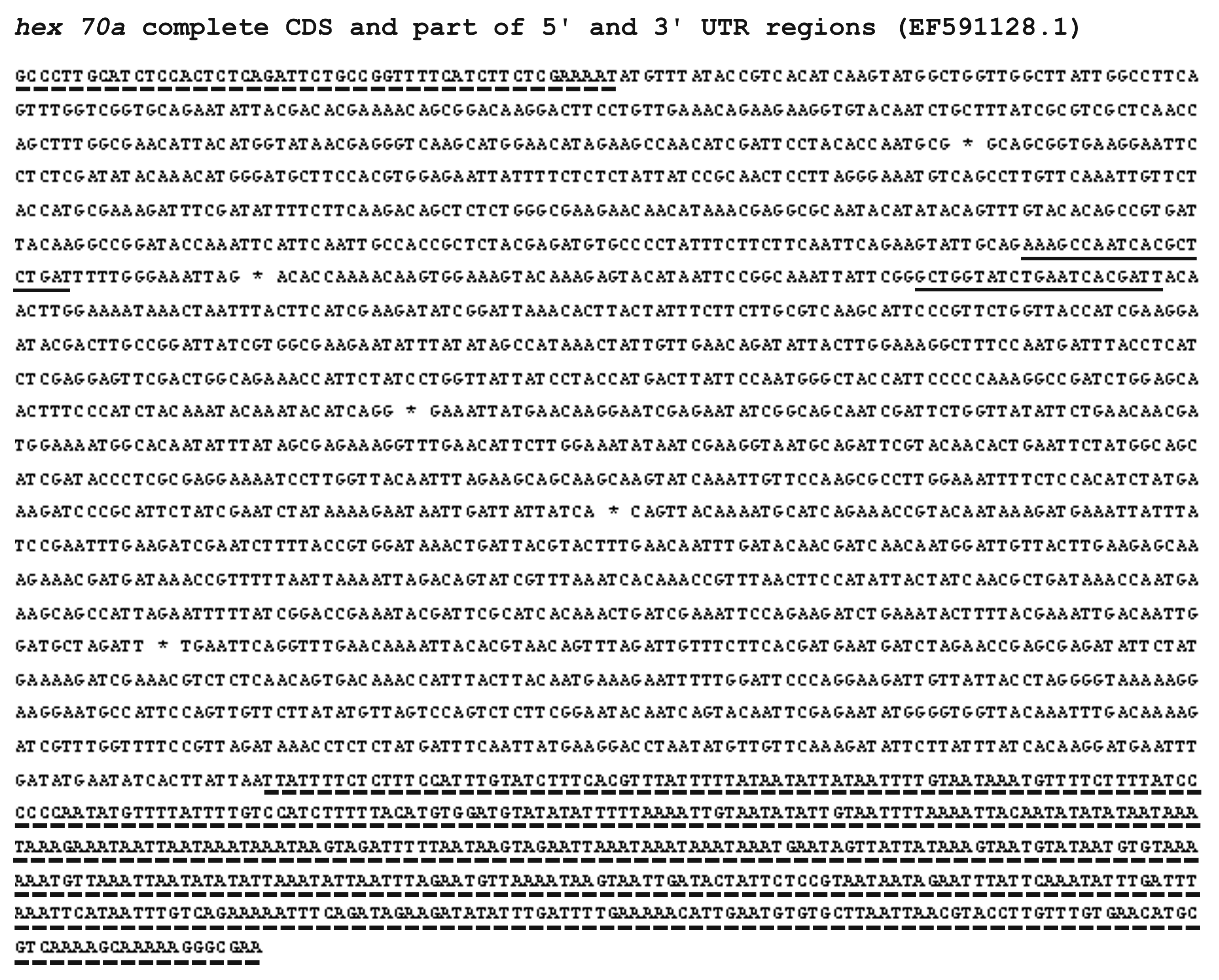

Supplement: Additional file 2 — Complete coding sequence of the hex70a gene. Exons are separated by asterisks. Primer sequences used for expression studies and gene sequencing are underlined. Dashed lines indicate 5' and 3' UTR regions. hex70a complete coding sequence. [file 1471-2199-11-23-S2.TIFF]

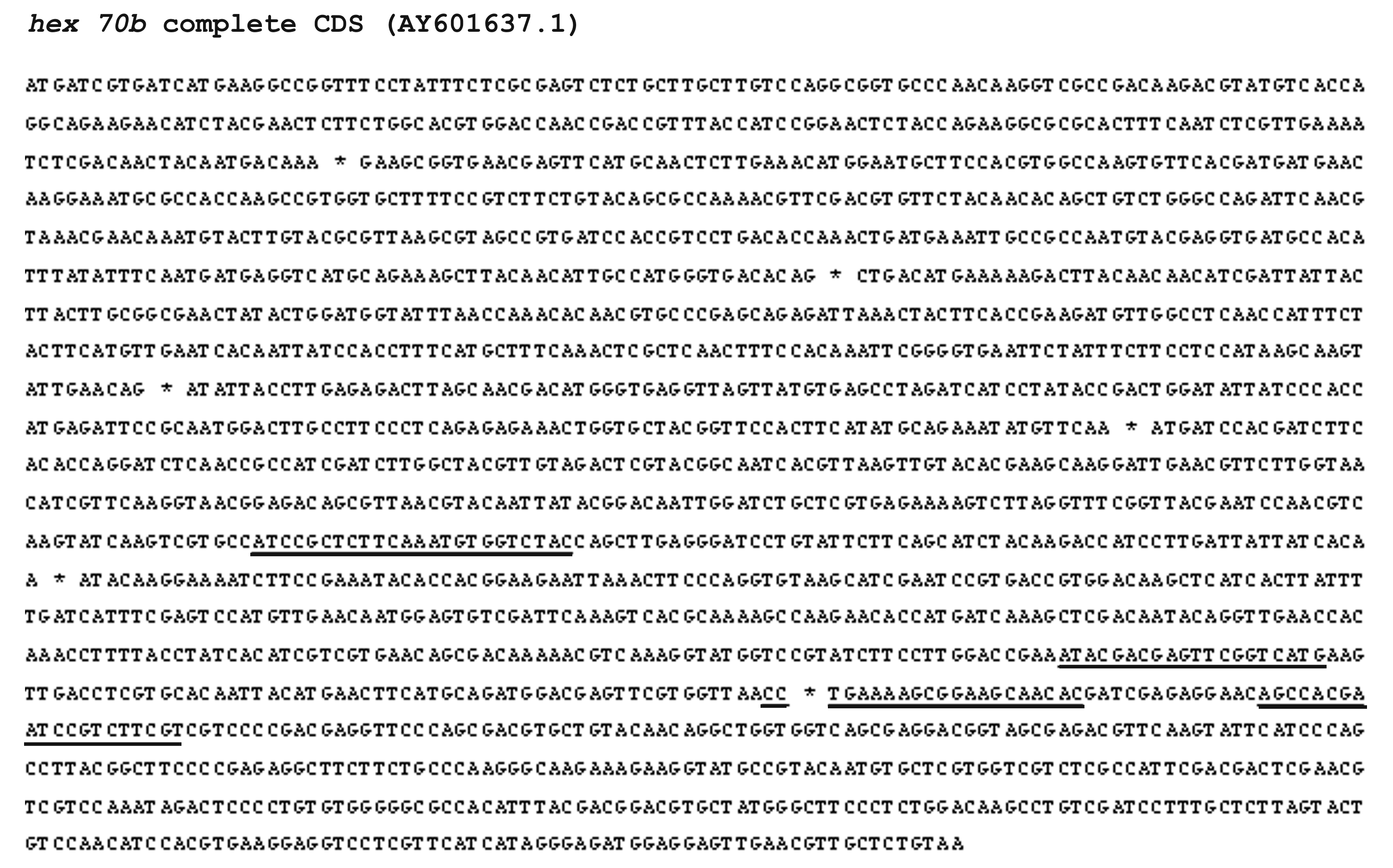

Supplement: Additional file 3 — Complete coding sequence of the hex70b gene. Exons are separated by asterisks. Primer sequences used for expression studies and gene sequencing are underlined. hex70b complete coding sequence. [file 1471-2199-11-23-S3.TIFF]

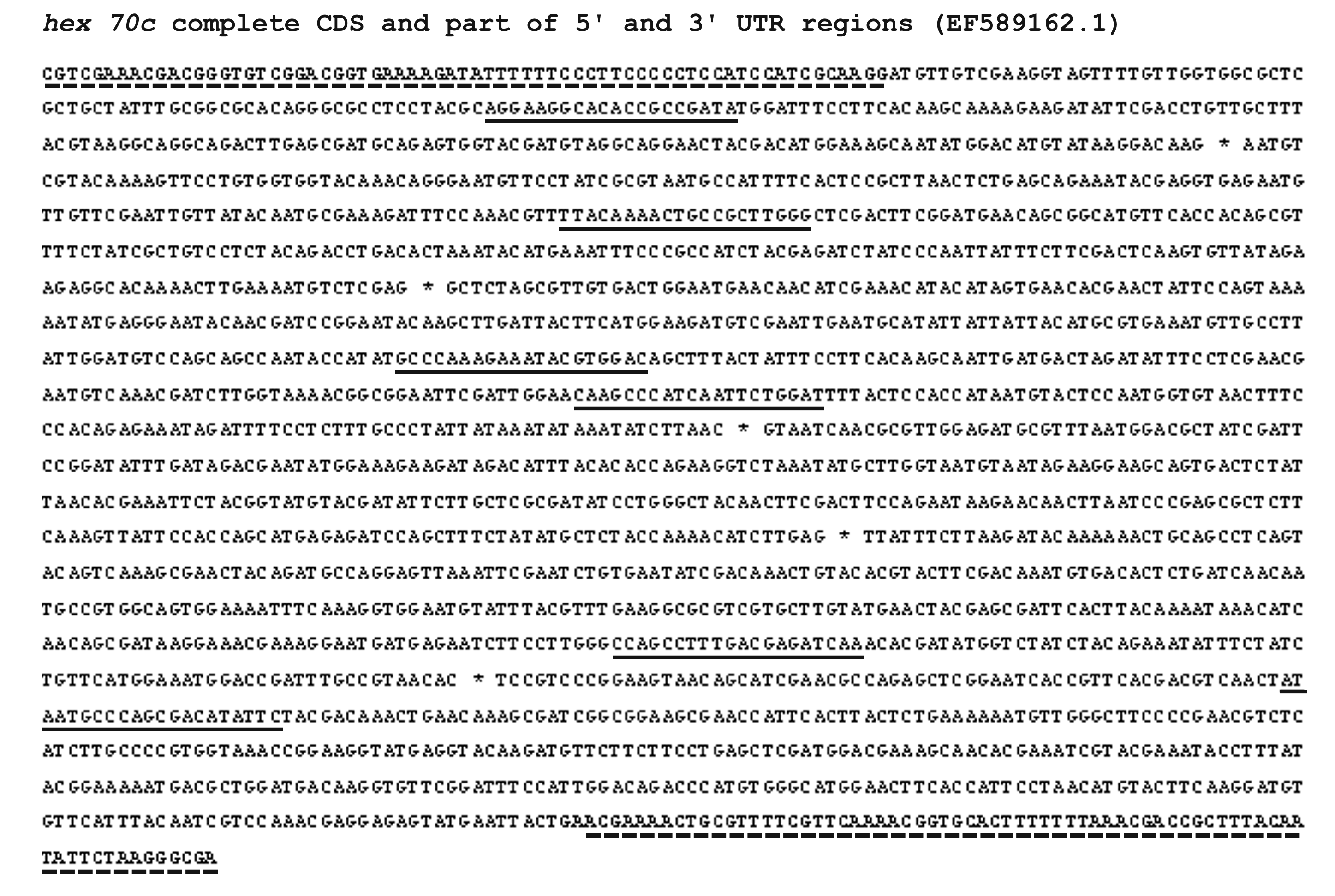

Supplement: Additional file 4 — Complete coding sequence of the hex70c gene. Exons are separated by asterisks. Primer sequences used for expression studies and gene sequencing are underlined. Dashed lines indicate 5' and 3' UTR regions. hex70c complete coding sequence. [file 1471-2199-11-23-S4.TIFF]

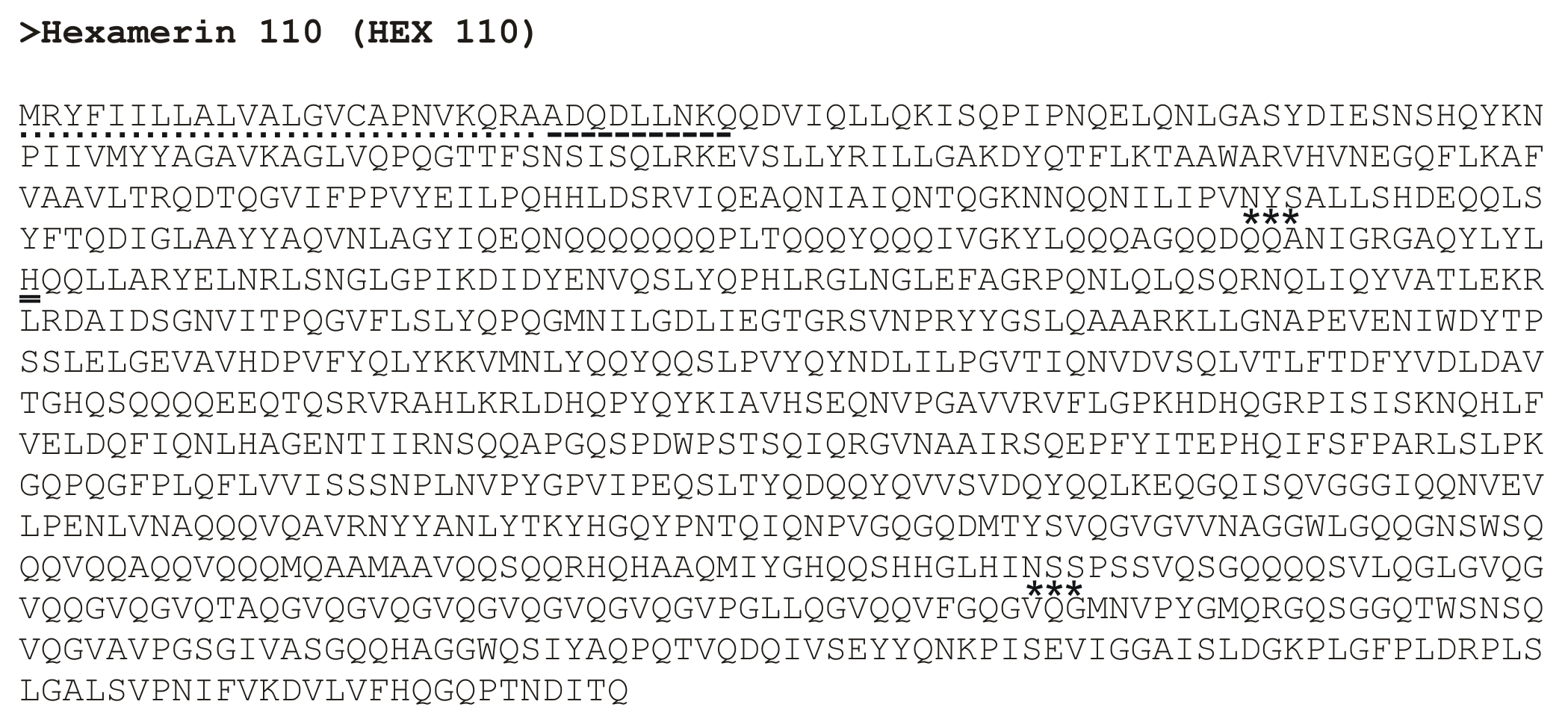

Supplement: Additional file 5 — HEX110 deduced amino acid sequence. Signal peptide is indicated by a dotted line, and a dashed line shows Danty's hexamerin motif [23]. Asterisks indicate glicosylation sites. The conserved histidine is double underlined. HEX110 deduced amino acid sequence. [file 1471-2199-11-23-S5.TIFF]

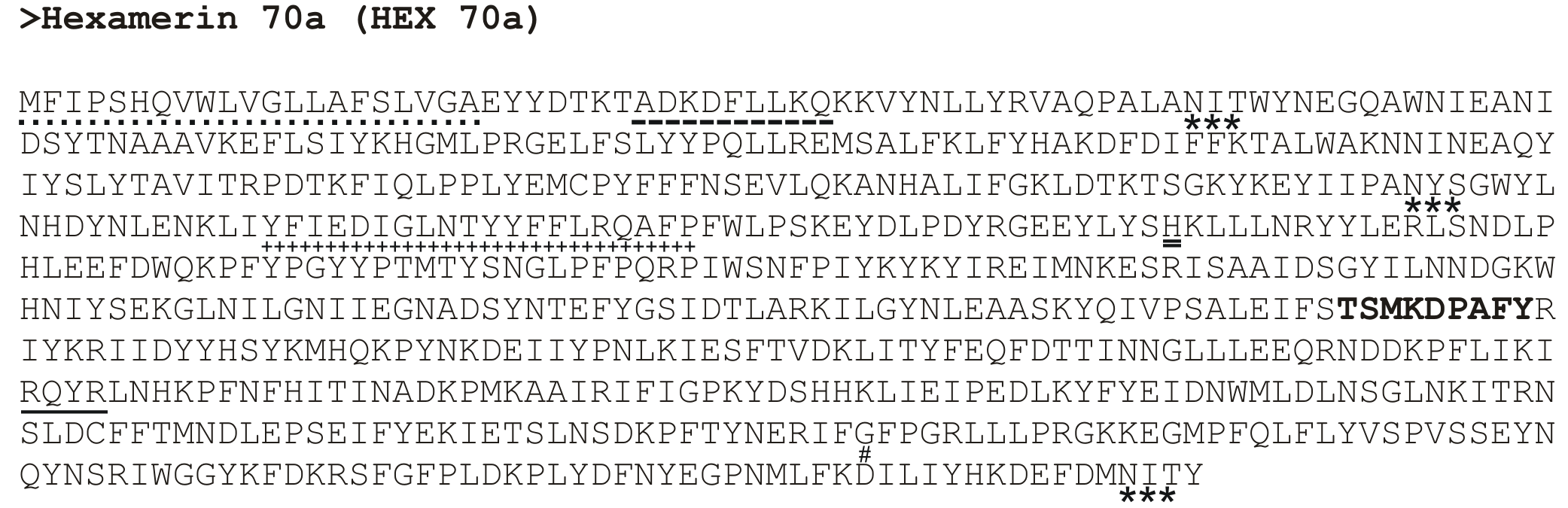

Supplement: Additional file 6 — HEX70a deduced amino acid sequence. Signal peptide is indicated by a dotted line, and a dashed line shows Danty's hexamerin motif [23]. Asterisks indicate glicosylation sites. The conserved histidine is double underlined. LSP signature-1 motif is indicated (+++). LSP signature-2 motif is in bold. Protease cleavage site is underlined. The exopterygote and endopterygote conserved glycine-14 is indicated (#). HEX70a deduced amino acid sequence. [file 1471-2199-11-23-S6.TIFF]

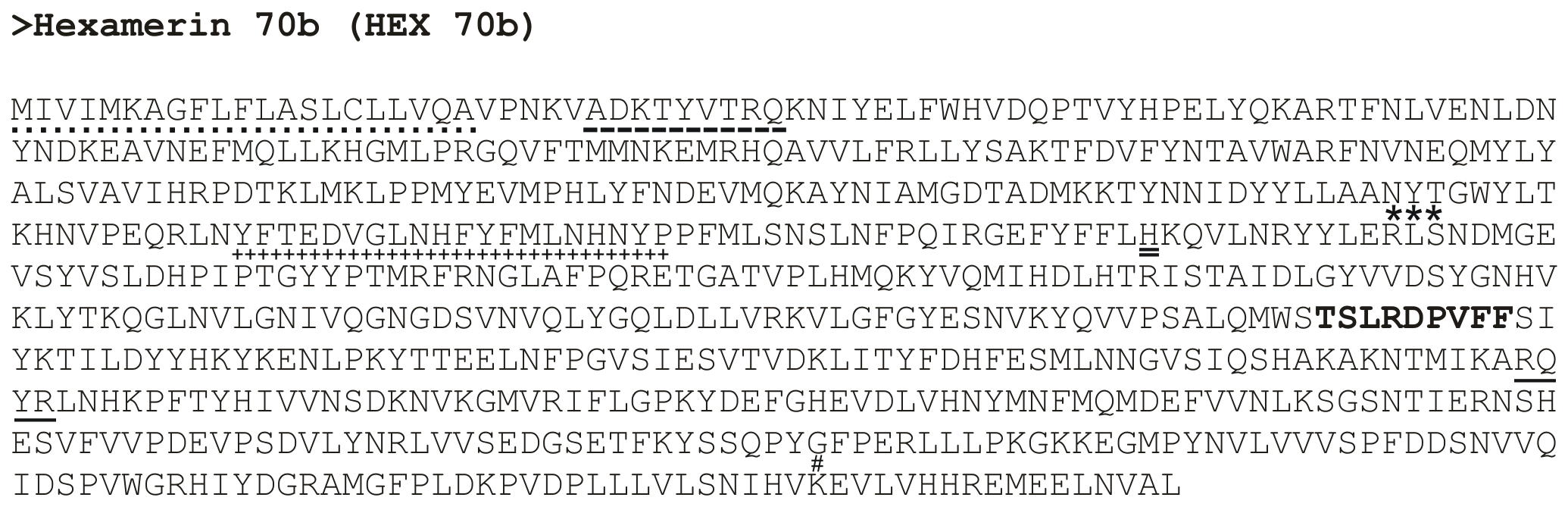

Supplement: Additional file 7 — HEX70b deduced amino acid sequence. Signal peptide is indicated by a dotted line, and a dashed line shows Danty's hexamerin motif [23]. Asterisks indicate glicosylation sites. The conserved histidine is double underlined. LSP signature-1 motif is indicated (+++). LSP signature-2 motif is in bold. Protease cleavage site is underlined. The exopterygote and endopterygote conserved glycine-14 is indicated (#). HEX70b deduced amino acid sequence. [file 1471-2199-11-23-S7.TIFF]

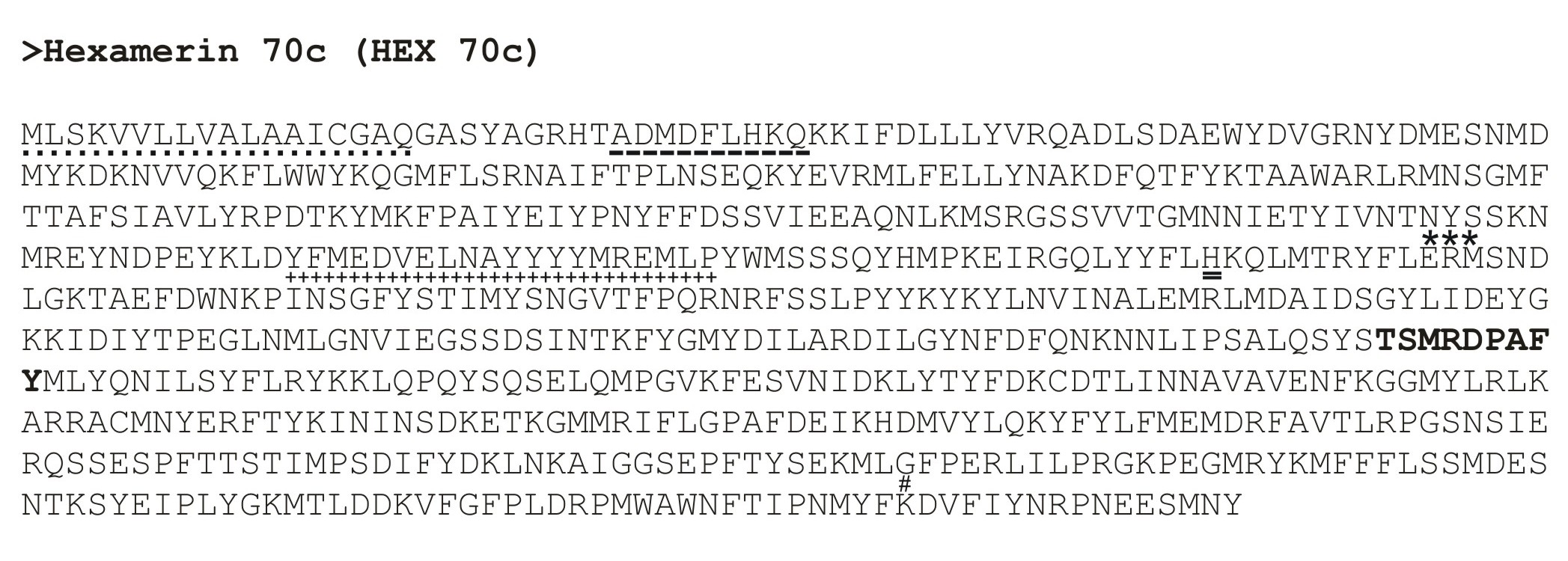

Supplement: Additional file 8 — HEX70c deduced amino acid sequence. Signal peptide is indicated by a dotted line, and a dashed line shows Danty's hexamerin motif [23]. Asterisks indicate glicosylation sites. The conserved histidine is double underlined. LSP signature-1 motif is indicated (+++). LSP signature-2 motif is in bold. The exopterygote and endopterygote conserved glycine-14 is indicated (#). HEX70c deduced amino acid sequence. [file 1471-2199-11-23-S8.JPEG]

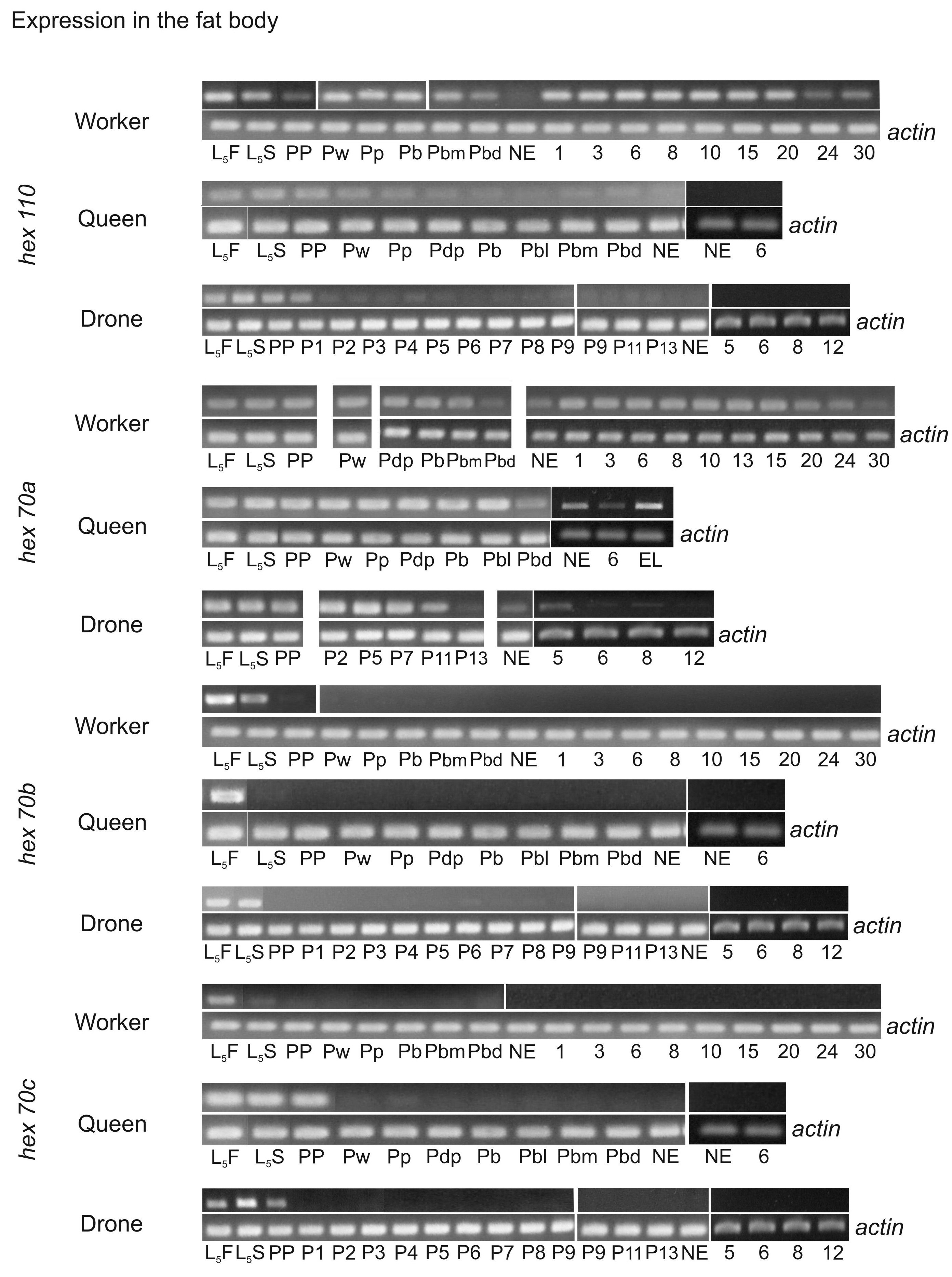

Supplement: Additional file 11 — Expression of hexamerin genes in the fat body of developing and adult workers, queens and drones. The abundance of hex110, hex70a, hex70b and hex70c transcripts as detected by semi-quantitative RT-PCR followed by electrophoresis of the amplified cDNA on ethidium bromide-stained agarose gel using an A. mellifera actin as a loading control. L5F and L5S: feeding and spinning phases of the 5th larval instar. PP: pharate pupae. Pw, Pp, Pdp, Pb, Pbl, Pbm and Pbd: successive phases of the worker and queen pupal stage. P1 to P13: successive phases of the drone pupal stage. NE: newly-emerged adults. Numbers indicate adult age in days. Expression of hexamerin genes in the fat body of developing and adult workers, queens and drones. [file 1471-2199-11-23-S11.JPEG]

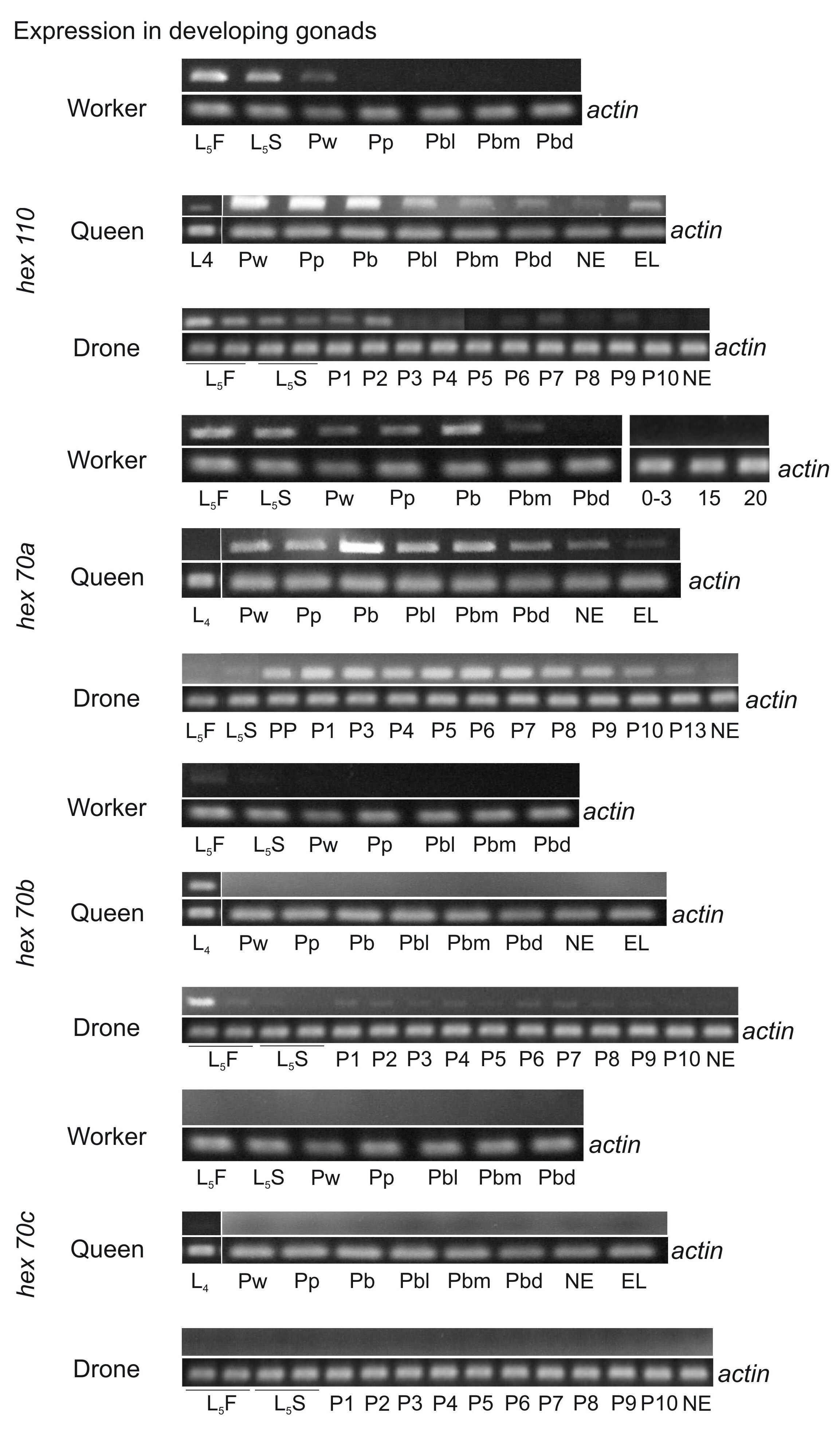

Supplement: Additional file 12 — Expression of hexamerin genes in the gonads of developing and adult workers, queens and drones. The abundance of hex110, hex70a, hex70b and hex70c transcripts as detected by semi-quantitative RT-PCR followed by electrophoresis of the amplified cDNA on ethidium bromide-stained agarose gel using an A. mellifera actin as a housekeeping gene. L5F and L5S: feeding and spinning phases of the 5th larval instar. PP: pharate pupae. Pw, Pp, Pb, Pbl, Pbm and Pbd: successive phases of the worker and queen pupal stage. P1 to P13: successive phases of the drone pupal stage. NE: newly-emerged adults. EL: egg-laying queens. Numbers indicate adult age in days. Expression of hexamerin genes in the gonads of developing and adult workers, queens and drones. [file 1471-2199-11-23-S12.JPEG]
