# Supplementary material for: The four hexamerin genes in the honey bee: structure, molecular evolution and function deduced from expression patterns in queens, workers and drones
Source: BMC Mol Biol. 2010 Mar 26;11:23. doi: 10.1186/1471-2199-11-23 (PMC2861669; doi:10.1186/1471-2199-11-23)

Additional file 9: Hydropathy profile produced for AmHEX70a **(A)**, AmHEX70b **(B)**, AmHEX70c **(C)** and AmHEX110 **(D)** sequences using Phobius (http://phobius.sbc.su.se/)

(A) Hexamerin 70a:

Transmembrane region: 0
Signal-peptide: yes
Prediction: signal peptide with a h-region between position 8 and 16 that is cleaved between position 21 and 22

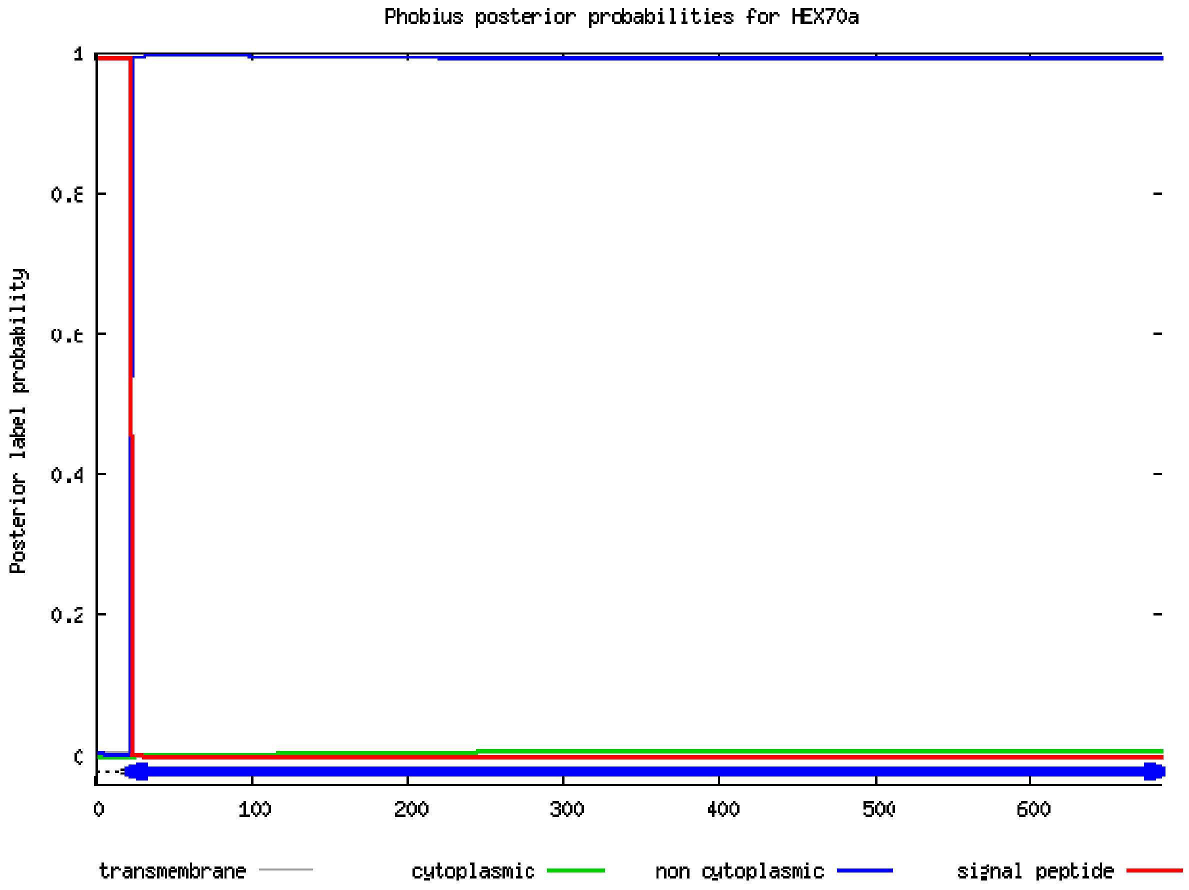

(B) Hexamerin 70b:

Transmembrane region: 0
Signal-peptide: yes
Prediction: signal peptide with a h-region between position 7 and 21 that is cleaved between position 21 and 22

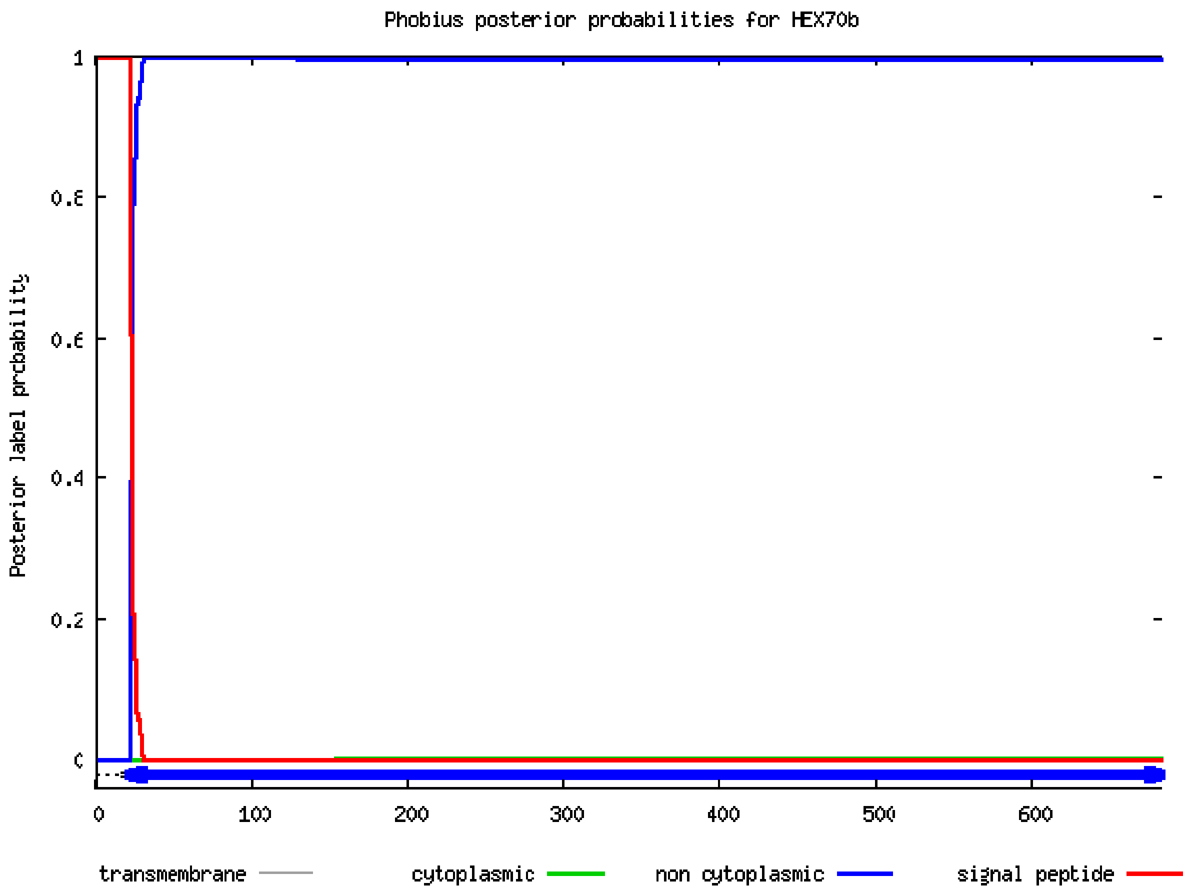

(C) Hexamerin 70c:

Transmembrane region: 0
Signal-peptide: yes
Prediction: signal peptide with a h-region between position 5 and 19 that is cleaved between position 19 and 20

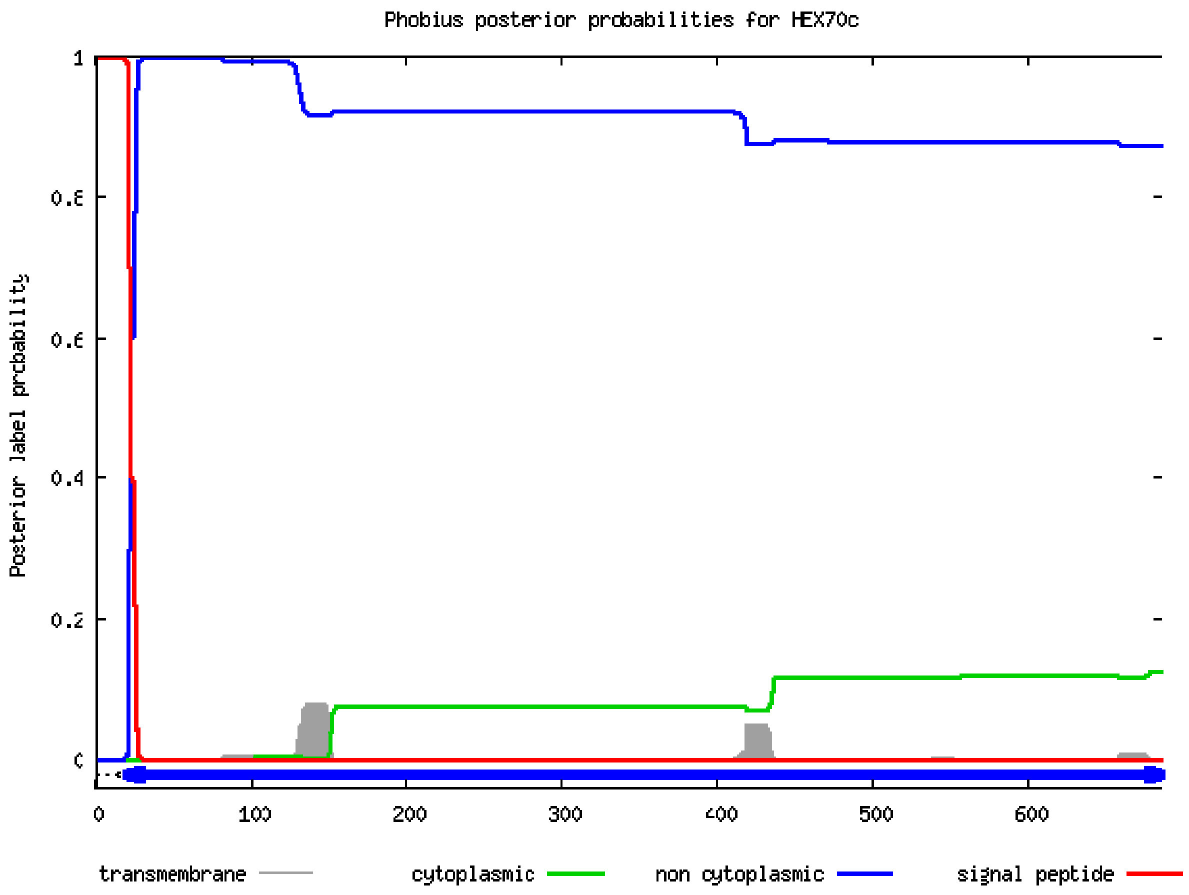

(D) Hexamerin 110:

Transmembrane region: 0
Signal-peptide: yes
Prediction: signal peptide with a h-region between position 4 and 16 that is cleaved between position 24 and 25

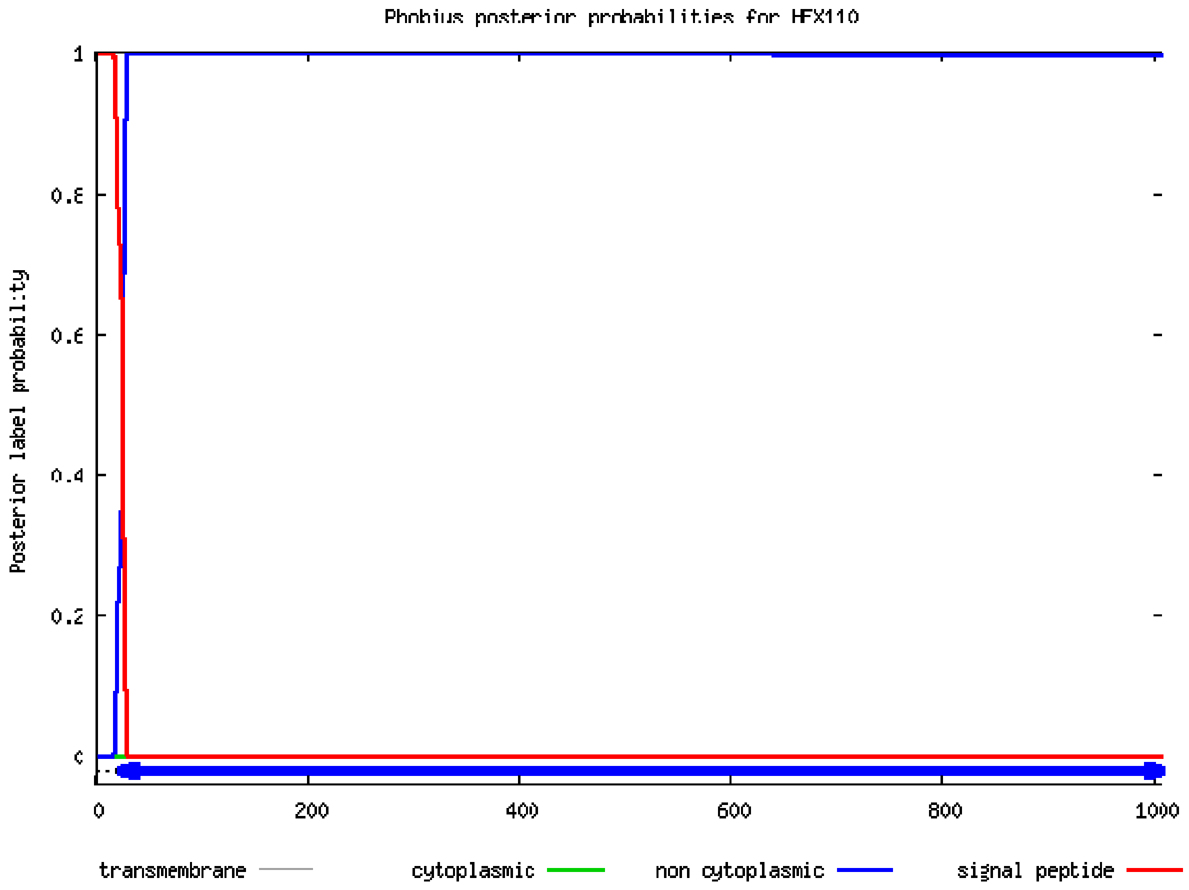

Supplement: Additional file 9 — Hydropathy profiles produced for HEX70a, HEX70b, HEX70c and HEX110 sequences using Phobius http://phobius.sbc.su.se. Hydropathy profiles of the deduced hexamerin sequences of the honey bee. [file 1471-2199-11-23-S9.PDF]
