# Supplementary material for: The four hexamerin genes in the honey bee: structure, molecular evolution and function deduced from expression patterns in queens, workers and drones
Source: BMC Mol Biol. 2010 Mar 26;11:23. doi: 10.1186/1471-2199-11-23 (PMC2861669; doi:10.1186/1471-2199-11-23)
Supplement: Additional file 10 — Multiple alignment including HEX 70a, HEX 70b, HEX 70c and HEX110 using ClustalW 1.83. Multiple alignment of the deduced hexamerin sequences of the honey bee. [file 1471-2199-11-23-S10.PDF]

Multiple alignment including HEX 70a, HEX 70b, HEX 70c and HEX110 using ClustalW 1.83

```
HEX70a MFIPSHQVWLVGLLAFS-LVGAEYYDTKTADKDFLLKQKKVYNLLYRVAQPALANITWYN
HEX70b MIV---IMKAGFLFLASLCLLVQAVPNKVADKTYVTRQKNIYELFWHVDQPTVYHPELYQ
HEX70c MLSK--VVLLVALAAICGAQGASYAGRHTADMDFLHKQKKIFDLLLYVRQADLSDAEWYD
HEX110 M-----RYFIILLALVALGVCAPNVKQRAADQDLLNKQQDVIQLLQKISQP-IPNQELQN
       *           *   .    .     :.**   : :*:.: :*:  : *. : .    :

HEX70a EGQAWNIEANIDSYTNAAAVKEFLSIYKHGMLP-RGELFSLYYPQLLREMSALFKLFYHA
HEX70b KARTFNLVENLDNYNDKEAVNEFMQLLKHGMLP-RGQVFTMMNKEMRHQAVVLFRLLYSA
HEX70c VGRNYDMESNMDMYKDKNVVQKFLWWYKQGMFLSRNAIFTPLNSEQKYEVRMLFELLYNA
HEX110 LGASYDIESNSHQYKNPIIVMYYAGAVKAGLVQPQGTTFSNSISQLRKEVSLLYRILLGA
        .  :::  * . *.:   *  :    * *:.  :.  *:    :   :   *:.::  *

HEX70a KDFDIFFKTALWAKNNINEAQYIYSLYTAVITRPDTKFIQLPPLYEMCPYFFFNSEVLQK
HEX70b KTFDVFYNTAVWARFNVNEQMYLYALSVAVIHRPDTKLMKLPPMYEVMPHLYFNDEVMQK
HEX70c KDFQTFYKTAAWARLRMNSGMFTTAFSIAVLYRPDTKYMKFPAIYEIYPNYFFDSSVIEE
HEX110 KDYQTFLKTAAWARVHVNEGQFLKAFVAAVLTRQDTQGVIFPPVYEILPQHHLDSRVIQE
       * :: * :** **: .:*.  :  ::  **: * **: : :*.:**: *  .::. *:::

HEX70a ANHALIFGKLDTKTSG-KYKEYIIPANYSGWYLNHDYNLENKLIYFIEDIGLNTYYFFLR
HEX70b AYNIAMGDTADMKKTYNNIDYYLLAANYTGWYLTKHNVPEQRLNYFTEDVGLNHFYFMLN
HEX70c AQNLKMSRGSSVVTGMNNIETYIVNTNYSSKNMREYNDPEYKLDYFMEDVELNAYYYYMR
HEX110 AQNIAIQN----TQGKNNQQNILIPVNYS----ALLSHDEQQLSYFTQDIGLAAYYAQVN
       * :  :           :  .  :: .**:          * :* ** :*: *  :*  :.

HEX70a QAFPFWLPSKEYDLP-----------------------------DYRGEEYLYSHKLLLN
HEX70b HNYPPFMLSNSLNFP-----------------------------QIRGEFYFFLHKQVLN
HEX70c EMLPYWMSSSQYHMPK----------------------------EIRGQLYYFLHKQLMT
HEX110 LAGYIQEQNQQQQQQPLTQQQYQQQIVGKYLQQQAGQQDQQANIGRGAQYLYLHQQLLA
               ... .                                 **  * : *: ::

HEX70a RYYLERLSNDLPHLEEFDWQKPFYPGYYPTMTYSNGLPFPQRPIWSNFPIYKYKYIREIM
HEX70b RYYLERLSNDMGEVSYVSLDHPIPTGYYPTMRFRNGLAFPQRETGATVPLHMQKYVQMIH
HEX70c RYFLERMSNDLGKTAEFDWNKPINSGFYSTIMYSNGVTFPQRNRFSSLPYYKYKYLNVIN
HEX110 RYELNRLSNGLGPIKDIDYEN-VQSLYQPHLRGLNGLEFAGRPQNLQLQSQRNQLIQYVA
       ** *:*:**.:     .. :: . . : . :   **: *. *     .     : :. :

HEX70a NKESRISAAIDSGYILNNDGKWHNIYSEKGLNILGNIIEGNADSYNTEFYGSIDTLARKI
HEX70b DLHTRISTAIDLGYVVDSYGNHVKLYTKQGLNVLGNIVQGNGDSVNVQLYGQLDLLVRKV
HEX70c ALEMRLMDAIDSGYLIDEYGKKIDIYTPEGLNMLGNVIEGSSDSINTKFYGMYDILARDI
HEX110 TLEKRLRDAIDSGNVITPQGVFLSLYQPQGMNILGDLIEGTGRSVNPRYYGSLQAAARKL
         . *:  *** * ::   *   .:*  :*:*:**::::*.. * * . **  :  .*.:

HEX70a LGYNLEAASKYQIVPSALEIFSTSMKDPAFYRIYKRIIDYYHSYKMHQKPYNKDEIIYPN
HEX70b LGFGYESNVKYQVVPSALQMWSTSLRDPVFFSIYKTILDYYHKYKENLPKYTTEELNFPG
HEX70c LGYNFDFQNKNNLIPSALQSYSTSMRDPAFYMLYQNILSYFLRYKKLQPQYSQSELQMPG
HEX110 LGNAPEVENIWDYTPSSLELGEVAVHDPVFYQLYKKVMNLYQQYQQSLPVYQYNDLILPG
       **   :     :  **:*:  ..:::**.*: :*: ::. :  *:     *  .::  *.

HEX70a LKIESFTVDKLITYFEQFDTTINNGLLLEEQRNDDK--PFLIKIRQYRLNHKPFNFHITI
HEX70b VSIESVTVDKLITYFDHFESMLNNGVSIQSHAKAKN---TMIKARQYRLNHKPFTYHIVV
HEX70c VKFESVNIDKLYTYFDKCDTLINNAVAVENFKGG-M--YLRLKARRACMNYERFTYKINI
HEX110 VTIQNVDVSQLVTLFTDFYVDLDAVTGHQSQQQQEEQTQSRVRAHLKRLDHQPYQYKIAV
       :.::.. :.:* * * .    ::     :.           :: :   :::: : ::* :
```

```
HEX70a NADKPMK-AAIRIFIGPKYDSH-HKLIEIPEDLKYFYEIDNWMLDLNSGLNKITRNSLDC
HEX70b NSDKNVK-GMVRIFLGPKYDEFGHEVDLVHNYMN-FMQMDEFVVNLKSGSNTIERNSHES
HEX70c NSDKETK-GMMRIFLGPAFDEIKHDMVYLQKYFYLFMEMDRFAVTLRPGSNSIERQSSES
HEX110 HSEQNVPGAVVRVFLGPKHDHQ-GRPISISKNQHLFVELDQFIQNLHAGENTIIRNSQQA
       ::::    . :*:*:** .*        : :    * ::*.:   *..* *.* *:* :.

HEX70a FFTMNDLEPSEIFYEKIETSLNSDKPFTYNE--RIFGFPGRLLLPRGKKEGMPFQLFLYV
HEX70b VFVVPDEVPSDVLYNRLVVSEDGSETFKYSS--QPYGFPERLLLPKGKKEGMPYNVLVVV
HEX70c PFTTSTIMPSDIFYDKLNKAIGGSEPFTYSE--KMLGFPERLILPRGKPEGMRYKMFFFL
HEX110 PGQSPDWPSTSQIQRGVNAAIRSQEPFYITEPHQIFSFPARLSLPKGQPQGFPLQFLVVI
               .:. :   :  :  ..:.*  ..  :  .** ** **:*: :*:  :.:. :

HEX70a SPVS-S------------------------------------------------------
HEX70b SPFDDS------------------------------------------------------
HEX70c SSMDES------------------------------------------------------
HEX110 SSSNPLNVPYGPVIPEQSLTYQDQQYQVVSVDQYQQLKEQGQISQVGGGIQQNVEVLPEN
       *. .

HEX70a ------------------------------------------------------------
HEX70b ------------------------------------------------------------
HEX70c ------------------------------------------------------------
HEX110 LVNAQQQVQAVRNYYANLYTKYHGQYPNTQIQNPVGQGQDMTYSVQGVGVVNAGGWLGQQ

HEX70a ------------------------------------------------------------
HEX70b ------------------------------------------------------------
HEX70c ------------------------------------------------------------
HEX110 GNSWSQQQVQQAQQVQQQMQAAMAAVQQSQQRHQHAAQMIYGHQQSHHGLHINSSPSSVQ

HEX70a ------------------------------------------------------------
HEX70b ------------------------------------------------------------
HEX70c ------------------------------------------------------------
HEX110 SGQQQQSVLQGLGVQGVQQGVQGVQTAQGVQGVQGVQGVQGVQGVQGVPGLLQGVQQVFG

HEX70a ------------------------------------------------------------
HEX70b ------------------------------------------------------------
HEX70c ------------------------------------------------------------
HEX110 QGVQGMNVPYGMQRGQSGGQTWSNSQVQGVAVPGSGIVASGQQHAGGWQSIYAQPQTVQD

HEX70a -------EYNQYNSRIWGGYKFDKRSFGFPLDKPLYDFNYEGPNMLFKDILIYHKDEFDM
HEX70b -------NVVQIDSPVWGRHIYDGRAMGFPLDKPVDPLLLVLSNIHVKEVLVHHREMEEL
HEX70c -------NTKSYEIPLYGKMTLDDKVFGFPLDRPMWAWNFTIPNMYFKDVFIYNRPNEES
HEX110 QIVSEYYQNKPISEVIGGAISLDGKPLGFPLDRPLSLGALSVPNIFVKDVLVFHQGQPTN
              :    .  : *    * : :*****:*:       .*: .*::::.::

HEX70a NITY
HEX70b NVAL
HEX70c MNY-
HEX110 DITQ
```
