# Supplementary material for: The four hexamerin genes in the honey bee: structure, molecular evolution and function deduced from expression patterns in queens, workers and drones
Source: BMC Mol Biol. 2010 Mar 26;11:23. doi: 10.1186/1471-2199-11-23 (PMC2861669; doi:10.1186/1471-2199-11-23)
Supplement: Additional file 13 — Specific primers used for sequencing and expression analyses. as title. [file 1471-2199-11-23-S13.PDF]

Additional file 13: Specific primers used for sequencing and expression analyses

Gene	Primer code	Sequence (5'-3')
actin	ACT-R	AGA ATT GAC CCA CCA ATC CA
	ACT-F	TGC CAA CAC TGT CCT TTC TG
rp49	R	TTG AGC ACG TTC AAC AAT GG
	F	CGT CAT ATG TTG CCA ACT GGT
hex 110	0R	CTC GTG AAT CCA AGT GAT GT
	0F	ACG GAC AGT CAA CAT CGC AT
	1R	CAG TTC CTT CAA TCA GAT CAC
	1F	GTT GCT GCT GTA TTA ACT CG
	2R	ATG GTC GTG TTT GGG TCC AA
	2F	GTC CTC AGA ATC TTC AAC TTC
	3R	TGG CCT ACA GGA TTC TGG AT
	3F	TAC TGG TCA CCA ATC CCA AC
	4R	CAT AGG GGA CGT TCA TTC CT
	4F	GGC GGA GGA ATT CAG CAA AA
	5R	ACT AAC AAC TGT TTG CGT GC
	5F	TCC GTA CTG CAA GGA TTA GG
hex 70a	RTR	AAT CGT GAT TCA GAT ACC AGC
	RTF	AAA GCC AAT CAC GCT CTG AT
hex 70b	RT-PCR-R	GTG TTG CTT CCG CTT TTC AGG
	RT-PCR-F	ATC CGC TCT TCA AAT GTG GTC TAC
	RTR	ACG AAG ACA GAT TCG TGG CT
	RTF	ATA CGA CGA GTT CGG TCA TG
hex 70c	PIR	AGA ATA TTG TAA AGC GGT CG
	PIF	GAG GTT TAA TGT GAT TAT CC
	1R	GGG ATA GAT CTC GTA GAT GG
	1F	AGG AAG GCA CAC CGC CGA TA
	2R	ATC CAG AAT TGA TGG GCT TG
	2F	TTA CAA AAC TGC CGC TTG GG
	3F	GCC CAA AGA AAT ACG TGG AC
	JUR	GAA TAT GTC GCT GGG CAT TAT
	JUF	CCA GCC TTT GAC GAG ATC AA
