# Supplementary material for: The four hexamerin genes in the honey bee: structure, molecular evolution and function deduced from expression patterns in queens, workers and drones
Source: BMC Mol Biol. 2010 Mar 26;11:23. doi: 10.1186/1471-2199-11-23 (PMC2861669; doi:10.1186/1471-2199-11-23)
Supplement: Additional file 14 — Accession numbers and references concerning to hexamerins and hemocyanins included in molecular phylogeny analysis. as title. [file 1471-2199-11-23-S14.PDF]

Name	Access ID	Species	Function	Reference
AmeHEX70a	ABQ59246.1	*Apis mellifera*	Metabolism and physiology of adult workers	Martins et al., 2008*
AmeHEX70b	AAT11850.1	*Apis mellifera*	Metamorphosis	Cunha et al., 2005*
AmeHEX70c	ABQ84439.1	*Apis mellifera*		
AmeHEX110	ABU92559.1	*Apis mellifera*	Reproduction	Bitondi et al., 2006*
NviHEX94	XP_001604915.1	*Nasonia vitripennis*		
NviHEX83	XP_001607012.1	*Nasonia vitripennis*		
NviHEX81	XP_001607029.1	*Nasonia vitripennis*		
NviHEX109	XP_001603025.1	*Nasonia vitripennis*		
NviHEX102	XP_001599110.1	*Nasonia vitripennis*		
NviHEX79	XP_001600430.1	*Nasonia vitripennis*		
NviHEX75	XP_001605292.1	*Nasonia vitripennis*		
CfeHEX2	CAB62053.1	*Camponotus festinatus*	Sociality	Martinez e Wheeler, 1994*
DmeLSP2	NP_524816.1	*Drosophila melanogaster*	Reproduction and metamorphosis	Benés et al., 1990
DmeLSP1γ	NP_523868.1	*Drosophila melanogaster*		
DmeLSP1β	NP_476624.1	*Drosophila melanogaster*	Reproduction and metamorphosis	Benés et al., 1990
DmeLSP1α	NP_511138.2	*Drosophila melanogaster*		
Dme7320	NP_649034.2	*Drosophila melanogaster*		
Dme8100	NP_648693.2	*Drosophila melanogaster*		
DmeFBP1	NP_729959.1	*Drosophila melanogaster*	Receptor	Burmester et al., 1999*
Aga22058	XP_321434.4	*Anopheles gambiae*		
Aga22055	XP_321436.4	*Anopheles gambiae*		
Aga9751	XP_321800.4	*Anopheles gambiae*		
Aga16795	XP_311376.4	*Anopheles gambiae*		
Aga29840	XP_001237710.1	*Anopheles gambiae*		
Aga4408	XP_315780.4	*Anopheles gambiae*		
Aga31208	XP_001237381.1	*Anopheles gambiae*		
OatHEX1.2	AAL29455.1	*Ochlerotatus atropalpus*	Expression female-specific - reproduction	Zakharkin et al., 2001*
HceHEX1	AAB86646.1	*Hyalophora cecropia*	Reproduction	Telfer and Pan, 2003
HceHEX2	AAB86647.1	*Hyalophora cecropia*	Reproduction	Telfer and Pan, 2003
HviHEX	AAB14955.1|	*Heliothis virescens*	Reproduction	Telang et al., 2002
HzeHEX	AAR32136.1	*Helicoverpa zea*	VHDL receptor	Persaud and Haunerland, 2004
TniJHSP2	Q06343.1	*Trichoplusia ni*	Basic juvenile hormone-suppressible protein 2	Jones et al., 1993
BmoSP1	P09179.1	*Bombyx mori*	Sex-specific storage protein	Sakurai et al., 1988
BmoSP2	P20613.2	*Bombyx mori*		Fujii et al., 1989
CfuDAP2	AAC35429.1	*Choristoneura fumiferana*		Palli et al., 1998
TmoHEX2	AAK77560.1	*Tenebrio molitor*		
TcaHEX1A	NP_001107851.1	*Tribolium castaneum*		
TcaHEX1B	XP_966959.1	*Tribolium castaneum*		
TcaHEX2	XP_967228.2	*Tribolium castaneum*		
TcaHEX3	XP_973084.1	*Tribolium castaneum*		
TcaHEX4	XP_973044.1	*Tribolium castaneum*		
TcaHEX5	XP_973799.1	*Tribolium castaneum*		
RflHEXII	AAU20852.2	*Reticulitermes flavipes*	Putative receptor	Zhou et al., 2007*
RflHEXI	AAU20851.2	*Reticulitermes flavipes*	Castes diferentiation	Zhou et al., 2007*
LmiJHBS	AAC47391.1|	*Locusta migratoria*	Juvenile hormone-binding protein	Braun and Wyatt, 1996*
CmaHC1	AAW57889.1	*Cancer magister*	Cryptocyanin	Terwilliger et al., 2005
ScuHC1	CAR85691.1	*Sinella curviseta*		Pick et al., 2009
PmaHC1	CAD87762.1	*Perla marginata*		Hagner-Holler et al., 2003
CacHC1	CAR85694.1	*Chelidurella acanthopygia*		Pick et al., 2009
HmeHC1	CAR85695.1	*Hierodula membranacea*		Pick et al., 2009
CseHC1	CAR85697.1	*Cryptotermes secundus*		Pick et al., 2009
CmoHC1	CAR85693.1	*Carausius morosus*		Pick et al., 2009
PamHC1	CAR85701.1	*Periplaneta americana*		Pick et al., 2009
SamEHP	AAC16760.1	*Schistocerca americana*		Sanchez et al., 1998

*See in the text (References)

Benés H, Edmondson RG, Fink P, Kejzlarová-Lepesant J, Lepesant JA, Miles JP, Spivey DW: **Adult expression of the *Drosophila* Lsp-2 gene**. *Dev Biol* 1990, **142:** 138-146.

Fujii T, Sakurai H, Izumi S, Tomino S: **Structure of the gene for the arylphorin-type storage protein SP2 of *Bombyx mori***. *J Biol Chem* 1989, **264:** 11020-11025.

Hagner-Holler S, Schoen A, Erker W, Marden JH, Rupprecht R, Decker H, Burmester T: **A respiratory hemocyanin from an insect**. *Proc Natl Acad Sci USA* 2003, **101:** 871-874.

Jones G, Manczak M, Horn M: **Hormonal regulation and properties of a new group of basic hemolymph proteins expressed during insect metamorphosis**. *J Biol Chem* 1993, **268:** 1284-1291.

Palli SR, Ladd TR, Ricci AR, Primavera M, Mungrue IN, Pang ASD, Retnakaran A: **Synthesis of the same two proteins prior to larval diapauses and pupation in the spruce budworm, *Choristoneura fumiferana***. *J Insect Physiol* 1998, **44:** 509-524.

Persaud DR, Haunerland NH: **Cloning and expression of the VHDL receptor from fat body of the corn ear worm, *Helicoverpa zea***. *J Insect Sci* 2004, **4:** 1-10.

Pick C, Schneuer M, Burmester T: **The occurrence of hemocyanin in Hexapoda**. *FEBS J* 2009, **276:** 1930-1941.

Sakurai H, Fujii T, Izumi S, Tomino S: **Structure and expression of gene coding for sex-specific storage protein of *Bombyx mori***. *J Biol Chem* 1988, **263:** 7876-7880.

Sanchez D, Ganfornina MD, Gutierrez G, Bastiani MJ: **Molecular characterization and phylogenetic relationships of a protein with potential oxygen-biding capabilities in the grasshopper embryo. A hemocyanin in insect?** *Mol Biol Evol* 1998, **15:** 415-426.

Telang A, Buck NA, Wheeler DE: **Response of storage protein levels to variation in dietary protein levels**. *J Insect Physiol* 2002, **48:** 1021-1029.

Telfer WH, Pan ML: **Storage hexamer utilization in *Manduca sexta***. *J Insect Sci* 2003, **3:**26.

Terwilliger NB, Ryan MC, Towle D: **Evolution of novel functions: cryptocyanin helps build new exoskeleton in *Cancer magister***. *J Exp Biol* 2005, **208:** 2467-2474.
