# Supplementary material for: The four hexamerin genes in the honey bee: structure, molecular evolution and function deduced from expression patterns in queens, workers and drones
Source: BMC Mol Biol. 2010 Mar 26;11:23. doi: 10.1186/1471-2199-11-23 (PMC2861669; doi:10.1186/1471-2199-11-23)
Supplement: Additional file 15 — Multiple alignment used for phylogenetic tree reconstruction. as title. [file 1471-2199-11-23-S15.DOC]

Aga22058 ----------------------MRSFLAITVVCLLACTASGY------YMT--KEVKYADKPFLEKQKAM
Aga22055 -----------------------MKGLTVVALLSLAALAAGY------VVVPEGKVTYADKDFLVKQKQM
Aga9751 -----------------------MRTFALFVLGAALAVASGA------FVPSTSQVKYAEKDWLHKQEDL
DmeLSP2 ------------------------MKSFTVIALAAVALLATL------GQAKHLDSKVADKDFLMKQKFM
Aga29840 ----------------------MKLLILAVAISLAVLASGSY------VPSTKFEAKYADKEFLFKQKFF
OatHEX1.2 ----------------------MKLVVAAVAISLAAMASGSF------VSSVGKDIKYADKDVLVKQKFF
Aga16795 ----------------------------------------------------------ADKDFLFKQKFF
Aga4408 ---------------------------------------------------------WEIKLRKTIEDGF
DmeLSP1b -------------------------MKIAIALLACLGLAAAA------SFHQTHEVKIADKAFLMKQKFL
DmeLSP1a -------------------------MKFAIAFLACVAVVTAT------AYHKTHDIKVADKAFLMKQKFL
DmeLSP1g --------------------------MKLTLVILALVACVTA------FSVPTQKVKIADKNFLEKQKFL
Aga31208 ---------------MQRPGCWVVVLGTVLSFCHGTYVPITQ------AKVDGLPVKYADKEFLVKQKFF
BmoSP2 -------------------MKSVLILAGLVAVALSSAVPKPS------TIKS----KNVDAVFVEKQKKI
HmeHC1 -----------------------------------MGLVKYT------------GSASADKDFLVKQRQI
PamHC1 ---------------------MKWQLVTFLGVALCYLASAKL------------SEVPADKEFLVRQREV
CseHC1 ------------------MKCILTVALLGVAVCHLAQAKVMR------------GEAPADKIFLSKQREV
CmoHC1 -------------------MQWVIAAVLLAGLGQLVAAKYTR------------GEVPADKDFLLKQREV
SamEHP --------------------MRLMWLAVLVVAVALGGAQAGI------------GDVPADKPFLMKQREV
CacHC1 -----------------------MKLIPACLVVAVCIAYCCG------------VSVPADSGLVVRQYEI
PmaHC1 -------------------MKWLLTLGLVMVLASLAQAKLTR------------GSVPADQDFLTRQRDV
ScuHC1 -------------------------MARFALTLIACTLVVAV------------AATPADQEFLTKQKEI
CmaHC1 ------------------------------MKWAVVAAVLLV------------AAVAEGADLAHRQQAV
NviHEX109 ----------------------MRFSIVFLALFALAAAIPTTPKLH-----------AADQDILKKQQHV
NviHEX102 ----------------------MRFSIVFLALFALAAAIPATPKYHA------VFKISADQELLKKQQDV
AmeHEX110 ----------------------MRYFIILLALVALGVCAPN------------VKQRAADQDLLNKQQDV
AmeHEX70c ---------------------MLSKVVLLVALAAICGAQG----------ASYAGRHTADMDFLHKQKKI
CfeHEX2 ----------------------MLKELLLLALVALCTTKG----------LHITG-KTADLDFLHKQKKL
NviHEX94 ----------------------MLRKLLVFALVAVSLAAASFEHAKYDMEHTKYDMKMTDKNMLMKQKKI
NviHEX83 ----------------------MMRNCIVFALFVAMAAAG--------QSYPGFYSKTADMEFLHKQKKI
AmeHEX70a -------------------MFIPSHQVWLVGLLAFSLVGA-----------EYYDTKTADKDFLLKQKKV
NviHEX81 -----------------MIGRLALLSVLAVALVAGQAYKP-----------DYYNEVTADKDFLLKQKRV
AmeHEX70b --------------------MIVIMKAGFLFLASLCLLVQ------------AVPNKVADKTYVTRQKNI
NviHEX79 --------------------MTSHRGVLSTGLFAITITIIAG-------LVRASEIPSAEADFLRKQEVV
NviHEX75 -----------------------MKRFAACLVLAIILTGVAA-------QEDVYLVK--DRQFLRRQLFM
TcaHEX1A -----------------------MRFLLVALLAGLCAVSAVS-----------VGSEYHHEYNQQRQKEI
TcaHEX1B -----------------------MRFLLVAALAGLCAVSAVS-----------VENDTYKYS-QEKQKEI
TcaHEX2 -----------------------MRFILVAVLAGLCAVSAIS-----------TKSSNYSQQ----QRDV
TmoHEX2 -----------------------MRLILVALLAGLCAVSAVS-----------VHSTNYAQK----QKDV
TcaHEX3 -------------------------MKLLVAFLALCAFASSY-----------PQQQISDKGLLQKQKDV
TcaHEX4 ------------------------MRFIVVAILGCCALALAF-----------PERKITNKQFLERQRDI
TcaHEX5 ------------------MKIVVIFLCLCGSISSLQEYGVGQ-----------RKFKVSDKLFLERQRDL
RflHEXII ------------------MRTAVLLVFLATAALAAANPSPSQ-----------ESRIIADKQFLQRQNDF
RflHEXI -------------------MNTALLFATVVAVLVCGAFSDHH-----------VGKKVADKPFLMKQKNI
LmiJHBS -----------------------MAALCNLLLLLLLAAAATA-----------APNPEGEKVFLTRQRDV
HzeHEX ----------------------MRAVLLILASLAAVAMARPE---------LDDNTSMGNMDIKHRQLVI
TniJHSP2 ----------------------MRAVLLFVVSLAALRMARPE---------IDD-TTLVTMDIKQRQLVI
HceHEX1 ----------------------MRAAVLLVLGLAAIASARPD---------NDD-VNFVSMDIKQKQLFI
HceHEX2 ---------------MRVLVLVAFLAAATASVVNDANYSFNV---------VIGKDSLMKADVKMKEICI
HviHEX ----------------------------------------------------------------------
BmoSP1 -----------------MRVLVLLACLAAASASAISGGYGTM---------VFTKEPMVNLDMKMKELCI
CfuDAP2 -------------------------MAVASASVVKDSRFDDT---------VIGRETLVNVDVKVKELCI
Dme7320 ----------------------------------------------------------------------
DmeFBP1 MSLEDLQLQKFILDIVQNIRQPLQQSELINLDVGLIADSQRYRGGIDKVMHNVIDLDRQRRLLDEHQVYS
Dme8100 ----------------------------------------------------------------------

Aga22058 LEIFQHVHQP-------ELHTHLWDDQK---AFDLAAYKEHFTKPQVVEEFLRFYKYGLLPMEEIFSVYN
Aga22055 LEVFQHVHQH-------EVHTELWEVSK---AYDIEQHYDQYSNVEAVKEFVTLYKHGLLPFDEIFSVFD
Aga9751 LLLFRHLYEK-------DWNPQLVAYAKNFHFKESAELFQHEPTVQWAKKLWSYYEHGLLPKGEVFSVYD
DmeLSP2 YQILQHIYQD-------DVFTTPFGGSY--VEYKPWEHVADYVHPEMLEHFFELWQHQPFTDDMVWSVMY
Aga29840 FEVLRNIHLP-------LKYDEYIPYTK---TWVSDETKYNDFAQ-VAEFFDYYKTGAFLEKGELFSIYN
OatHEX1.2 FEIMRNIHMP-------LQYEEYNMYTK---TWAEDSSMYQNYDQ-VTEFFNMYNMDLFLGKGEIFTVYN
Aga16795 FEVLRNIHLP-------LQFEEYFPYTK---SYISDESKYVNFQE-VVEFFNYYKAG-FLGKGELFSIYN
Aga4408 YMTDDGQRID-------LRLPESADAFG---SIMNVNVDSYGMKH-MGYIEILSRALLSNTEYRSKKMWP
DmeLSP1b FEIVYRVEDP-------LMFEDHIKQGE---KFYFEESYYTHYDMYMKKFFEAYKAHALLPKGEFFGALV
DmeLSP1a FEIVYRVEDP-------LMFEEYIAMGK---QFYFDKEHYTHFDLYMEKFFEAHKAHALLPKGEFFGALV
DmeLSP1g FEIVHRIDEP-------LMFEEWIKMGQ---KLITDKAQYETFDFYMEKLWESYKLGALLPKGEFFGALV
Aga31208 FEILRHLHQP-------IAFEEYLPYTS---RWVTDP--SKYTNYTEVAEFIQTYEQGVLKKGQIFTIYN
BmoSP2 LSFFQDVSQL-------NTDDEYYKIGK---DYDIEMNMDNYTNKKAVEEFLKMYRTGFMPKNLEFSVFY
HmeHC1 LRLCNKVHE--------PNRFKEQAELGKNYEPLKHLDKYKHP-RSVKTLVKYYNSGLTLPRGAIFTLFN
PamHC1 LRLLNKVHE--------PNRFKEQAELGRSYDPTAHFSKYKNA-VPVKRMVKEYMAGHMLPRGAIFTLFN
CseHC1 LKLLNKVHE--------PNRFKDQADLGRSYDPTQHLSKYKNA-LPVKTLVKQYTSGRLLPRGRIFTLFN
CmoHC1 LKLLNKPHE--------PNRYKDQADSGSKYEPSNNLNRYKNP-VPVKTLVKRYNSHSLLPRGQIFTLFD
SamEHP LRLFNKVHE--------PNRYKEQVELGNAYEPLNSLPRYRNP-APVKQLVRLYRAGSLLPRGAIFTLFD
CacHC1 LKLLNKVHE--------PNRIKEQIEIGNSFDLSTALTGFKNP-NAAKALLKGYKAGSLLPRGSIFTLFN
PmaHC1 IRLCMKVHE--------HNHYQEQVDLVKDYDPSVAG-KFKDV-TPIKRLMKYYNAKTLLPRGDIFSLFH
ScuHC1 VKLLNKVHE--------LNFYQDQATIGKDWDPLAHLDSYKNV-RVVKELVKELKNGKLIKRGEIFNLFN
CmaHC1 NRLLYKIYSPI------PSAFAKLKEHATTFNPRDHISHCNDGGNSVNHLMDEIEAHRVLEQKHWFSLFN
NviHEX109 IHLLENIVG--------ELPHQYLYEIGYKYEFEPYLQNYENP-SVVKYYLGLVKTGHVQPKGTPFSVSV
NviHEX102 IYLLENIAG--------ELPNQHLREIGYSYEFEPYLQNYENP-STVKYYVSLVKTGHAQPQGTPYSDAV
AmeHEX110 IQLLQKISQ--------PIPNQELQNLGASYDIESNSHQYKNP-IIVMYYAGAVKAGLVQPQGTTFSNSI
AmeHEX70c FDLLLYVRQA-------DLSDAEWYDVGRNYDMESNMDMYKDK-NVVQKFLWWYKQGMFLSRNAIFTPLN
CfeHEX2 YELLFFVKQN-------TLTDMEFHKIGRDYNIESNIDSYNDK-LIVQDFLHLHKS-GMLSRNAIYSPYY
NviHEX94 FDLLMYINNQ-------VLTDAEWFEMGRNYDIMSNIDYYTKK-EVVMDFMAHYKF-GMLRKDSIFTYYN
NviHEX83 FDLLMYVDQN-------VLTDTEYFEFGRNFDIAANVDYFTNK-EAVHDFLVYFKS-GFLAKDALFTYYK
AmeHEX70a YNLLYRVAQP-------ALANITWYNEGQAWNIEANIDSYTNA-AAVKEFLSIYKH-GMLPRGELFSLYY
NviHEX81 YEILYHLTQP-------EIKADLYK---EGQEYKIEMDGYTNK-EAVTEFLHRLEH-GYLHHDAVYSIYY
AmeHEX70b YELFWHVDQP-------TVYHPELYQKARTFNLVENLDNYNDK-EAVNEFMQLLKH-GMLPRGQVFTMMN
NviHEX79 FQLLWSSREA-------PKNHPEVFERLRNYKLEDVGAACENH-THAKEFENHLHRFQAVSQDEPFAAFR
NviHEX75 YQLLWKPKDL-------QASFPNVMSKAANFKVEDLLTANVDQ-DAAQAYIKVYN-NGILPRGRSASTFY
TcaHEX1A YRLFKYINQP-------SYYP-DHIEIANTYKFWEHKADYVKP-EVVDEFYNYYYYGKYLHRGEIFSVFH
TcaHEX1B FRLFKYINQP-------SFYP-DFIEVAQSYNFYDFKADYIKP-EVVEEFYRFYYYGKILQRGEIFSVFY
TcaHEX2 WRLFKYINQP-------SYYK-DHVEIAHSYYFYDHASNYAKH-EVVEEFYRYFKYDTFLQRGEIFSVFH
TmoHEX2 WRLFKYINQP-------SYYK-DHVEIAHNYHLSKNVAAYSKP-EVVEQFYKYWQYGKLLPRGEIFSVFH
TcaHEX3 LRLFRYVNQP-------SYYK-DFNEIAQNFDLEQNYNNYANP-EVPKYYYQIYQYG-LLPRGQVFSVFY
TcaHEX4 LRLFRHINQP-------SYYK-DHQEIAQSFHLHDHYDHYTKP-EIAKHYHQIYEYG-LLPRGEVFSVFY
TcaHEX5 LDLYRNVNQL-------DFDE-NRQKIAKSYAIEANVDAYVNS-TAVKQFVSYYKLG-LLPRGEIFSIFY
RflHEXII LRLLVRIEQP-------NYYE-DQVTLGNSYDIEVNIKNYKHP-QVVKQFLSAYKKG-FLPRGVPYSPYY
RflHEXI LGLVHRIHQD-------NLFK-EQVDVGNTYDIEAHISNYKNT-KVVKEFISYYKKG-MLQRWEPFSVYY
LmiJHBS LRLFVKIQQP-------AVIP-EHIEIIKSFKWEEIEPKLQDK-ETVERFLQLHKEGALLSREDDFSILY
HzeHEX LKLLNHITEP--------LMYKDLEDWGKNFKIEDNMELFTKT--DVVKHFIKMIKTGVLPRGEIFTLHI
TniJHSP2 LKLLNHVVEP--------LMYKDLEELGKNFKIEENTDLFTKT--DVLKDFIKMRKVGFLPRGEIFTLHV
HceHEX1 LKLLNHVTEP--------VMYKDVSEIGMNFKIEDNMELYTKT--EVVKSFVNMLKVGVLPRGEVFTLNV
HceHEX2 MKLLNYVLQP--------TVYEDIKEVAREYMLEENTDKYSKS--DVVTKFMETFKMGMLPRGEVFVHTN
HviHEX ----------------------------------------------------------------------
BmoSP1 MKLLDHILQP--------TMFEDIKEIAKEYNIEKSCDKYMNV--DVVKQFMEMYKMGMLPRGETFVHTN
CfuDAP2 MKLLNHILQP--------TLYEDIRTVAREWNIEDNTDKYLKT--DVVKNFIETFKMGMLPRGEIFVHTN
Dme7320 ----------------------------------------------MTRFYEDFSLGKLLHRNVRYNPLY
DmeFBP1 VGRLEHVQQLRGIYRLLARAQDFDTLRRNVVYLRRNINPVLLVNALALAIRDREDTQALIVPAVQELLPE
Dme8100 ---------------------------MGSQLNDDPDSYKEGTWRYIKDFRERVHQNRVPGPFVILNQLD

Aga22058 EYHREQAVALFHLFYYAKDWDTFYKTMVWARFH-VNEGMFVYAVTVAVLHRADMQGIVLPAPYEIYPYYF
Aga22055 DHHREQAIALFHLFYYAKDWETFYKSVVWARFH-VNEGMFVYAVTVAVLHRKDLAGLELPAPYEIYPYYF
Aga9751 ELHREQAIALFRVLYHAKDWETFYKVAAWARYY-VNEGMFVYALTVAVTHRADMAGFVLPAPYEISPYHF
DmeLSP2 DKHEEYVVGLVRLFYFAKNWETFQHVVYWARQH-VNKQLFVYAVTIASLFRDDMQGVVLPAHYEIHPWSY
Aga29840 EQYLRQTYAST-----------------------------------------------------------
OatHEX1.2 KYMLKQTYMLFSFLYNSLDWDTYYKNVIWAREH-INEGMFIYAVTLTVLHRTDLQGIVMPAIYEIYSYYF
Aga16795 QEYMKQTYLLFTFFYNSVDFDTFYKNVVWAREN-VNEGMFVHAITMAVFHHPQLKGFVLPAVYEIYPYYF
Aga4408 SVLMQFETSLR-----------------------------------------------------------
DmeLSP1b MSHAKQARGLFNFFYYAKDWETFAANVAWARMH-VNEGMFVYALTMAVIHRNDFHGLMLPSIYEIFPQFF
DmeLSP1a KHHAKQARGLFNFFYYAKDWETFMTNVAFARMH-FNEGMFVYALTLAVIHRDDFHGLVLPAIHEIFPQFF
DmeLSP1g KTHHKQAYGLFNFFYYAKDWETFVRNVAWARIH-VNEGMFVYALTLAVIHKPEFEGLILPQIYEIFPQYF
Aga31208 YWYAKETVQLYRFFENAIDWDTYYKNVVWARAN-LNEGMFLSALTLSVLHRKDLQGIVLPAIYEIHPHLF
BmoSP2 DKMRDEAIALLDLFYYAKDFETFYKSACFARVH-LNQGQFLYAFYIAVIQRPDCHGFVVPAPYEVYPKMF
HmeHC1 DKHREQMITVFESLFFAQDWDTFLRTACWCRDR-VNEGQFVYALSVAVLHREDTRGIVLPPPYEIYPHLF
PamHC1 NKHREEMITVFESFFFAEDWDTFYRTACWARDR-VNEGQFIYALSLAVLHREDTRGIVLPPAYEIYPHLF
CseHC1 DRQREQMITIFESLFYAEDWDTFYRTACYVRDR-VNEGQFVYALSVAVLHREDTRGMVLPPAYEVYPHLF
CmoHC1 DKHREEMVLLFESMFYALDWDTFYKTACWARDR-INEGQFIYALSVAVIHRDDAKGIVLPPSYEIYPHLY
SamEHP DTHREQMILLFESLLYANDWETFLRTAAWARDR-VNEGQFVYALCVAVLHREDTRGVVLPPPYEIYPHLF
CacHC1 EKHRSEMILLFETFFYANDWDLLFKTACWARDR-INEGQFVYALSVAVLHRSDTHGIVLPPPYEIYPHLF
PmaHC1 KEHREEMILLFESFLFAQDWDTFFKTAVWARDR-INEGQFVYALSVAVLHREDCKGIILPPAYEIYPHMF
ScuHC1 EEHRREMILLFETLFFAKDWDTFHKTACWARDK-INEGQFVYALSVATLHRADTRGIRLPPAYETYPHLF
CmaHC1 ERQREEALMMVDVLLNCMDFETFQGNAAYFREH-MNEGEFVYAIYVAVTHSELMQGVVLPPMYEVTPHMF
NviHEX109 SQLRKEVALLTQIFLGAKDYETFLNTAAWARVY-VNQDQFVKAFSSAVIQRPDTQGVILPPAYEVYPHYF
NviHEX102 SQLRKEVALLVQIFLGAKDYETFLNTAAWARVH-VNQDQFVNAFAKAILQRKDLYGVILPPAYEVFPYYF
AmeHEX110 SQLRKEVSLLYRILLGAKDYQTFLKTAAWARVH-VNEGQFLKAFVAAVLTRQDTQSVIFPPVYEILPQHH
AmeHEX70c SEQKYEVRMLFELLYNAKDFQTFYKTAAWARLR-MNSGMFTTAFSIAVLYRPDTKYMKFPAIYEIYPNYF
CfeHEX2 EEHREEVKIVTKLFFSAKDFQTFYKTASWARLN-LNDGVFTSAFTTAVFYRPDCKHMRLPAPYEIYPNLF
NviHEX94 QEHREEMMTLYKLFYFAKDFNTFYKTACWARIH-MNTGLFTTAFTTAVLYRDDTKFIRLPAIYEMYPNMF
NviHEX83 QEHREEMMLLYRLFYFAKDFTTFYKTACWARIH-INPGMFTAAFTTAVIYREDTKHIRLPAIYEIYPNLF
AmeHEX70a PQLLREMSALFKLFYHAKDFDIFFKTALWAKNN-INEAQYIYSLYTAVITRPDTKFIQLPPLYEMCPYFF
NviHEX81 PRNVDETRAVFRILFYAKDFDTFYKTALFCRNR-LNHQMFGYAFYLAVIHRPDTKYIKLPPLYEIAPHYF
AmeHEX70b KEMRHQAVVLFRLLYSAKTFDVFYNTAVWARFN-VNEQMYLYALSVAVIHRPDTKLMKLPPMYEVMPHLY
NviHEX79 DEHVDEVRLLFDVLLCAKSFDEFAALVAWSHAGKVPQVAWIHAFAMALAHRKDTRGLRLPNLYEIVPHYF
NviHEX75 PPHLAEIKALFNVLYTSKDFETFAVVSTWSRD-------------VTSLTRHDTRRVRIPNVHVTLPSYF
TcaHEX1A KEHLYQAIALFKLFYYANTYESFYHTAVWARNY-VNEGMFLYAVSVALVHRPDTYYYALPPIYEVYPYYF
TcaHEX1B KEHLRQAIALFKLFYYANTWETFYKTAVWARNN-VNEGMYLYSLSVALIHRPDTYYYALPPIYEVYPYYF
TcaHEX2 GEHLKQAIALFKLFYYANDFDTFYKTAVWARQH-VNEGMFLYAFSVALIHRPDTYYFSLPPIYEIYPHYF
TmoHEX2 GEHLKQAVALFKLFYYANDYETFYTTAVWARNH-INEGMFMYSLSVALIHRPDTYYFSLPPIYEIYPHYF
TcaHEX3 QEHLQQAIALYKLFYYAKDYQTFYNTAVWARQY-INEGVYLYSLSVAIVHRNDTYGVVLPPIYETYPYYF
TcaHEX4 EEHLQQAIALYKLFYYAKDYDTFYRTAVWARQH-VNEGVFLYSFSVAIVHREDTYGIVLPPIYEIYPHYF
TcaHEX5 YEHRLQAISLFKLFYYARDFDTLYKTAVWARNN-VNEGLFLYAFSTALVHRDDTFTFVLPPIYEIYPFYF
RflHEXII TTQSYETKLLFDLFYYANDYDTFYKTAVWARDR-INAGQFLYAFSVATFLREDLNDIVLPPPYEVYPYLF
RflHEXI KTHLEQAISLFELFYFANDFDTFYKTACWARDR-VNPLMFWYSFTAAVLHRDDTTDVMMPPPYEVYPYFF
LmiJHBS AKHLWQAVDLFKILYRIPDYETFHKVAVWLRYN-VNVGLFEYVARVVLLHRDDTRELLLPPPYEVLPQFF
HzeHEX DRQLKEVVTMFHMLYYAKDFNTFIKTACWMRLH-LNEGMFVYALTVAVRHREDCKGIILPPPYEIYPYYF
TniJHSP2 DRQLKEVVTMFHMLYYAKDFTTFVKTACWMRLY-LNEGMFVYALTVAVRHREDCKGIILPPPYEIYPYYF
HceHEX1 DRQMKEVVNMFHILYYAKDFSTFIKTACWMRLY-LNEGMFVYSLSVAVRYREDCKGVVLPPPYEIYPYYF
HceHEX2 ELQMEQAVKVFRILYFAKDFDYFIKTACWLRER-INGGMFVYALTAAVFHRSDCVGITLPAPYEIYPYFF
HviHEX ----------------------------------------------------------------------
BmoSP1 ELQMEEAVKVFRVLYYAKDFDVFMRTACWMRER-INGGMFVYAFTAACFHRTDCKGLYLPAPYEIYPYFF
CfuDAP2 EKHIKEAIRVFKLLYFAKDFDVFIKTCCWLRER-INIGMFVYTLTAAVFHRNDCRGIILPAIYEIYPYYF
Dme7320 ADHYRQMLGLYQFFYNARDWYTLWQNISWARVH-VHPCIFVQALTQLILKREDYQALIMPKIYELWPESY
DmeFBP1 LYLDEEVIQQVRSVLREQTQRPSLMDIVGMRQRAMNPVMSILMPWREIHMQMALRKQQNIQNVLGQKRVV
Dme8100 EEMPLNLLGIYRFLTMAVDWTSFQRNACFARIH-FHPLLFVNALQMAVEEREDTKDLRMPAMYEVLPQLY

Aga22058 FNDVVISKAQRYKMQGFYR---------------------------------------------------
Aga22055 FNTEVVQKAAQYKMQGFPG---------------------------------------------------
Aga9751 INTEVIQKAQQYKMQGYYG---------------------------------------------------
DmeLSP2 FDSQALEWAEHYKMHGFHH---------------------------------------------------
Aga29840 ----------------------------------------------------------------------
OatHEX1.2 FNTDIIRTATYRKMYDPK----------------------------------------------------
Aga16795 FNTDLIHSVDQYLYDFVTGKNTIVRNSRDFYYSVQQFSTYDYTYSCGVGSGSKYIDDLPFGYPFDRDIDF
Aga4408 ----------------------------------------------------------------------
DmeLSP1b FNSKFVFEAEKFDYEMWMKMTMYEKEYMDVYYKTN----------------------------GYDYSTM
DmeLSP1a FNSKFVMEAEKFDYEMWMKTSLYEKEYMDVYHKIPTFSSYEHGYKQGMAYGYGKTHGHGQTY-EHEFGSM
DmeLSP1g FNSKFVYAAEKFDYEVFSKLTMYEKEYKDILYKDYS---------------------------------E
Aga31208 FDGDVFQDATNKRALD------------------------------------------------------
BmoSP2 MNMEVLQKIYVTKMQHGLINP-------------------------------------------------
HmeHC1 VNSEVIHAAYKAKMRQEP----------------------------------------------------
PamHC1 VNSEVIHAAYKAKMRQEP----------------------------------------------------
CseHC1 VNSDVIHAAYRAKMRQEP----------------------------------------------------
CmoHC1 VNSEVIHAAYKAKMRQEP----------------------------------------------------
SamEHP VNSEVIHAAYKAKMRQEP----------------------------------------------------
CacHC1 VNSEVIHAAYKAKMRQEP----------------------------------------------------
PmaHC1 VNSEVINSAYKAKMTQTP----------------------------------------------------
ScuHC1 VTSQVIHEAYAAKMRQEP----------------------------------------------------
CmaHC1 TNSEVIDKAYAAKMTQTA----------------------------------------------------
NviHEX109 FDTRIIQKVHDYMNQYGY----------------------------------------------------
NviHEX102 FDTRIIQKVQDYVNKYGL----------------------------------------------------
AmeHEX110 LDSRVIQEAQNIAIQN------------------------------------------------------
AmeHEX70c FDSSVIEEAQNLKMSRGS----------SV----------------------------------------
CfeHEX2 FDNNVIQRARDIKMMHG-----------VR----------------------------------------
NviHEX94 FDSKVVRDAQRIMMTHGKTHLGHEMMGMKK----------------------------------------
NviHEX83 FDAKVIRDAHRLKMTRGYGYHGYGHPMPFA----------------------------------------
AmeHEX70a FNSEVLQKANHALIFGK-----------LD----------------------------------------
NviHEX81 YNSELFEKAHHVKAFGK-----------LE----------------------------------------
AmeHEX70b FNDEVMQKAYNIAMGDT----------ADM----------------------------------------
NviHEX79 FETEVMVKAYRAKTGEI-----------------------------------------------------
NviHEX75 YNSYVFTKFNETQDDDQ-----------------------------------------------------
TcaHEX1A YSSEVIQEAQYYKQMYHGADG-------------------------------------------------
TcaHEX1B YNSEVIQEAQYYKQMYSSQSG-------------------------------------------------
TcaHEX2 YNYEVIQKAQHYKQMYYGQDG-------------------------------------------------
TmoHEX2 YSTEVIKKAQYYKQMYSGQQG-------------------------------------------------
TcaHEX3 YNNEVIQQAQRYKQQYD-----------------------------------------------------
TcaHEX4 YNNEVIQEAYRYKQQYYNQ---------------------------------------------------
TcaHEX5 YNSEDLEKAQRYKQAYSGG---------------------------------------------------
RflHEXII VDSDVIQKAYETKMWDHS----------------------------------------------------
RflHEXI VDSDIIQKAYKYWMMHVG----------------------------------------------------
LmiJHBS VSAPALQQATDACLRGLCAD--------------------------------------------------
HzeHEX VRADVIQKAYLLKMKKGDVDLKLCDFYG------------------------------------------
TniJHSP2 VRADVIQKAYLLKMKKGLLDLKLCDFYG------------------------------------------
HceHEX1 VRADVIQKAYLIKMRKGLLDLKLSEFYG------------------------------------------
HceHEX2 VDSHVINKAFMMKMTKAASDPVLMNYYG------------------------------------------
HviHEX ----------------------------------------------------------------------
BmoSP1 VDSHVISKAFMMKMTKAAKDPVLWKYYG------------------------------------------
CfuDAP2 VNSDVINKAFMMKMTKKVTDNVLANYWG------------------------------------------
Dme7320 HDDVTVRKARNFNFANWIR---------------------------------------------------
DmeFBP1 VRAENQGQVEGTSLLTDDIELQNFVQNLIQELALLEDVTQLEQSQLINNDQREEQWVGRQRVQYQDNNQE
Dme8100 FEKEVILTAQEVTWNQLSPIR-------------------------------------------------

Aga22058 ------------------------------------------------MKKADGVYSAFIPSNYTGYYVH
Aga22055 ------------------------------------------------VKKVDDVYTVVIPTNYTGWYVH
Aga9751 ------------------------------------------------MKKVDGVYTVTIPSNYTGWYMH
DmeLSP2 ------------------------------------------------VKQMDNIYNVVIRTNYSNVHGS
Aga29840 ----------------------------------------------------------------------
OatHEX1.2 ------------------------------------------FGFHANGKYNVVYSNFTMTFPERYKDTF
Aga16795 SYFYTKNMYFKDVTIYHSDEVKQYFFNYYKAGFLGKGELFSIYNQEYMKQTYLLFTFFYNSVDFDTFYKN
Aga4408 ----------------------------------------------------------------------
DmeLSP1b YRSSDYTYMKDFKTWQWWKLMGLGEHWYTEDKFILRENIYEFNQETKWLSMMKDVKKFYMPVDYTRDLNL
DmeLSP1a YQTSDYMYMKDFKTWQWWKLMGLGEHWYSESNYILRENIYEYNQESNWLTMMKDVKKFYMPVDYSRDLYL
DmeLSP1g FTGNFYFYTKDWKTWQWYKMMGLDQEWYVEDKYFLRENLSQFVNDPKYVDVVKGLKKFYMPVDYTRDIDF
Aga31208 ------------------------------------------------------PNYGFYANKRNNLALA
BmoSP2 -------------------------------------------EAAAKYGIHKENDYFVYKANYSNAVLY
HmeHC1 -----------------------------------------------------------AIVRMNFTGTI
PamHC1 -----------------------------------------------------------AVVRMNFTGTI
CseHC1 -----------------------------------------------------------AVVRMNFTGTV
CmoHC1 -----------------------------------------------------------AVVRMNFTGTI
SamEHP -----------------------------------------------------------AVVHMNFTGTI
CacHC1 -----------------------------------------------------------AIVRMNFTGTI
PmaHC1 -----------------------------------------------------------AIIHMNFTGTI
ScuHC1 -----------------------------------------------------------AVIHMNFTGTV
CmaHC1 -----------------------------------------------------------GEFYMSFTGSK
NviHEX109 ----------------------------------------------EHHGEGEQHHEHVHHIHVNYTSYL
NviHEX102 ----------------------------------------------DYQQAQG-EKYGVYTINVNYTSYY
AmeHEX110 --------------------------------------------------TQGKNNQQNILIPVNYSALL
AmeHEX70c ----------------------------------------------VTGMNNIETYIVNTNYSSKYMREY
CfeHEX2 ----------------------------------------------PTNVDNGDSYVIHANYSGNLVRPY
NviHEX94 ----------------------------------------------TDDMNHVETFYLYSNFTEVCLNPM
NviHEX83 ----------------------------------------------KGSFENVETFYVYSNFTDVCINPT
AmeHEX70a ----------------------------------------------TKTSGKYKEYIIPANYSGWYLNHD
NviHEX81 ----------------------------------------------QKHSAGYDTYILPYNYSNFYLNDE
AmeHEX70b ----------------------------------------------KKTYNNIDYYLLAANYTGWYLTKH
NviHEX79 -------------------------------------------------EGVGDEYVIEAN----HTSHT
NviHEX75 -------------------------------------------------CGNSDNQIVAKYSKIVSRLDA
TcaHEX1A ----------------------------------------------------AHYNDRTIYANYSGYYLN
TcaHEX1B ----------------------------------------------------AHYNGRTIYANYSGYYLN
TcaHEX2 ----------------------------------------------------AHYNDRTIYANYSGYYVN
TmoHEX2 ----------------------------------------------------SEYNDKIIYANYSGHYLN
TcaHEX3 ------------------------------------------------------GKSYTINANYSGYYLN
TcaHEX4 ------------------------------------------------------EHGYTINANYSGFYLN
TcaHEX5 ------------------------------------------------------AKTYTIYSNYSGYYLN
RflHEXII -------------------------------------------------------LTSPKTHVFPANYTV
RflHEXI -------------------------------------------------------TTEHHTYIIPMNHTM
LmiJHBS -----------------------------------------------------ENRPFVIRANYTGSHAL
HzeHEX -------------------------------------------------IKKTDKDVFIIDENVFDKRVH
TniJHSP2 -------------------------------------------------IKKTDKDVFIIDENVYDKRVH
HceHEX1 -------------------------------------------------IKKTEKDVYIIDENVYDRRVA
HceHEX2 -------------------------------------------------IKVTDKSMVVIDW-RKGVRRS
HviHEX ----------------------------------------------------------------------
BmoSP1 -------------------------------------------------ITVTDDNLVVIDW-RKGVRRS
CfuDAP2 -------------------------------------------------IRVTDKNVCYIDS-RKGVRHT
Dme7320 --------------------------------------------------------------YVNMTDVE
DmeFBP1 RDLG--------------QDVDTGRLLRVSRRRLLEQQDEYQGQNIYGNQKDRFQRLLRRDDDNDDNDDD
Dme8100 -------------------------------------------------LVTPKRRWIDILLGYRNPKQP

Aga22058 SNPEQRVSYFMEDIGLNAYYYYFH--ADYPTWMGG-KE--------------------------YGLYKD
Aga22055 TNVAQKVSYFTEDIGLNTYYYYFH--ADYPFWMGG-KE--------------------------YGLYKD
Aga9751 TNAEQKISYFTEDIGLNSYYYYLH--TDYPFWLGG-EL--------------------------FGLSKD
DmeLSP2 LNYDHDLAYYLEDVGFNAFYYYFN--LDYPFWTKGGEE--------------------------HVLNKD
Aga29840 ----------------------------------------------------------------------
OatHEX1.2 MMNGLGMDYFHEDIGLNSYYFYFM--MDYPFFLGG-DK--------------------------FGLLKE
Aga16795 YYGEDKLSYFTEDIGLNAYYYYFM--MDYPYFVGT-DK--------------------------FNLYKD
Aga4408 ----------------------------------------------------------------------
DmeLSP1b YNKESKLSYFTEDLGWNAYWYYLN--MDYSFFLDG-NT--------------------------FDLKND
DmeLSP1a YNEESKLSYFTEDLGWNSYWYYLN--MDYSFFLDG-KT--------------------------FGLQND
DmeLSP1g FNDETKMTYFTEDLGWNAYWYYLN--MDYAFFLNG-KQ--------------------------FGLDKD
Aga31208 NYTAWFATQF------------------------------------------------------------
BmoSP2 NNEEQRLTYFTEDIGMNAYYYYFH--SHLPFWWTS-EK--------------------------YGALKE
HmeHC1 RNPEQRVAYLGEDVGMNAHHSHWH--MDFPFWWKQQE---------------------------YGVEKD
PamHC1 RNPEQRVAYLGEDVGMNSHHSHWH--MDFPFWWKQEE---------------------------YGVHKD
CseHC1 RNPEQRVAYLGEDVGINSHHSHWH--MDFPFWWKQDE---------------------------YGVKKD
CmoHC1 RNPEQRVAYLGEDLGMNSHHAHWH--MDFPFWWKPEE---------------------------YGIDKD
SamEHP RNPEQRVAYLGEDLGMNSHHSQWH--MDFPFWWKEDE---------------------------YGIRKE
CacHC1 KNPEQRVAYLGEDIGMNAHHAHWH--MDFPFWWKEHE---------------------------YGIHKD
PmaHC1 RNPDQWIAYLGEDVGLNSHHAHWH--MDFPFWWKAAE---------------------------YGIEKD
ScuHC1 RNPEQRVAYFGEDVGMNSHHAVFH--MDWPFWWNEEK---------------------------YGLTKD
CmaHC1 KNPEQRVAYFGEDIGMNSHHVHWH--MDYPFWWHG-------------------------------QEID
NviHEX109 PFGENQIAYFTEDIGLSAYYSYVH--LSSYILHHGQQL-----------------HGYGQKYPQSHHGHV
NviHEX102 PFGENEIAYFTEDIGLNAYYGYVQ--LAHYMIPYG---------------------GDYAHKEHGEHYKN
AmeHEX110 SHDEQQLSYFTQDIGLAAYYAQVN--LAGYIQEQNQQQQQQPLTQQQYQQQIVGKYLQQQAGQQDQQANI
AmeHEX70c NDPEYKLDYFMEDVELNAYYYYMR--EMLPYWMSSSQ---------------------------YHMPKE
CfeHEX2 LD-DYKLDYFMEDVGLNAYYYYVR--QVMPFWLSMKN---------------------------FDIPAQ
NviHEX94 HNYENKMMYFMEDIGLNAFYYYYR--MMFPFWMSTKE---------------------------YSVPQN
NviHEX83 NALEYKLNYFTEDVGLNAFYYYFR--MSFPFWMDSKD---------------------------YEVPKS
AmeHEX70a YNLENKLNYFIEDIGLNTYYFFLR--QAFPFWLPSKE---------------------------YDLP-D
NviHEX81 YDYEQRLNYFTEDIGLNNYYFFFR--NQFPWFLKSEE---------------------------IGAPKS
AmeHEX70b NVPEQRLNYFTEDVGLNHFYFMLN--HNYPPFMLSNS---------------------------LNFP-Q
NviHEX79 ESTDSQLNYFRDDVGLNQFYFQLH--NQLPFWLSCEEK----------------------NCNSNGIPKH
NviHEX75 FDPEALISYFIEDVGVENFYFYQS--ADVPLFMNRPK---------------------------YDIDTY
TcaHEX1A LHPEQALSYFTEDVGVNSFYYYY--NLYYPFWMSGEE---------------------------FNLKYD
TcaHEX1B LYPEQSMAYFTEDVGVNSFYYYY--NLYYPYWMSGEE---------------------------FNLKYD
TcaHEX2 VYPEQALAYFTEDVGVNSFYYYY--NLYYPYWMSGEE---------------------------FNLKYD
TmoHEX2 LHPEQSLSYYMEDVGLNSFYYYY--NLYYPFWMSGEE---------------------------FNLKYD
TcaHEX3 LNPEQSLSYFTEDVGINSFYYYY--NVYYPFWLGGQD---------------------------FNYQND
TcaHEX4 LHPEQSLSYFTEDVGVNSFYYYY--NIYYPHWLGGED---------------------------FDFAHD
TcaHEX5 LNKEQSLSYFLEDVGLNAFYYYC--NIYYPFWMDGDE---------------------------FKLKND
RflHEXII HTPEQVLSYFTEDVGLSTYYLYY--YYNYPTFFNSTE---------------------------YGVHFD
RflHEXI KSKENLLYYFTEDVGLNAFNMYY--RMYYPSWFNVTE---------------------------YGHKFD
LmiJHBS RNVESKLSYFREDVGLASYMAFMGAQYIVPWVNASEC---------------------------PWPALR
HzeHEX LSDEDKLRYFTQDIHLNTYYYYFH--VHYPFWMKDTVMDKN--------------------------LKT
TniJHSP2 LNKEDKLRYFTEDIDLNTYYFYFH--VDYPFWMKDKFMDK---------------------------MKM
HceHEX1 LNEDDKIKYFTEDIDLNTYYYYFH--VDYPFWMKDQIFDK---------------------------LKT
HceHEX2 LSEDDKYSYFTEDVDLNTYMYYLH--MNYPYWLTDEVYGL---------------------------NKE
HviHEX ----------------------------------------------------------------------
BmoSP1 LSQNDVMSYFMEDVDLNTYMYYLH--MNYPFWMTDDAYGI---------------------------NKE
CfuDAP2 LGKDDQLAYFTEDIDLNAYLYYLH--MNYPYWMENEVYGL---------------------------HKE
Dme7320 EIHPQKLEPLNLEGNLRGTIEWFQAMADVNILRMNEQK--------------------------KRNKLE
DmeFBP1 DDQDIRMGRVQLQRGISQGGLRIGGAHNLPTVSVNSDRLLHVS------------------RRRLNAIQQ
Dme8100 WMEEEPIPTDPLIIDNARKVAYLSLDLELNSHWNSLITR-------------------------LIISIE

Aga22058 RRGEFYLYQHQQFLARYYLERLSNDLGTIPTFS-WYEPIVTGYYPYLRYYNGVPFPARENYHNGYT-EKH
Aga22055 RRGELYLFKHQQLLARYYLERLSNDLGTIPEFS-WFKPIVTGYYPNMHYYNGVSFPSRDNYYEVYTPEHY
Aga9751 RRGELYLYEHQQILARYYLERLSNDLGHVPEFS-WWTPIATGYYPDLQYYNGHSFPARDNYYHVDQESNY
DmeLSP2 RRGELYLYVHWQLLARWYLERLSHDLGEVPAFN-MYVPTESGYASNLRTYYGVPQWHRENHHSFYHEHNY
Aga29840 ----------------------------------------------------------------------
OatHEX1.2 RSGELYLYTHQQLLARYNLERMSNMMHPTKMLT-WKFPLETGYFSLLSYWNGIPFKYRDFNYMIKDDDYF
Aga16795 RRGELYMYMYQQLIARYYLERQVNFMGPIEEFD-YDFPIKTGYWSKLSYYNGIPFFVRNDYYSVSKDFYY
Aga4408 ----------------------------------------------------------------------
DmeLSP1b RRGEWWLYNVHQLLSRYYMERLSHGFGEIPEFS-WYQQIEMGYDPQLIYYNGIGYSFRKNYYEMETYANY
DmeLSP1a RRGEWWLYNVHQLLSRYHMERLSHGLGEIPQFS-WFHQIEMGYDPQLIYYNGIGYSYRKNYYELETYGNF
DmeLSP1g RRGEYWIYNVQQILARYYQERLANGFGEIPEFF-WYKQIEYGYDPQLIYYNGIGYSYRKNYYDFYTYGKF
Aga31208 ----------------------------------------------------------------------
BmoSP2 RRGEVYFYFYQQLLARYYFERLTNGLGKIPEFS-WYSPIKTGYYPLMLTKFTPFAQRPDYYNLHTEENYE
HmeHC1 RKGELFYWMHHQLIARFDLERLSNDLPFVEPLS-WEDKIVDGFYPQTTYRVGGEFPARPDNFEFQDLEHI
PamHC1 RKGELFYYMHHQLIARFDAERLSNDLPIVEPLY-WDEKIVDGFYPQTTYRVGGEFPARPDNFEFHDLEHI
CseHC1 RQGELFYYMHHQLIARLDLERLSNDLPFVKPLY-WEDKIEDGFYPQTTYRVGGEFPARPDNFEFQDLQDI
CmoHC1 RKGELFYYMHHQLIARFDLERLSNDLPPVEPLG-WRERIVDGFYPQTTYRVGGEFPARPDDFEFQDLSSI
SamEHP RKGELFYYMHHQLIARFDLERLSNDLPFVEPLY-WTERIKDGFYPQTTYRVGGEFPARPDNFAFHDLQNI
CacHC1 RKGELFYYMHHQLIARFDLERLSNNLPFVEPLS-WDQKIANGFYPQTTYRVGGEFPARPDNYAFHDLENI
PmaHC1 RKGELFYYMHHQMIARYDFERLSNWLHFVEPIS-FEDEIEHGFYPQTTYRVGGEFPARPDNFHFHDLEHI
ScuHC1 RKGELFWYMHHQLITRFDAERLSNDLNEVEALH-WDKPIVEGFYPQTTYRKGGEFPARPDNFMFHDLKDH
CmaHC1 RKGELFFWAHHQLTARFDAERLSNHLPLVDELY-WDRPIKEGFAPHTSYKYGGEFPTRPDNKNFEDVDGV
NviHEX109 GHGAHFYYIHQQLLARYNLERLGQGLEPIRELDNYWEHIETPYKPHLRYLNGVNFPGRDEHHYITP----
NviHEX102 GHGAHYYYIHQQLLARYNLERLGQGLAPIKELDYYYEHIETPYKPNFRYTNGVNFPGRDEHHYITP----
AmeHEX110 GRGAQYLYLHQQLLARYELNRLSNGLGPIKDID--YENVQSLYQPHLRGLNGLEFAGRPQNLQLQS----
AmeHEX70c IRGQLYYFLHKQLMTRYFLERMSNDLGKTAEFD-WNKPINSGFYSTIMYSNGVTFPQRNRFSSLP-----
CfeHEX2 FRGHFYYFKHKQLLNRYYLERMSNDLGDIEDFD-WNKPFYPGFHSILMYNNGMVIPQRSRYTNVP-----
NviHEX94 IRGELYYYFHQQLMARYYLERFSNGLGEIEDFS-WDSMRLPGFYSDYMFNNGVSMPRRDWWNVVP-----
NviHEX83 FRGDFYYFMHKQIMSRYYLERFSNDLGEIEDFS-WDSMSLPGFYSDISFFNGVSMPRRDWWNVVP-----
AmeHEX70a YRGEEYLYSHKLLLNRYYLERLSNDLPYLEEFD-WQKPFYPGYYPTMTYSNGLPFPQRPIWSNFP-----
NviHEX81 YRGEEYFYGHKQLFNRYYLERLSNDLGKVEDFD-WDRPFYPGFWPSLKFPNGLQFPSRNGNSYFP-----
AmeHEX70b IRGEFYFFLHKQVLNRYYLERLSNDMGEVSYVS-LDHPIPTGYYPTMRFRNGLAFPQRETGATVP-----
NviHEX79 LRGELYYHVHKTLLARYELERLSLGLGRVEDID-WEQGISTGYRSGMTHLNGWPVPDREPGARLP-----
NviHEX75 RRGERYFNNHKMFLARYNLERLSHGLGFIEDLD-WNQNIQTGYYPYMADYNGIPFPHRPHNSPVP-----
TcaHEX1A NRGELFYYMYQQILARYYLERLSNGFGEIDHFN-WEVPFETGYYPSLCYPNGLQFPSRPNYAHLYEYFYN
TcaHEX1B NRGELFYYMYQQILARYYLERLSNGFGEIDYFN-WEVPFENGYYPTMCYPNGLQFPARPSYSRLYEYFYN
TcaHEX2 NRGEIFYYMYQQILARYYLERLSHGFGEIDHFD-WEVPFESGYYPSMCYPNGLYFPTRHAYAHLYEYFYN
TmoHEX2 NRGEAFYYMYQQILARYYLERLSHGFGEIENFD-WEVPFETGYYPSLCYPNGLHFPTRPNYAHLYEYFYN
TcaHEX3 LRGELFYYVYQQILARYYLERLSNGFGEIPYFN-YEVPFENGYYPSLQYPNGLFFPQRPNFANLYEYFYN
TcaHEX4 RRGEQYYYVYQQILARYYLERLSNDFGEIPFFN-YEVPFENGYYPLLQYPNGLFFPQRPNYAKLYEYFYN
TcaHEX5 RRGEQYYYLYQQLLARYYLERLSNDFGEVEFFN-YDEPFKYGYYSSLRYPTGLAFPNRPNYAPLSETRAN
RflHEXII RRGEQFYYKIQQVLARYILERLSHDLPEVQPFH-YDKPFQTAYYPKLRYANGQEVPFRPYEYSKRNLYNY
RflHEXI RRGEMFLYVQHQLYARYSLERMSNGMPEVEPFV-YNKPLKTAYNPNLMYHTGQEMPPRPSD---------
LmiJHBS MRGDLYYFLMRNLLARYDLERLSNHMLPVTPVD-LWEPVSEGYDPQLRLLSGKEAAARPEG---------
HzeHEX RRFELTVYMYQQILARYYLERLSNRMGMIKEFS-WHKTIKKGYWPWLKTSNGIEFPVRFNNYVIAHDYN-
TniJHSP2 RRFELTYIMYQQILARYILERLSNGMGMIKDLS-WHKTIKKGYWPWMKLHNGVEIPVRFDNYVIVRDHN-
HceHEX1 RRFELTLYIYQQILARYYLERLSNGLGEIPEFS-FYKPIQQGYWPWLKLHNGIELPARFNNYVSYKYDN-
HceHEX2 RRGEILMYANSQLLARLRMERLSHKMCDIKMFM-WNEPVKDGYWPKIRLPNGDEMPVRQNNFVPVTSEN-
HviHEX ----------------------------------------------------------------------
BmoSP1 RRGEIMMYANQQLLARMRLERLSHKMCDVKPMM-WNEPLETGYWPKIRLPSGDEMPVRQNNMVVATKDN-
CfuDAP2 RRGEVMTYASEQLLARYRLERLSHGMCDIKPIK-WDEKIKTGFWPKIRMHNGEEMPARQGNTILLNETN-
Dme7320 HLLEDIDWQSYWYNLNMGVILTAENSDQLREWCYYQLSQILARYKLECYGQKMAYKRLTQNRGINEEFQF
DmeFBP1 DQQQDQLNGRQRFMGLVRGDRLSEGRRVGQLDDVRQERQNNWQKQDYILTQGRTFGVQQERQNYADQLES
Dme8100 EGKEKTNEPIIIDGDRLVAFRGSFDEVNFKKHLALGSHFHNTKLYLYNLHQFVAALSMEDLATGQKS---

Aga22058 DV--------------------------------------------------------------------
Aga22055 ED--------------------------------------------------------------------
Aga9751 RE--------------------------------------------------------------------
DmeLSP2 EH--------------------------------------------------------------------
Aga29840 ----------------------------------------------------------------------
OatHEX1.2 KL--------------------------------------------------------------------
Aga16795 QV--------------------------------------------------------------------
Aga4408 ----------------------------------------------------------------------
DmeLSP1b DM--------------------------------------------------------------------
DmeLSP1a EM--------------------------------------------------------------------
DmeLSP1g EM--------------------------------------------------------------------
Aga31208 ----------------------------------------------------------------------
BmoSP2 RV--------------------------------------------------------------------
HmeHC1 K---------------------------------------------------------------------
PamHC1 K---------------------------------------------------------------------
CseHC1 K---------------------------------------------------------------------
CmoHC1 K---------------------------------------------------------------------
SamEHP K---------------------------------------------------------------------
CacHC1 K---------------------------------------------------------------------
PmaHC1 K---------------------------------------------------------------------
ScuHC1 R---------------------------------------------------------------------
CmaHC1 AR--------------------------------------------------------------------
NviHEX109 ----------------------------------------------------------------------
NviHEX102 ----------------------------------------------------------------------
AmeHEX110 ----------------------------------------------------------------------
AmeHEX70c ----------------------------------------------------------------------
CfeHEX2 ----------------------------------------------------------------------
NviHEX94 ----------------------------------------------------------------------
NviHEX83 ----------------------------------------------------------------------
AmeHEX70a ----------------------------------------------------------------------
NviHEX81 ----------------------------------------------------------------------
AmeHEX70b ----------------------------------------------------------------------
NviHEX79 ----------------------------------------------------------------------
NviHEX75 ----------------------------------------------------------------------
TcaHEX1A YG--------------------------------------------------------------------
TcaHEX1B YG--------------------------------------------------------------------
TcaHEX2 YG--------------------------------------------------------------------
TmoHEX2 YG--------------------------------------------------------------------
TcaHEX3 YG--------------------------------------------------------------------
TcaHEX4 YG--------------------------------------------------------------------
TcaHEX5 FG--------------------------------------------------------------------
RflHEXII NGQGQYYGNYYGGN--------------------------------------------------------
RflHEXI ----------------------------------------------------------------------
LmiJHBS ----------------------------------------------------------------------
HzeHEX ----------------------------------------------------------------------
TniJHSP2 ----------------------------------------------------------------------
HceHEX1 ----------------------------------------------------------------------
HceHEX2 ----------------------------------------------------------------------
HviHEX ----------------------------------------------------------------------
BmoSP1 ----------------------------------------------------------------------
CfuDAP2 ----------------------------------------------------------------------
Dme7320 IT--------------------------------------------------------------------
DmeFBP1 VSRDDERLVHINRRRLNQDNQDIQQQQMHFPRRINSIGEGRRVMGERPIQDEDILKLIRGENRLKLMTDD
Dme8100 ----------------------------------------------------------------------

Aga22058 -----------------------------------------------------------VQEITDYERRL
Aga22055 -----------------------------------------------------------VEEVVEYEHRI
Aga9751 -----------------------------------------------------------IRQVVDYEKRL
DmeLSP2 -----------------------------------------------------------IEHVEMYTQRV
Aga29840 ----------------------------------------------------------------------
OatHEX1.2 -----------------------------------------------------------DWIMD-WEMRM
Aga16795 -----------------------------------------------------------NLLKD-YEMRI
Aga4408 ----------------------------------------------------------------------
DmeLSP1b -----------------------------------------------------------LDKITGFLKRI
DmeLSP1a -----------------------------------------------------------LDKITGFQQRI
DmeLSP1g -----------------------------------------------------------YSQIQNFFSRV
Aga31208 ----------------------------------------------------------------------
BmoSP2 -----------------------------------------------------------RFLDTYEKTFV
HmeHC1 -----------------------------------------------------------VKDMVDYTRRI
PamHC1 -----------------------------------------------------------VKDMMDYTRRI
CseHC1 -----------------------------------------------------------VKDMVDYTRRI
CmoHC1 -----------------------------------------------------------VQDMVDYERRV
SamEHP -----------------------------------------------------------VQQMIDYTRRV
CacHC1 -----------------------------------------------------------IKDMIDYTRRI
PmaHC1 -----------------------------------------------------------IKDMLDYTRRI
ScuHC1 -----------------------------------------------------------VADLEAYEERI
CmaHC1 -----------------------------------------------------------IRDLKEMENRI
NviHEX109 ----------------------------------------------------RHHNYELIKLVRALERRI
NviHEX102 ----------------------------------------------------RHHNYELVKLARALEQRI
AmeHEX110 ----------------------------------------------------QRN--QLIQYVATLEKRL
AmeHEX70c -----------------------------------------------------YYKYKYLNVINALEMRL
CfeHEX2 -----------------------------------------------------FYKYKYLKEVEVLEKRI
NviHEX94 -----------------------------------------------------FYKYRYLEYLKMLETRI
NviHEX83 -----------------------------------------------------FYKYKYVEYVKQLETRI
AmeHEX70a -----------------------------------------------------IYKYKYIREIMNKESRI
NviHEX81 -----------------------------------------------------NYKFHKIHECEEYETRI
AmeHEX70b -----------------------------------------------------LHMQKYVQMIHDLHTRI
NviHEX79 -----------------------------------------------------VSKYKFLEEVKHYESRL
NviHEX75 -----------------------------------------------------SFKQQFLTLVKQYQDRL
TcaHEX1A -----------------------------------------------QKYGYN-KYAYSYTFVKDYERRI
TcaHEX1B -----------------------------------------------QHYGYN-KYAYSYTFVKDYERRI
TcaHEX2 -----------------------------------------------QHYGFN-KYAHSYTHISDYERRI
TmoHEX2 -----------------------------------------------QKYGFN-KYANSYSYVKDFERRI
TcaHEX3 -----------------------------------------------QKYGTN-KYAYSYTFVQDYERRI
TcaHEX4 -----------------------------------------------QKYGNN-RYAYSYTFVQDYERRI
TcaHEX5 -----------------------------------------------QSWTYKSAFGYSYSRVQDFERRI
RflHEXII ------------------------------NEYYSGNYFTGNYKPTYYYGYANNYDYYYPEDIKSYEGRI
RflHEXI -------------------------------------------------MLVTNFDTYTIEDIKNYERRV
LmiJHBS -------------------------------------------------LRPTHADVISLDDVISWERRV
HzeHEX --------------------------------------------------------RDVIRLCEEYERII
TniJHSP2 --------------------------------------------------------RDVIRLCDEYERII
HceHEX1 --------------------------------------------------------TELISLVEEYETLI
HceHEX2 --------------------------------------------------------LKLKMLLDDVEQMI
HviHEX ----------------------------------------------------------------------
BmoSP1 --------------------------------------------------------LKMKQMMDDVEMMI
CfuDAP2 --------------------------------------------------------LKYKLVFDDFEKMT
Dme7320 ------------------------------------------------------------KKISELEAKV
DmeFBP1 EILEMLQRNRQQRLQKHQNDDDDDVNDDDVNVVHRQGLRSRRSLPNLRQQNNRLSEIVLHNLRQLVARLN
Dme8100 -----------------------------------------------------------TELIYPPLMTT

Aga22058 MEAIDYGFIILPDG---------------TFYLGNLIQSNGDSVNKRFYGYLEKVAKFFLGGSFEMFNEF
Aga22055 REAIDLGYIVLPDGRHS------------IEYLGNLIQSNPDSVNSRFYKYVGWFARILLGGSIEHFENH
Aga9751 RDKVNLSTPESIDV------------------LGNLIQANPDSYEHRFYKYVAMYARIIMGAAIEPVEPY
DmeLSP2 MDWIHKNEKFDVETIN---------------VLGNIIQGNADSVDKKFYGSLDKLYRFIVNEGHHYGHGD
Aga29840 ----------------------------------------------------------------------
OatHEX1.2 RKMIDDGFYMTDDGTKID---LRKWS--NIDFLGQMMNCNMDNMKCQKFGFIEMMSKMLL--SGMDFMTQ
Aga16795 RQVIDKGYYYAQDGSKID---LRQPE--SVEYVGNMIFANPDSVDKDYFGFLEVVARNVY--SG-GAKFA
Aga4408 ----------------------------------------------------------------------
DmeLSP1b HNIVEMGYYKTADGHTID---LRKPE--AIEFIGNMLQGNVDAMDKMFYQFWYMLAHMYF--ADADYYQM
DmeLSP1a QNIVELGYYQTTDGHMID---LRKPE--SIEIIGNMLQGNVDAIDNIFFQFWYMLAHMYF--ADTHYYQM
DmeLSP1g YKVLETGFYKTADGQVFD---LHKPE--AIKIVGNYLQGNADTFDKYFFNYYYLLAHMYF--ADVDYNDM
Aga31208 ----------------------------------------------------------------------
BmoSP2 QFLQKDHFEAFGQKIDFH---DPKAINFVGNYWQDNADLYGEEVTKDYQRSYEVFARRVLGAAPMPFDKY
HmeHC1 REAISQMAVVTKSGE---YYSLNNTKGIN--TLGEIMEPSFDSKHPEYYGALHNYGHIMLGQIVDPKGKF
PamHC1 REAISQQAVVTRTGE---MVSLNNTQGID--ILGCMVEPSHDSKHPEYYGALHNYGHIMLGQITDPKGKF
CseHC1 REAISQQSVITRWGQ---NLPLNDTMGIN--ILGSLVEPSQESPNPQYYGALHNYGHILLGQITDPKGKF
CmoHC1 REAISQQAVITMSGD---YFSLNDTTGIN--TLGEMMEPSTTSKHRDYYGALHNYGHILLGKITDPKGKF
SamEHP RECISQQAVITETGD---LYNISSPEGIN--VLGELIEPSTGSKHREYYGALHNYGHIMLGQITDPKRKF
CacHC1 REAIAQQAVITKSGE---YFYLNDSKGIE--VLGDLMEPSFDSKHPEYYGALHNYGHILLGQITDPKGKF
PmaHC1 KEAISKQKVRSKNGE---KIPLDAVHGID--ILGDLMEPSVESPHEDYYGSLHNDAHVLLGQITDPLGKF
ScuHC1 REAISAGVIFDAHDPN-TFISLNNTEGID--KLGAIIEASSCSINPSFYGSLHNLGHIILGRVVDPLGKF
CmaHC1 RDAIAHGYADNTDGT---HLDINNDEGIN--VLGAAIESSTSSIHPSYYGALHNQAHRVLGQQADPHGKF
NviHEX109 VDAIDLGHVITPQGAFLS---LYQPEGLN--ILGQIIEGTGHSINPRYYGSYQAVAKQLLGNAPEFNNIY
NviHEX102 LDAIHLGHVITPQGAFLS---LYQPEGLN--ILGELIEGTGRSINPRYYGSFQYVARQLLGNAPEFNNIY
AmeHEX110 RDAIDSGNVITPQGVFLS---LYQPQGMN--ILGDLIEGTGRSVNPRYYGSLQAAARKLLGNAPEVENIW
AmeHEX70c MDAIDSGYLIDEYGKKID---IYTPEGLN--MLGNVIEGNSDSINTKFYGMYDILARDILGYNFDFQNKN
CfeHEX2 MDAIDLGYVHDKDGKQVN---IYTSEGLS--ILANLIEGNVDSCNRRFYGMYDALARDILGFNIDYKNKN
NviHEX94 MEAIDSGFIIDEMGKQVN---IMCPEGLN--ILANIIEGNYDSMNMKYYGSYDVLSRRVLGMNYDAKNKD
NviHEX83 LEAIDSGYFYDTMGKQIS---FYTPEGLN--YLGNIIEGNCDSFNIKYYGAYDLLARDILGMNFDCKCKD
AmeHEX70a SAAIDSGYILNNDGKWHN---IYSEKGLN--ILGNIIEGNADSYNTEFYGSIDTLARKILGYNLEAASKY
NviHEX81 SQAVDAGIIFSTDSKYAK---IYTPEGLN--ILGNMIEGNADSMNEQYYGNIDMLMRNILGFNIPPLTPE
AmeHEX70b STAIDLGYVVDSYGNHVK---LYTKQGLN--VLGNIVQGNGDSVNVQLYGQLDLLVRKVLGFGYESNVKY
NviHEX79 SKAIDLGELRTGSSFNKK---LDESSSFN--DLCNALQGNNDSPQPKLYGSLDRLYRRLLGFAPEVTGKK
NviHEX75 ITAADTGNLKGSNVP-------QGDEFLD--DLMNAAQGNADSPDQDYYGSYDVTARHILGFNPEPTDPF
TcaHEX1A RDAIDSGYVYTRGGDKVD---LFSHEGIN--ILGSLVEGNPDSPYYHYYGAYHVFARHLLGYSFQPLTYR
TcaHEX1B RDAIDSGYVFTRSGQKVD---LFSRDGIN--ILGNLIEGNPDSPYYKYYGAYQVFARHLLGYSFQPLTYH
TcaHEX2 HDVIDSGYVHTHSGQKVD---LFSHEGLD--ILGNLIEGNPESPYYHYYGAYQVFARHLLGYSHQPLTFH
TmoHEX2 HDVIDSGYAHTESGQTVE---LFSHHGMN--ILGNLIEGNPDSPYYHYYGAFQVFARHLLGYSQQPLTYF
TcaHEX3 REAIDQGYVYTPEGQKIN---LYTEQGLN--ILGNLIESNPDSPNYRYYGALQVYGRHLLGYSYQPLNQY
TcaHEX4 RDAIDRGYVFSHDGQRIN---LFSEDGVN--ILGNLIESNPDSPDRHFYGALHVYARHLLGYSSQPLDKY
TcaHEX5 SDTIDAGFAFTKSGKLVD---LYSSDQIN--VLGNMIESNPDSVNPRYYGPWDIYGRHLLGYSYQPLDKY
RflHEXII RDGIDFGYFFSEGGQPKYPLYDEYSKGIN--YLGDIIEGNGDTVNKRVYGAIYQAYRQLAGQSADPYNNY
RflHEXI ADVIDFGYFKDEHLKVHS--MYEDNNGIN--YLGQMIEGSYNSPHYYYYGSLFHFYRMMLGHMMDPFHKH
LmiJHBS RDAAATALFLNEKKLESLE----ESDAVN--RLASIVMGGPSSPAPNYYRSVSWGLQTLYGHIADPQHQY
HzeHEX REAIIKGFIEIN-GMRLELTKTEDMEVLGKLIYGKIDKLDLDRTVVDSYRYLLIVMKAALGLNTLHSDKY
TniJHSP2 RDAIIKGFIEIN-GMRLELTKTDDIETLGKLIFGKIDKVDLDKTLVDSYRYLLIVMKAALGLNTFHSDKY
HceHEX1 KEAIMKGYIDMN-GIRLELTKPEDIETLGKMIYGKVAAKEQDSKCVEAYRYLLIVMKSAIGLNTLESDKY
HceHEX2 REGILTGKIERRDGTMINLKKPEDVEMLARLILGGMNLANDDAKMFH----MMTMFRKMLSYNQYNMDKY
HviHEX ----------------------------------------------------------------------
BmoSP1 REGILTGKIERRDGTVISLKKSEDIENLARLVLGGLEIVGDDAKVIH----LTNLMKKMLSYGQYNMDKY
CfuDAP2 RHGYPHRRMETRDGTVITLKKAEDFEYLARLLVGGIGMLHDDAKVVH----ITNMLKKMFAYNVYNVDKY
Dme7320 GDTISFQSIQLTNGSRIN--------------------------LNENNNWLIGLEELFPYDWTQLAVDE
DmeFBP1 QESIAQGQLIEEQQQLINNPRLTQSERYALRLNQIRINSQRSRQVLAQIGQIEQRIQEVIGQVLNQVNVN
Dme8100 GGVPYKGTSLNIEAVRGIIDLTIDGLKDKIEMAVKHNNHAMDNLSIAGGIIGNNYLHICRQLNLAVNGQG

Aga22058 RAVPSVLERYETA----------------------------------------MRDPVFYQFFKRVIGFY
Aga22055 KVLPGVLEHYETS----------------------------------------LRDPMFYQLYKRIIQWY
Aga9751 QVIPSALEHYESA----------------------------------------MRDPVFYQIYKRIVAYY
DmeLSP2 ESFPGLFMHYDTS----------------------------------------MRDPIFYEVYKTIVSHY
Aga29840 ----------------------------------------------------------------------
OatHEX1.2 KTWPSVMMHHETT----------------------------------------LRDPMFYKLWDRLLNFY
Aga16795 KFFPSALMHFETS----------------------------------------MRDPFFYQLYNRFLTFY
Aga4408 -------------------------------------------------------DPIFYQLYDRFLDIY
DmeLSP1b DVYPNVMLNFETM----------------------------------------MRDPMYYMFYKSIAQVY
DmeLSP1a EVYPNVMLNFETM----------------------------------------MRDPMFYMFYKSIAQVY
DmeLSP1g EVFPNVFLNFETM----------------------------------------LRDPFFYTFYKKFTDVF
Aga31208 ----------------------------------------------------------------------
BmoSP2 TFMPSAMDFYQTS----------------------------------------LRDPAFYQLYNRIVEYI
HmeHC1 NMPPGVMEHFETA----------------------------------------TRDPAFFRLHKYIDNLF
PamHC1 NMPPGVMEHFETA----------------------------------------TRDPAFFRLHKYIDNLF
CseHC1 DMPPGVMEHFETA----------------------------------------TRDPAFFRLHKYIDNLF
CmoHC1 NMPPGVMEHFETA----------------------------------------TRDPAFFRLHKHIDNLF
SamEHP NMPPGVMEHFETA----------------------------------------TRDPAFFRLHKYIDNLF
CacHC1 NMPPGVMEHFETA----------------------------------------TRDPAFFRLHKYIDNLF
PmaHC1 DLPPGVMEHFETA----------------------------------------TRDPAFFRLHKHIDNLF
ScuHC1 GMPPGVMEHFETA----------------------------------------TRDPAFFRLHKHIDNIF
CmaHC1 NMPPGVMEHFETA----------------------------------------TRDPSFFRLHKYMDNIF
NviHEX109 EYSPSALELGQTA----------------------------------------VRDPIFYQLYSKIIELF
NviHEX102 EYSPSALELGQTA----------------------------------------VRDPIFYQLYSKIIELF
AmeHEX110 DYTPSSLELGEVA----------------------------------------VHDPVFYQLYKKVMNLY
AmeHEX70c NLIPSALQSYSTS----------------------------------------MRDPAFYMLYQKILSYF
CfeHEX2 KVVPSALQCYSTS----------------------------------------LRDPGFYRLTKRIMVYF
NviHEX94 MYIPSSLENFSTS----------------------------------------MKDPAFYRIYNKIVGFF
NviHEX83 YYVPSALQLFSTS----------------------------------------MRDPAFYRLYDRILFFF
AmeHEX70a QIVPSALEIFSTS----------------------------------------MKDPAFYRIYKRIIDYY
NviHEX81 KLQPSALEHYATS----------------------------------------MRDPAFWRMYKRIFHHY
AmeHEX70b QVVPSALQMWSTS----------------------------------------LRDPVFFSIYKTILDYY
NviHEX79 SGQVAALEMSWTS----------------------------------------LRDAGFFRMNKRILDLG
NviHEX75 HYIISALENLHAN----------------------------------------VRDPAFYRIYKSIVDIG
TcaHEX1A KVYPSALEHFETS----------------------------------------MRDPAFYQLYKKLITYF
TcaHEX1B KTYPSALEHFETS----------------------------------------MRDPAFYQLYKKLINYF
TcaHEX2 KLHPSALEHFETS----------------------------------------MRDPAFYQLYKKLLGFF
TmoHEX2 KLHPSALEHFETS----------------------------------------LRDPAFYQLYKKLLSYF
TcaHEX3 QVAPSALEHFETS----------------------------------------LRDPAFYQFYKRVLLYF
TcaHEX4 HVAPSALQHYETS----------------------------------------LRDPAFYQFYKRIVLYF
TcaHEX5 KVAPSALEHFETA----------------------------------------LRDPAFYQLYKKILMQF
RflHEXII GLAPSALQNIFTA----------------------------------------LRDPANYQILKRITYLF
RflHEXI GLAPSALEQPETA----------------------------------------LRDPAYYQLYKRMYHLV
LmiJHBS GMAPSALDMHLTM----------------------------------------YRDPLYYRIIKRIYGIF
HzeHEX FVVPSVLDQYQTA----------------------------------------LRDPVFYMLQKRILDLV
TniJHSP2 FVVPSILDQYQTA----------------------------------------LRDPVFYMLQKRIIDLV
HceHEX1 FVAPTVLDSYQTA----------------------------------------LRDPVFYQLQKRLVNIV
HceHEX2 TYVPTALDMYTTC----------------------------------------LRDPVFWKIMKRVMNSF
HviHEX ----------------------------------------------------------------------
BmoSP1 TYVPTSLDMYTTC----------------------------------------LRDPVFWMIMKRVCNIF
CfuDAP2 TYVPTAIDMYATA----------------------------------------LRDPLYWRLMKRVNEIV
Dme7320 VIETNILLDIRTI----------------------------------------VRSEDFYYYAERLLDSY
DmeFBP1 SLRGQVIDQRQVESLIADVLLGRLGQVGIMTIIRQVVQDNNIEQIDRTGLGIRLSDPVVQYTLRRIVRIV
Dme8100 INQPSMLGSATSN----------------------------------------LRDPIYRSLLFRIYDLL

Aga22058 YRYLD-TLPSYKYDEINFPGVKLETVEMDK-LVTYFDNFDADITNAVDVEVFDETTMKAGEMKKFGKMAH
Aga22055 WEFKD-HLQPYTKEELTFGELKFETVKVDK-LVTYFDRSDADITNAVDVEVFDESSMKAGEMKKFGKIAH
Aga9751 HQFKE-HLPEYTYEELYYPGVKIDGVVVDK-LQTYFDRFDVDITNAIDIEPEP---YVPGKYEGFGEIEY
DmeLSP2 WHLME-TYPEYHKKDYAFEGVHIDAVHMPESLTTYFEHFDSDISNAVNVEPAVEG--SADPLYTFGRNSH
Aga29840 ----------------------------------------------------------------------
OatHEX1.2 YLFKS-YLPYYTLEELNFKGVVIKDVVIDK-LMTYFEYFDSDISNVIPMMNV------------------
Aga16795 YQFKS-YLKPYTYEELYFKGVEIKSVVFDK-LLTYFEYYDSDVSNVIPLKASG-----------------
Aga4408 SYFKR-FLPSYTYEELNFNGVVIQDVTFDK-LMTYFDFFDSDVSNVLPMQST------------------
DmeLSP1b FQFMH-YLPKYTKEQLLMPGVTMKNVEVSD-LTTYFDLVDFDVTNMLNDKMIFQD--------------G
DmeLSP1a FQFMH-HLPKYTKEQLLMPGVTLKHVEVSE-LVTYFDLVDFDVTNMLNGKMVFHE--------------G
DmeLSP1g YTFKY-YLKPYTQKDLFYEGITIKDVSVSK-LVTYYDIVDFDVTNLLNDKMTFVD--------------G
Aga31208 ----------------------------------------------------------------------
BmoSP2 VEFKQ-YLKPYTQDKLYFDGVKITDVKVDK-LTTFFENFEFDASNSVYFSKEEIKN--------------
HmeHC1 KIHKD-LLPVYTHEELDFPGLKVVDLDVD-VLQTYLEDFDIDLLNALDDTQELD----------------
PamHC1 KLHKD-LLPHYTMEELGFNGVTIQDLTVD-DLVTYFEDFDIDMLNALDDTAELQ----------------
CseHC1 KLHKD-MLPPYAREELEFPGVAIQDVTVD-DLVTYFEDFDIDLLNALDDTLELK----------------
CmoHC1 KLHKD-LLPPYKQEELVLPGVKITDVAVE-PLETYFEDFDADLLNALDDTIDLD----------------
SamEHP YEHKD-LLPRYTSEELELPGTSIEEVQID-PLETFFEYFDVDLLNALDDTEELP----------------
CacHC1 KLHKD-LLPSYSKEELSLDGVQIEDVQID-ELTTYFEDFDIDLLNALDDTVELE----------------
PmaHC1 KMYKD-LLPPYTKAELEFPGVKVLDWEIG-NLVTYFEDFDIDMLNALDDTADLP----------------
ScuHC1 KEHKD-SLVPYTHEELDVEGVDIKNVEVD-DLVTYFEDFDIDMLNALDDAAGLT----------------
CmaHC1 KEQKD-KLPPYTTDDLKYDNVEITDVDID-ELSTFFEDFEFDLRNALDTSDNVG----------------
NviHEX109 HYYQE-ALPAYQYNDVVVPGVHIEKVQVG-DLVTYFSDYEVELDNAVPHPVEHH-------------HQH
NviHEX102 HYYQE-ALPAYQYNDVVVPGVNIEKVEVG-DLVTYFSDYEVDLNNAVVQPVG---------------QHQ
AmeHEX110 QQYQQ-SLPVYQYNDLILPGVTIQNVDVS-QLVTLFTDFYVDLDAVTGHQSQ---------------QQQ
AmeHEX70c LRYKK-LQPQYSQSELQMPGVKFESVNID-KLYTYFDKCDTLINNAVAVEN------------------F
CfeHEX2 FRYKK-NMPQYTQDELIFPGVKFESVNID-KLVTYFDNCDTVINNALSVES------------------F
NviHEX94 MKYKS-HLNRYTKNELEFSGVKIENVEID-KLYTYFDTREYMVNNLIDVAS------------------M
NviHEX83 HKYKS-HLTRYTKSELEFPGVRFEGVEVD-KLFTYFDKKEYFINNAVAVDS------------------F
AmeHEX70a HSYKM-HQKPYNKDEIIYPNLKIESFTVD-KLITYFEQFDTTINNGLLLEEQ-----------------R
NviHEX81 SKYNL-NQKSYKPEEIQYPELKIESVEVD-KLQTYFDYFDATISNGLAVES------------------Y
AmeHEX70b HKYKE-NLPKYTTEELNFPGVSIESVTVD-KLITYFDHFESMLNNGVSIQS------------------H
NviHEX79 LRFKR-NLPVYTPADLGFDGIRIDYVHLD-KLVTYLDSFESTLNHPRVHYFADDSA----------ARKE
NviHEX75 ISYKA-RLPSYTSEDIGFPGVEVDRVSVP-SLKTYFDEFDAILGYQKCS---------------------
TcaHEX1A FRYKAQHYKYYTEHDLAFEGVEVKNVEFD-RLVTYFDYYFADISSAVYVTPEE-----------------
TcaHEX1B YRFKSQYYKYYTEQDLVFEGVEVKNVEFD-RLVTYFDYYYSDISSAVYVTPEE-----------------
TcaHEX2 FRYKSQHYHYYDEHDLAYHGVHVKHVEVD-PLVTYFDYFYADLSNAVYVTPEE-----------------
TmoHEX2 IRYQANHKHYYNEHDLMYQGVEVKNVEFD-SLVTYFDYFYADLSNAVYVTPQE-----------------
TcaHEX3 QQYQN-NLPAYTAQDLSYQGVEVTNVQFD-RLVTYFDYFYTDLSNAVYVTPEE-----------------
TcaHEX4 QKYKS-YLPSYTEHDLHFQGVEVKSVEFD-RLVTYFDHFYTDISNAVYVTPQE-----------------
TcaHEX5 FKYKI-YLPPYTYNELNFPGVKIEKFEVD-KLFTYFDYFDSDVTNAVYT-PPN-----------------
RflHEXII QRYKN-YLPQYTYQELAYPGVTIENVEVG-KLITYNDYFDIDLDNVVNVKVPE-----------------
RflHEXI NKYKD-RLPRYTHEQLWFEGVTVENVDVG-KMYTYMENFEFSLGGTIYVAKEE-----------------
LmiJHBS EVYKN-NLPPYHAQDLTWGGVKIEELKVVDKLVTFFDDFDIRLDNAIDVGRVE-----------------
HzeHEX FLFKL-RLPCYTKEDLYFPGVKVDNVNVD-KLVTYFDDYLMDMTNAVFLTEEEMK---------------
TniJHSP2 HLFKL-RLPSYTKEDLYFPGVKIDNVVVD-KLVTYFDDYLMDMTNAVYLTEDEIK---------------
HceHEX1 QLFKK-RLPCYTKEELYFPGVKIENVVVD-KLLTYFDDYFMDMTNAVTLSEEEIK---------------
HceHEX2 EVFKN-MLPSYTREELDFPGIKVERIVTE-KMVTFMDEYDMDITNALYLDQAEMQ---------------
HviHEX ----------------------------------------------------------------------
BmoSP1 TVFKN-MLPKYTREQFSFPGVKVEKITTD-ELVTFVDEYDMDISNAMYLDATEMQ---------------
CfuDAP2 KVYKL-LLPKYTRDEFHFPGVKVESISTD-KLVTFMDEYDMDITNAVFLDDAEMQ---------------
Dme7320 RWYRQ-VFQPNNQKTFIPSDLRIDDMQIT-PLITYDQPVDVDISNILAAQHFYLAG--------------
DmeFBP1 DEQREQILGGYRQEQLQMRGVSINDVRVD-KLRTRIEEHELDLSNLVEQQVQG-----------------
Dme8100 KSYENENIFPDVGGRETPKIVDIQVSRLVTFEETMTTDLINLIDQQLLLSHRNN----------------

Aga22058 YQGEDFVLYARMPRLNHLPFSFKLNVVSDKPQKA-VVLVFLGPKYDQYGNAYSVNANR--ENFFQLDHFL
Aga22055 YQGEDFVIKARQWRLNHMPFTVEYSVVAEKPIKG-VVRMYLGPKYDQYGHAYGVNENR--ENFVLLDTFE
Aga9751 KP-DPVVIKARTMRLNHKPFTYKISLTADKPAKA-AVRVFLGPKYDEYGGLYTLNENR--ENFYELDYFV
DmeLSP2 YKGSSYVIKARQQRLNHKPFEFTLDVTSDKAQDA-VVKVFIGPKYDEHGHEIPLEHNY--QNFFELEHFK
Aga29840 DKYFDYAVFARQRRLNHKPFSYTMNVMSDYTGKA-IIRAFVGPKFDRF---FDLQFYK--KYFFEIDQYL
OatHEX1.2 DKHWDFTVLGRTMRLNHKPFTYTLDVMSEFSGKG-VVRVFLGPKFDKL---MDLDFYR--KFFVEVDQYQ
Aga16795 EKFFDFSVFARQKRINHKPFSYTMDVYSEFAGKG-VVRVYMGPKFYDF---KQLQYLK--KYFVEVDQYL
Aga4408 DKYFDYAVYARQSRLNHKPFSYTMNVMSNYTGKAAIIRAFVGPKFDRF---FDLQFYV--LLRD------
DmeLSP1b KFVWDMSLFARQMRLNHKPFTYTYTIESEKVEKV-VIRAFLGPKFDEFGKVISLAENR--MNFMEIDEFY
DmeLSP1a QFLWDKSLFARQMRLNHKPFSYTYTIDSARDEKV-VIRAFLGPKFDEYGRMISLTDNR--MNFMEIDEFT
DmeLSP1g QYIWDKALLARQARLNHKPFNFEFTIDSDKVQKG-VVRVFLGPKFDEYGRVIPLDYNR--KNFVQIDSFV
Aga31208 ----------------------------------------------------------------------
BmoSP2 ---NHVHELRCATRLNHSPFNVNIEVDSNVASDA-VVKMLLAPKYDDNGIPLTLEDNW--MKFFELDWFT
HmeHC1 ----DVEIKARVRRLNHQPFTFRVTVSSEKNAN-VAVRVFLGPKYDWFGVQIPLNEKR--LYMVEIDKFY
PamHC1 ----DVDIKARVRRLNHKPFSVQVTVNSERDAM-AVVRIFLAPKYDWFGQEIPINEKR--LYMIEIDKFV
CseHC1 ----DVEIKARVRRLNHRPFTFSITVNSNQEQM-AAVRIFLGPKFDWFGQEIPINEKR--LYVIELDKFV
CmoHC1 ----DVEIKARVRRLNHKPFTVQITAESERETL-ATVRIFLGPKYDWFGQEIPLEEKR--QYLVEIDKFV
SamEHP ----DVSIHARVRRLNHKPFVFSVRVNSEAERF-VTVRVFLGPKYDWFGQEIPINEKR--HYIVEIDKFV
CacHC1 ----DVEIKARVRRLNHKPFNFKIEVNSDKEYT-AAVRVYIGPKYDWFGQEITLDEKR--LYMVEIDKFV
PmaHC1 ----DVDVKARVQRLNHEPFTWALHMESDKEVT-AAFRVFLGPKKDWYESDFTINEVR--PYLIEIDKFV
ScuHC1 ----DVDIKARVQRLNHKPFGIKIIANSAAEKT-VTVRLFLAPKYDWYGREVPLDIQR--WKFIELDKFA
CmaHC1 ----DVTVKAHVSRLNHKPFYYNIHYNAKQDDK-VTVRVYLTPVRDENGIKLDINDNR--WSAIMIDTFW
NviHEX109 EHQPFPHVRAHLKRLDHKQYEYTIHVNAEKPVQGAVVRVYVGPKYNYD--GQPIDINVHRHYFFELDQFY
NviHEX102 EYQPFPKVKANLKRLDHKQYEYTIYVQAKKVVPGAVVRVYVGPKYNYD--GQPIDISQHRHYFYELDQFV
AmeHEX110 EEQTQSRVRAHLKRLDHQPYQYKIAVHSEQNVPGAVVRVFLGPKHDHQ--GRPISISKNQHLFVELDQFI
AmeHEX70c KGGMYLRLKARRACMNYERFTYKININSDKETK-GMMRIFLGPAFDEIK-HDMVYLQKYFYLFMEMDRFA
CfeHEX2 QEGMKLRVKARRYCLNHKPFTYRFTINSDKETK-AVLKIFLGPAFDDIRTKDLSHLRESYKYFFEMDHFE
NviHEX94 KDGFSFNMKAWQYHLNYKPFTYKFAVNSDKSTK-AIMRIFLGPAMEG--YDDYSFLLHYYQYFFMLDEFE
NviHEX83 KEGKSFSIKAWQYNLNYKPFTYKFAVNSDKDTK-AVMRVFLGPAVEGDKYDDYSYLLHYYQYFFMLDEFE
AmeHEX70a NDDKPFLIKIRQYRLNHKPFNFHITINADKPMK-AAIRIFIGPKYDS--HHKLIEIPEDLKYFYEIDNWM
NviHEX81 AEAEKYLIQVRQYRLNHKPFNVKINIKAEKPTK-AAIRIFLGPKYNV--HMKEIDFQEHYHEFYEMENFI
AmeHEX70b AKAKNTMIKARQYRLNHKPFTYHIVVNSDKNVK-GMVRIFLGPKYDEFG--HEVDLVHNYMNFMQMDEFV
NviHEX79 SCGPKTTVMVRQQRLNHKAFAYQIGVTSERVTK-GVVRIFLGPKVDA--RGNELDLEDSIEQFYELDRWI
NviHEX75 SNDEKTSVIVKQKRLNHEKFNVGVKVNSKVSTK-AVVRIFIGPKFDK--NDAELSLEQSQHKYFELDQFL
TcaHEX1A FVQDGFKVQVAQHRLNYKPFTYKIYVDSSVDTD-AVVKVFLGPKYDEYGRYINLTENW--MNFVQFDHFV
TcaHEX1B FVQDSFKVQVAQPRLNYKPFTYKIYVDSSIDTE-AVVKVFLGPKYDEYGRYINLTENW--MNFVQFDHFV
TcaHEX2 FVHDSFKVHVAQERLNHKPFTYKIYIDSDKDTE-AVVKVFLGPKYDEYGRYINLTENW--MNFVQFDHFV
TmoHEX2 FVQDSFKVQVAQQRLNHKPFTYKIYVNSDRETD-AVVKVFLGPKYDEYGRFINLTDNW--MNFVQFDHFV
TcaHEX3 FNGERVQIRARQYRLNHKAFTYKIYVKSSQAQK-ASVRVYLGPKYDEYGRYLNISQNR--LNFVQFDHFV
TcaHEX4 YDSEKVQVRVRQYRLNHKPFTYRVHVSSDKEQQ-AVVRIYLGPKYDEYGRYINISHNR--LNFVEVDHFK
TcaHEX5 FQWDNFKVKVRQQRLNHKPFTLKIYVNSDKVND-AVVRLFLGPKYDEYGRKLTMTEKR--LNFVELDTFR
RflHEXII DGQ-YVDYRARQTRLNHKPFTYSIDVTSDKATE-VYVRVFLGPKYDYLGREYNINDRR--HYFVEIDRFP
RflHEXI DML-GVNLHVRQPRLNHKPFTYKIEVSSEKAVD-AYVRVFLGPKHNYLDEEWDLNERR--HFFVEMDRFR
LmiJHBS DIR-KTNVVARQQRLNHKPFSYSLSVSSDKEQL-ALVRVFLGPADGAVP----VEDLR--HHFLVVDGFH
HzeHEX KTKSDMKFMVRKRRLNHQPFKVTLDILSDKAVD-CVVRIFLGPKKDHMDRLIDININR--LNFVELDTFL
TniJHSP2 KTKSDMVFMVRKRRLNHQPFKVTLDILSDKSVD-CVVRVFLGPKKDNLNRLIDINRNR--LNFVELDTFL
HceHEX1 KASASMGFLVRKRRLNHEPFKVSLEILSDKAVD-CVVRIFMGPKEDQLGRLIDINKNR--LNFVEMDSFV
HceHEX2 KKKSDMVYVARMRRLNHQPFKVSIDVMSDKAVD-AVVRIFIGPKYDCMGRLNSVNDKR--LDMVEIDSFV
HviHEX ----------------------------------------------------------------------
BmoSP1 NKTSDMTFMARMRRLNHHPFQVSIDVMSDKTVD-AVVRIFLGPKYDCMGRLMSVNDKR--LDMFELDSFM
CfuDAP2 KKKSDMLFVARQRRLNNHPFKVTVDVVSDKAVD-AVVRVFIGPKYDCMGRLMDINDKR--FDMVEIDSFL
Dme7320 QFVWPFTLQHRQFRLQHKDFSYNLLISSNKTQS-TIFRVFLTT--------SERGGNIQREPFYQLDSFL
DmeFBP1 ---IQQEIVGRQRRLNNKAFTIDMDITSDQDQD-AIIRIFLGPAEDQQGRQGASLDER-RRDFVLLDAIQ
Dme8100 LQFLRRNLVARQHRLNHDPFSIGLDIVAPENLVVEARMYLSLPNQLR--------------LRLHLDSFK

Aga22058 VDLVAGENA---ITRNSQDFSWFVKDRTTYFDLYKQVMQAYNGDYKFPLDMSE----AHCGFPARLMLPK
Aga22055 WEFKQGQNV---FVRESTQFPLYVQDRTPYFELYKWVMDAYNGKRQFPLDMTE----AHCGFPSRLMLPK
Aga9751 QELTAGKNV---LTRSSVNFNGYVKDRTSFYELYRSVMAGYTGAEKFQLDMSE----AHCGFPNRLMLPK
DmeLSP2 VHLEAGVNH---IKRASGDFSFWVNDRTTYLELYQKLMDATNSDYKFKLDQSE----AHCGVPNRMMLPR
Aga29840 VDFTAGKNT---FVRNSRDFYWSVKDRTMYTDLYKKIMLGYNGQEKFALDMSE----AHCGFPDRLILPK
OatHEX1.2 VDLMVGKNT---LVRNSRDFYWSVRDRTMYTDLYKKIMMSVAGKDKFVLDMSE----AHCGFPDRLILPK
Aga16795 YDFVTGKNT---IVRNSRDFYYSVRDRTTYTELYKKIMTAYNGGEKFVLDNSE----AHCGFPDRLLLPK
Aga4408 ----------------------------------------------------------------------
DmeLSP1b YELKAGTNK---ITRKSSEFYWTVKDRTTYTELYYYTMMAFDGKYDFPLDISE----PHCGFPDRLVLPM
DmeLSP1a YTLKTGSNL---ITRKSTDFAWTVKDRTTYTELYYYTMMAFDGKYDYPLDLTE----PHCGFPDRLVLPM
DmeLSP1g YPFIAGTNT---IKRSSKEFSWTAEDRITYTELYKYVMLASEGKYDFPLDISE----PHNAFPDRLVLPK
Aga31208 ----------------------------------------------------------------------
BmoSP2 TKLTAGQNK---IIRNSNEFVIFKEDSVPMTEIMKMLDEGKVP-----FDMSE----EFCYMPKRLMLPR
HmeHC1 TTVNKGTTV---IERKSSESSVTIPDRETTKVLQKKVEDAIQGNGAFLVNKDV----RHCGYPDRLLLPK
PamHC1 TKVNKGTTV---VQRKSSESSVTIPDRETTKVLTKRVEEALQGKTTYMVNKDV----RHCGYPDRLLLPK
CseHC1 AKVNKGATV---IQRKSSESSVAIPDRETTKILVRRVDDALQGKATYTVNKDV----RHCGYPERLLLPK
CmoHC1 TKVNSGKTT---IQRKSSESSVTIPDRETTKLLVAKVENAINGKATINVNKDV----RHCGYPDRLLLPK
SamEHP AKVNAGKTV---IARKSSESSVTIPDRETTKVLTQRVEDAIAGKVQLTVNKDV----RHCGYPDRLLLPK
CacHC1 TKLTAGHNN---IFRKSSESSVTIPDRETTKVLHKKVKDALKNSTPLLVNKDV----RHCGYPGRLLLPK
PmaHC1 TKVVAGKSV---IHRKSSESSVTIPDRETTKVLLEKVEHALEGKETLNVNKDE----RHCGYPDRLLLPK
ScuHC1 VKLTAGENA---IVRKSSESSVTIPDPVSTHDLRKLVDDAIAGTATLEVDKDV----RHCGVPDRLLLPK
CmaHC1 TEVKAGTHN---HRRSGFESNVAIPDRISFEELIKETDEAVDNNLELSLNSG-----RSCGHPQRLLLPK
NviHEX109 YDIVEGHNA---IVRNSHQATGQSYDWPSVQQIRERVEGAIKSQNPYYITYP----QQLFGFPARLSLPK
NviHEX102 YDITEGQNV---ITRNSHQFAGQSYDYPSVQQLKESIKGAYSSQSPYYINYP----EQLYGFPARLTLPK
AmeHEX110 QNLHAGENT---IIRNSQQAPGQSPDWPSTSQIQRGVNAAIRSQEPFYITEP----HQIFSFPARLSLPK
AmeHEX70c VTLRPGSNS---IERQSSESPFTTSTIMPSDIFYDKLNKAIGGSEPFTYS------EKMLGFPERLILPR
CfeHEX2 VTLKQGTNT---IERHSSDSVFTMPDLVSSDTFYKQLERAMSGSAPFTYV------EKFFTLPERLVLPK
NviHEX94 FNLHEGMNN---FERKSHDVDGPQHEYMTGDRFYKKMMDAMHSKEGMHFN------KQMWSFPKYMTLPK
NviHEX83 VNLKSGMNS---FDRLSTDSFFFKGEYMTGDQFYKKVLNAIEGHAPFQYD------NRMFGFPNHMMLPK
AmeHEX70a LDLNSGLNK---ITRNSLDCFFTMNDLEPSEIFYEKIETSLNSDKPFTYN------ERIFGFPGRLLLPR
NviHEX81 YDLAAGENK---IQRNCHDFFFVDHEYEPSEVFYKKLSKAIEGAEELKYE------KRLMGFPDRLLLPK
AmeHEX70b VNLKSGSNT---IERNSHESVFVVPDEVPSDVLYNRLVVSEDGSETFKYS------SQPYGFPERLLLPK
NviHEX79 VDLKQGSNK---LDRSSNDSPYYSSDPQSSEAYNRDIVRALRTTDENLARDIVHSRKLHLGLPRRLLLPR
NviHEX75 VNLEKGDNY---ITRRDSEFAFQRPDDLGSDEFYDLIKASLANENAGLL-----VRQRPYGWSRRLIIPR
TcaHEX1A YKLKSGQNV---ITRNSREIFNYIHDRTSYYQLYQKAMGVSDDEQRGYYHHTQF----WFGFPQRFYLPK
TcaHEX1B YKLKSGQNV---ITRNSREIYNYMHDRTSYYQLYQKAMGVFDEKQGEYYKYTQF----WFGFPQRYMLPK
TcaHEX2 YKLKSGENV---ISRNSHEIYNYIHDRTSYYELYQKAFGVYNKDQHFQFHDNQF----FFGFPQRYMLPR
TmoHEX2 YKLKAGDNI---ISRDSHEIYNYMHDRTSYYELYQKAFGVHDQSHSFQFHHDQF----WYGYPQRYMLPM
TcaHEX3 YQLQAGENT---IERNSRQSYFYQNDRTSYQELYQKVLGALDGNGQFSVEPNEA----YFGFPRRFLLPK
TcaHEX4 YQLKVGENV---IERNSHQNYFYQNDRTSYRQLYKQVLGALNGNGEFNVNANEA----YFGFPRRFLLPK
TcaHEX5 YKLQSGENV---IERTSRDFYWYVPDKTSYRDLYKKILGALDNTESLQLDSSEA----FYGFPNRLLLPK
RflHEXII HKIQEGKTT---IKRNSRDSSVVTPDYPSYRTLLRKVSDALEGKEQFYIDRSER----YCGYPERLLLPR
RflHEXI HHVPAGKSV---IERNSHDSSIIAPTPDSYRTFVKKVQDAYDGKTQYFIDKSHN----YCGFPENLLLPK
LmiJHBS TKLKPGNNT---IVRKSREMLSVSDDPSTFRELYGRVEAALGGGERLVAGAERVR---LEGYPHRLLLPR
HzeHEX FKLTTGKNT---IVRNSHDMHNIVHDRMFTRDLMKKVESITDMRDLLIKDLRNYHT----GFPTRLLLPR
TniJHSP2 YKLNTGKNT---IVRNSYDMHNLVKDRMMTRDFMKKVESITDMRDLMIKDLRNYHT----GFPTRLLLPK
HceHEX1 YKLTTGKNT---IVRKSSDMHNLVPDRMMTSDLWKKVESITDMRDLFVKDLKNYFT----GFPTRLLLPK
HceHEX2 YKLDTGKNN---IVRSSLEMHGVIEQRPWTKNILEKGFDTTGSG---FKSIESWWYKSRLGFPHRLLLPL
HviHEX --GTSGKNT---IVRNSLEMHGVIEQRPWTRRILNNMVDTVGMISK-TVDVESWWFKTRVGFPHRLLLPL
BmoSP1 YKLVNGKNT---IVRSSMDMQGFIPEYLSTRRVMESEMMPSGDG---QTMVKDWWCKSRNGFPQRLMLPL
CfuDAP2 YKLETGKNTNSVGATREIEMHGVIEDRPWTSRVWDHSFDAVDTSSHDRVMDDSWWYKTRTGFPTRLLLPM
Dme7320 TVIYPGLNR---ITRESKEFKGLAGDHISYTELYQFVKLAERDEFDFPLNISTAN----CAFPRRLILPR
DmeFBP1 VQLENGRNR---IHRRSIDIPWTTSDVTPLVEIYRQVMLQLKGQQAQQVVGIQQLVGENGRFPQHLLLPR
Dme8100 CSLKRGLNH----------FERQFPTAISKPTLSELYEADYASTDVTTSN----------RFPPHLQLPR

Aga22058 GKKGGMPFQFFFMIAPYHAPEVERFTGYDQTLSCG-----------------------------------
Aga22055 GKKGGMPFQLYFIVSPYHAPAVPQHEGYDYTLNCG-----------------------------------
Aga9751 GKKGGMPFQLYVIVSPYKAPQVSQYSGFDPVLSCG-----------------------------------
DmeLSP2 GKKGGQVFQFFYMVYPYHQPEVAQFTGYDPVVSCG-----------------------------------
Aga29840 GWTSGMPMQFYFIITPYTAKTYEQGYQYDKTFTCG-----------------------------------
OatHEX1.2 GWVSGMKMQFYFIITPYTMTQTATDVIFDKTFMCG-----------------------------------
Aga16795 GLPSGYEMTFYFIVTPYYAPKVQQFSTYDYTYSCG-----------------------------------
Aga4408 ----------------------------------------------------------------------
DmeLSP1b GWQKGMPMQMFFMVVPYVAPAHEQFSTFDYTYSCG-----------------------------------
DmeLSP1a GWKKGMPMQMFFMVVPYMAPQHEQFSTFDYTYSCG-----------------------------------
DmeLSP1g GWEQGMPMQFYFFVSP-FAETYEQFSNFDYTYSSG-----------------------------------
Aga31208 ----------------------------------------------------------------------
BmoSP2 GTEGGFPFQLFVFVYPFDNKGKDLAPFESFVLDNN-----------------------------------
HmeHC1 GKKDGMPFTFYVIVTDFDKEKVNDVPLDYNYGGSISYCG-------------------------------
PamHC1 GKKDGMIFTMYVIVTDYETEKVNDLPYDYEYGGAISYCG-------------------------------
CseHC1 GKRDGMPFSLFVILTDFDKEKVNDLPWDYDYGGSISYCG-------------------------------
CmoHC1 GKKGGMPFTLYVILTDFNKEKVNDLPYDYDYGGSLSYCG-------------------------------
SamEHP GRRDGMPFTLFVVLTDYEKDKVNDLPFDYDYGGSVSYCG-------------------------------
CacHC1 GKIEGMPFPLYAIVTDFEQEKVNDLPFDYDYGGSISYCG-------------------------------
PmaHC1 GRNTGMPVQIYVIVTDFEKEKVNDLPYDYDYGGSLSYCG-------------------------------
ScuHC1 GKTNGMKYTMFVMLSDFEEDKVNDLPHDYEYGGFVSYCG-------------------------------
CmaHC1 GNEEGLQFWLNVYVTSGEDAVHDDLHTDIHG--NHGYCG-------------------------------
NviHEX109 GTKSGFPLQFFVIITAGIHHQPEHYGPVVEENWMTYQPHHYQIVSSEDYEQFAQHPVDKIHGGYQSVDVI
NviHEX102 GSQSGFPLQFFFIITAGTQHQPEHYGPVVEEQWQTYQPYHYQIVGDEEYKQFTQNPVDTIHGGYQTIEVI
AmeHEX110 GQPQGFPLQFLVVISSSNASGQQHAG------------------------------------GWQSIYAQ
AmeHEX70c GKPEGMRYKMFFFLSSMDESNTKSYEIP------------------------------------------
CfeHEX2 GKPEGMRFKMFFYLSTLDGSKVRNVELP------------------------------------------
NviHEX94 GSVDGMRFKLFFYISSFEE--GKAMELP------------------------------------------
NviHEX83 GKVDGMRFKLFFYLGPYQE--VKSFELP------------------------------------------
AmeHEX70a GKKEGMPFQLFLYVSPVSS-EYNQYNSR------------------------------------------
NviHEX81 GKPEGMPFQMFIFVSPIQG-EPMAYTSR------------------------------------------
AmeHEX70b GKKEGMPYNVLVVVSPFDDSNVVQIDSP------------------------------------------
NviHEX79 GNRSGLGLRLVVCLHGLDGAKVDKLDEET-----------------------------------------
NviHEX75 GKPDGLTLRLFVIVNKFD-EDKAVHTDSL-----------------------------------------
TcaHEX1A GTYGGFPYQFYVYVTKYVQYKNQKSE--------------------------------------------
TcaHEX1B GTYGGFPYQFYVYVTKYVPYKAQKDG--------------------------------------------
TcaHEX2 GSPEGMTYQFYVFVTKYHPYKAHAS---------------------------------------------
TmoHEX2 GSRGGMTYQFYVMVSKQLPYKTHTT---------------------------------------------
TcaHEX3 GNYGGYEYQFYVIVSPYVPYQGQQT---------------------------------------------
TcaHEX4 GNYGGQEFQFFVIVSPYVPYKYHQEGYD------------------------------------------
TcaHEX5 GKREGQVFQFYVIINPYQAPGRQEVQ--------------------------------------------
RflHEXII GKKGGQSFTFYVILTPYVQQGEHEFEPYN-----------------------------------------
RflHEXI GQKGGETFTFYVIITPYVKQDEHDFEPYD-----------------------------------------
LmiJHBS GRPSGLPLELVAVATDAREP--------------------------------------------------
HzeHEX GFVGGMDCMLYVIVTPLRLVDNVDMN-VLDIY--------------------------------------
TniJHSP2 GFVGGMHMMLYVIVTPLRLVDNVDIN-ILDIN--------------------------------------
HceHEX1 GSVSGLPMVMYVMVSPLKLVDNVDMSAMLDPT--------------------------------------
HceHEX2 GTLGGFPLQMFVIVTPVKTG---MVLPSIDMS--------------------------------------
HviHEX GRRGGMPLQMFVIVSPVKTN---LLLPTVDMT--------------------------------------
BmoSP1 GTIGGLEMQMYVIVSPVRTG---MLLPTLDMT--------------------------------------
CfuDAP2 GSRGGLEFQMFVIVSPVRTG---LTLPTIDMA--------------------------------------
Dme7320 GGLGNPLKLRLLIVASVYDFRARQENELN-----------------------------------------
DmeFBP1 GRPEGLPMQLLVVVSPLVELQVQDVVPAITIG--------------------------------------
Dme8100 GTGDGLKLQLLVELSEWN----------------------------------------------------

Aga22058 ----------------------------------------------------------------------
Aga22055 ----------------------------------------------------------------------
Aga9751 ----------------------------------------------------------------------
DmeLSP2 ----------------------------------------------------------------------
Aga29840 ----------------------------------------------------------------------
OatHEX1.2 ----------------------------------------------------------------------
Aga16795 ----------------------------------------------------------------------
Aga4408 ----------------------------------------------------------------------
DmeLSP1b ----------------------------------------------------------------------
DmeLSP1a ----------------------------------------------------------------------
DmeLSP1g ----------------------------------------------------------------------
Aga31208 ----------------------------------------------------------------------
BmoSP2 ----------------------------------------------------------------------
HmeHC1 ----------------------------------------------------------------------
PamHC1 ----------------------------------------------------------------------
CseHC1 ----------------------------------------------------------------------
CmoHC1 ----------------------------------------------------------------------
SamEHP ----------------------------------------------------------------------
CacHC1 ----------------------------------------------------------------------
PmaHC1 ----------------------------------------------------------------------
ScuHC1 ----------------------------------------------------------------------
CmaHC1 ----------------------------------------------------------------------
NviHEX109 PDFDNHVITEGNWHWGYLYKKYPGSYYYPHWQQTHYSYGGEHHYEHHYPEHHEGQYHGEQHVEQHGEHHG
NviHEX102 PDFDSQIITEGNWHWGYLYKKYPGSYYYPHWQQTYYSQKGGDSHHYGSHYKTDYPKHGATYHYGQGGYHG
AmeHEX110 PQTVQDQIVS------------------------------------------------------------
AmeHEX70c ----------------------------------------------------------------------
CfeHEX2 ----------------------------------------------------------------------
NviHEX94 ----------------------------------------------------------------------
NviHEX83 ----------------------------------------------------------------------
AmeHEX70a ----------------------------------------------------------------------
NviHEX81 ----------------------------------------------------------------------
AmeHEX70b ----------------------------------------------------------------------
NviHEX79 ----------------------------------------------------------------------
NviHEX75 ----------------------------------------------------------------------
TcaHEX1A ----------------------------------------------------------------------
TcaHEX1B ----------------------------------------------------------------------
TcaHEX2 ----------------------------------------------------------------------
TmoHEX2 ----------------------------------------------------------------------
TcaHEX3 ----------------------------------------------------------------------
TcaHEX4 ----------------------------------------------------------------------
TcaHEX5 ----------------------------------------------------------------------
RflHEXII ----------------------------------------------------------------------
RflHEXI ----------------------------------------------------------------------
LmiJHBS ----------------------------------------------------------------------
HzeHEX ----------------------------------------------------------------------
TniJHSP2 ----------------------------------------------------------------------
HceHEX1 ----------------------------------------------------------------------
HceHEX2 ----------------------------------------------------------------------
HviHEX ----------------------------------------------------------------------
BmoSP1 ----------------------------------------------------------------------
CfuDAP2 ----------------------------------------------------------------------
Dme7320 ----------------------------------------------------------------------
DmeFBP1 ----------------------------------------------------------------------
Dme8100 ----------------------------------------------------------------------

Aga22058 ----------------------------------------------------------------------
Aga22055 ----------------------------------------------------------------------
Aga9751 ----------------------------------------------------------------------
DmeLSP2 ----------------------------------------------------------------------
Aga29840 ----------------------------------------------------------------------
OatHEX1.2 ----------------------------------------------------------------------
Aga16795 ----------------------------------------------------------------------
Aga4408 ----------------------------------------------------------------------
DmeLSP1b ----------------------------------------------------------------------
DmeLSP1a ----------------------------------------------------------------------
DmeLSP1g ----------------------------------------------------------------------
Aga31208 ----------------------------------------------------------------------
BmoSP2 ----------------------------------------------------------------------
HmeHC1 ----------------------------------------------------------------------
PamHC1 ----------------------------------------------------------------------
CseHC1 ----------------------------------------------------------------------
CmoHC1 ----------------------------------------------------------------------
SamEHP ----------------------------------------------------------------------
CacHC1 ----------------------------------------------------------------------
PmaHC1 ----------------------------------------------------------------------
ScuHC1 ----------------------------------------------------------------------
CmaHC1 ----------------------------------------------------------------------
NviHEX109 EQYHGKHHHAGEQYHGEQYHGQQYHGVHHGYAGEQYHGKTPYHGVEQGKTYHGEQYHGTAHHGHGFEHEH
NviHEX102 QEGYHGQYPYAYQKGTEQPEGGKYQTYYK---------------------------------------QG
AmeHEX110 ----------------------------------------------------------------------
AmeHEX70c ----------------------------------------------------------------------
CfeHEX2 ----------------------------------------------------------------------
NviHEX94 ----------------------------------------------------------------------
NviHEX83 ----------------------------------------------------------------------
AmeHEX70a ----------------------------------------------------------------------
NviHEX81 ----------------------------------------------------------------------
AmeHEX70b ----------------------------------------------------------------------
NviHEX79 ----------------------------------------------------------------------
NviHEX75 ----------------------------------------------------------------------
TcaHEX1A ----------------------------------------------------------------------
TcaHEX1B ----------------------------------------------------------------------
TcaHEX2 ----------------------------------------------------------------------
TmoHEX2 ----------------------------------------------------------------------
TcaHEX3 ----------------------------------------------------------------------
TcaHEX4 ----------------------------------------------------------------------
TcaHEX5 ----------------------------------------------------------------------
RflHEXII ----------------------------------------------------------------------
RflHEXI ----------------------------------------------------------------------
LmiJHBS ----------------------------------------------------------------------
HzeHEX ----------------------------------------------------------------------
TniJHSP2 ----------------------------------------------------------------------
HceHEX1 ----------------------------------------------------------------------
HceHEX2 ----------------------------------------------------------------------
HviHEX ----------------------------------------------------------------------
BmoSP1 ----------------------------------------------------------------------
CfuDAP2 ----------------------------------------------------------------------
Dme7320 ----------------------------------------------------------------------
DmeFBP1 ----------------------------------------------------------------------
Dme8100 ----------------------------------------------------------------------

Aga22058 --------------------------------------------VGSGARYIDALPFGYPFNRKINEAAW
Aga22055 --------------------------------------------VASGARYIDSLPFGYPFDRPIDEKVW
Aga9751 --------------------------------------------VGSGARYMDSYAFGYPFDRPIDEKLF
DmeLSP2 --------------------------------------------VGHGSRYVDALPFGFPFNRPVKHDYY
Aga29840 --------------------------------------------VESGMRFYDSLPFGYPFDRVINFNYF
OatHEX1.2 --------------------------------------------KYN---FIDNLPMGFPFDREIDYTYW
Aga16795 --------------------------------------------VGSGSKYIDDLPFGYPFDRDIDFSYF
Aga4408 ----------------------------------------------------------------------
DmeLSP1b --------------------------------------------IGSGARYVDSLPFGYPFDRAIDEYEF
DmeLSP1a --------------------------------------------IGSGARHVDSLPFGYPFDREINEYEF
DmeLSP1g --------------------------------------------VGSGTRFVDTKPFGYPFDRQIDESDF
Aga31208 ----------------------------------------------------------------------
BmoSP2 --------------------------------------------LLASLWIAPLLMHYSRFLTCISRIFS
HmeHC1 --------------------------------------------TLAGHKYPDNKPMGFPFDR-RIDEDH
PamHC1 --------------------------------------------TLAGHKYPVNKPMGFPFDR-QIDGDN
CseHC1 --------------------------------------------TVSGHKYPDSKPMGFPFDR-QINSDN
CmoHC1 --------------------------------------------TIN-HKYPDSRPMGFPFDR-RLSTEE
SamEHP --------------------------------------------TLG-HRYPDAKPMGFPFDR-RIDQDS
CacHC1 --------------------------------------------TLG-HKYPDSKPMGYPFDR-PIG-RE
PmaHC1 --------------------------------------------VVGGHKYPDTKAMGFPFDRRIYSRED
ScuHC1 --------------------------------------------TIN-HKYPDKKPMGWPLDRKICKDSF
CmaHC1 --------------------------------------------IQG-AKYPDKRPMGFPFERRVPDIRV
NviHEX109 EYYGSGHYPGASEYHGTYPTGTYKGYQGHEEQLQQFYNNEFIGDIIGGAISLDGKPLGYPFDRQLAHSAF
NviHEX102 AYHGSGYYPGG---------HYSQSHKGQSEYVHNYYQNKYIGDVIGGAVSFDNKPFGYPFDRPIADSAF
AmeHEX110 ----------------------------------EYYQNKPISEVIGGAISLDGKPLGFPLDRPLSLGAL
AmeHEX70c ---------------------------------------------LYGKMTLDDKVFGFPLDRPMWAWNF
CfeHEX2 ---------------------------------------------IFGKLTLDEKPLDFPLDKPMHPWKF
NviHEX94 ---------------------------------------------IFGQRMYYGKPFNFPIDRPMYPWFF
NviHEX83 ---------------------------------------------IFGNFKYYGKSFGFPLDRPMFPWFF
AmeHEX70a ---------------------------------------------IWGGYKFDKRSFGFPLDKPLYDFNY
NviHEX81 ---------------------------------------------VFGDSLMDNRPAGYPLNRPIQKFDF
AmeHEX70b ---------------------------------------------VWGRHIYDGRAMGFPLDKPVDPLLL
NviHEX79 ---------------------------------------------LFGGLALDGRDLGFPLDRPIGGEGF
NviHEX75 ---------------------------------------------IAGKGLLDSRSLGFPFDRSVDHLKF
TcaHEX1A -----------------------------------------VPVVGSGYQYVDGYPMGYPFDRPVYYGQV
TcaHEX1B -----------------------------------------VPMVGMGSQYVDGYPMGYPFDRPVYYGYM
TcaHEX2 -----------------------------------------VPMVGSGMHYVDAYPMGYPFDRPVYYEEL
TmoHEX2 -----------------------------------------VPMIGSGMQYVDSYPMGYPFDRPVYWEQV
TcaHEX3 ------------------------------------DSKYYYPRVGSGAQYLDNYPLGYPFDRPVHYDQV
TcaHEX4 ------------------------------------ASKYYYPRVGSGAHHIDNYAFGYPFDRPIHYDQI
TcaHEX5 -------------------------------------QDYYFHRVGTGMNYIDNYAFGFPFDR--TIVSY
RflHEXII ------------------------------------YKSFSYCGVGFNNKYPDYKPLGYPFDRPIYGSDF
RflHEXI ------------------------------------YKAFSXCGVGQDRKYPDDMPLGFPFDRQIHSKDF
LmiJHBS --------------------------------------------AYPGQVFSDGRDPFFPFDRRLFLWEL
HzeHEX --------------------------------------RKDLVRDFRSTVLLDKMPLGFPFDRRIDVGNF
TniJHSP2 --------------------------------------RKDLMRDFRSTVLLDKMPLGFPFDRRIDVGNF
HceHEX1 --------------------------------------SKALSFDFRSTVLMDKMPLGFPLDRYIDVTNF
HceHEX2 --------------------------------------TMKARYACRWSVCFDTMPLGFPFDREIYMPTF
HviHEX --------------------------------------IMKERKTCRWSVCFDTMPLGFPFDRKIDMTHF
BmoSP1 --------------------------------------MMKDRCACRWSSCISTMPLGYPFDRPIDMASF
CfuDAP2 --------------------------------------QMKDRHTCFWTTCVDSMPLGFPFDRVIETTHF
Dme7320 ------------------------------------------CDFSQGVSRWDELPLGYPFERFLEDDAL
DmeFBP1 ---------------------------------------------IGSASLRDARPLGYPLDRPIHNEQE
Dme8100 ---------------------------------------------------------GLESHFDGASVTV

Aga22058 FTP----NMVYYDTMIFHKSETEVNSVVV-----------------------------------------
Aga22055 FTP----NMYYLDTMIFHKKEAEINAVH------------------------------------------
Aga9751 YAVP---NAFFQDVSIYHKSEGEL----------------------------------------------
DmeLSP2 FDVH---NFKFVDVKIFHRDEHTNVV--------------------------------------------
Aga29840 YTK----NMYFKDVFIFHTEEMKMNQTY------------------------------------------
OatHEX1.2 YTK----NMLFKDVFIYHMDDVKMNQTF------------------------------------------
Aga16795 YTK----NMYFKDVLIFHSDEFKANLSY------------------------------------------
Aga4408 ----------------------------------------------------------------------
DmeLSP1b FVP----NMYFKDVSIYHADTMEPYYKYKSYSNYGHFDYTFFNDYYTKYFKF------------------
DmeLSP1a HVP----NMYFKDVTIYHADTMEKYYNYKEYTNYGHFDYSFFNDYYTKYFKL------------------
DmeLSP1g FVP----NGFFKDVKVYYVDTFAKYFEKK-YTQFGTFDYSIEY---------------------------
Aga31208 ----------------------------------------------------------------------
BmoSP2 FTTR---VNGSLTNSIFLRMTHMIMLFQKIKF--------------------------------------
HmeHC1 FFTPN---MGQKEVTISFKEVP------------------------------------------------
PamHC1 FCTPN---MFQKDVIITFKDTI------------------------------------------------
CseHC1 FFRSN---IYQKDVVITFKDSE------------------------------------------------
CmoHC1 FSMPN---FCSKDVTITFKGDSS-----------------------------------------------
SamEHP FFTKN---IYQRDVTITFKGSV------------------------------------------------
CacHC1 FYYPN---MFEKDVVITHKES-------------------------------------------------
PmaHC1 FFTDN---MYTKDVTITFKENHHH----------------------------------------------
ScuHC1 HDVTN---MYFRDVEIKFEHTH------------------------------------------------
CmaHC1 IKNLP---NFFGKIVDVYHKDH------------------------------------------------
NviHEX109 YAHN----IYLKDVVVYHHDEYFTEY--------------------------------------------
NviHEX102 YAHN----IYIQDVYVYHHNEYIPEY--------------------------------------------
AmeHEX110 SVPN----IFVKDVLVFHQGQPTNDITQ------------------------------------------
AmeHEX70c TIPN----MYFKDVFIYNRP----------------------------------NEESMNY---------
CfeHEX2 FTPN----MLMKDVYIYHIPGNTINENRMTDDTMIENWMNENWLNEDRVNEDRLNEDTMNKSWLKEHRMN
NviHEX94 SLGN----VFFKDVFIYHQPEQEMIGYHNMNTMMHNSMQDSMYTSKPNMMHDSMQDYGKVMRMNRDRYMK
NviHEX83 KLDN----CYFKDIFIYHMKDYDVKFSHQY----------------------------------------
AmeHEX70a EGPN----MLFKDILIYHKDEFDMNITY------------------------------------------
NviHEX81 HGPN----FFFKDVLIYHKQEHDLNIMF------------------------------------------
AmeHEX70b VLSN----IHVKEVLVHHREMEELNVAL------------------------------------------
NviHEX79 ERLRN---FMVKDIKVFHEDVKDVEKD-------------------------------------------
NviHEX75 EGTN----FAFKDIVVYHKH--------------------------------------------------
TcaHEX1A FYDIP--NGYFYETKVYHRDADAVNYSQQEYYNKQNYYQHHQHQYKPQHFNKQYNYAKQYYP--------
TcaHEX1B YYAIP--NGYFYETKIFHRDSDVFNYSQQDYYYGKRYNFANQRYQSKKHDYKYLSGSKDYVRPQYETQGR
TcaHEX2 FYALP--NSYFQDVRIYYQGHNYEHHANHLIEM-------------------------------------
TmoHEX2 FYDIP--NSYFHEVKIYHEHHDHGYEQHLEHEATHYMV--------------------------------
TcaHEX3 YNNIP--NSFVYSAKIYHRDVEDINASSASSQ--------------------------------------
TcaHEX4 FHNVP--NAYFYTTKIYHRHADDINASTSAHQ--------------------------------------
TcaHEX5 DFKVP--NSKFQDVTIYHKSSDDLNSSQAQNEP-------------------------------------
RflHEXII YTTN----SYFKDVVIFHKKEEEVNTAITQ----------------------------------------
RflHEXI YTHQ----HVLQGCTIFPQETRRSQYSHPLGQQCEISTSHLLSGNGFSISKPKATDILQSDVLTLVNIIL
LmiJHBS RSAP---NARFEPVTIYHRVSKDAKH--------------------------------------------
HzeHEX FTPN----MKFIDVKIFHKKMTCDMKTRWNRWVLRDYNMVDRTTIDSDTYFVDTDLN--MKMDRNINLTD
TniJHSP2 FTPN----MKFVEVTIFHKRMTCDMKTRWNRWVLKDYDMVDRTRIESDSYFVDTDLD--MKVNRNVNLID
HceHEX1 YTSN----MKFVDVTIFHKNQSCDMKMRWDKWVLKSYNLASKTPIESNTYFVDTDLNSSSKVESSANVYD
HceHEX2 FTNN----MKFTDVWVYRKDLSTMSNTTKDVDLSDMVMKKDDLTYLDSDMLMNWSYKDVMLMSSDNMLRM
HviHEX FTNN----MKFTDVLVYRKDMG-MSNSVKDIDTSDMVMKKDDLTYLDNDQLVRWSYKDVMMMSKDDMMRM
BmoSP1 FTSN----MKFADVMIYRKDLG-MSNTSKTVDTSEMVMMKDDLTYLDSDMLVKRTYKDVMMMSSMMN---
CfuDAP2 FTPN----MKFTDILVFRKDMD-LANVNKEVDTSDMVMRRDDLTYLDKDMLMNWSYRDVMLMSTDKMMRM
Dme7320 AVEISGDHVFWKDVHILHEN--------------------------------------------------
DmeFBP1 LLQLTN--VLLQDVVIIQEN--------------------------------------------------
Dme8100 LAKT------LKDVIIFHNQA-------------------------------------------------

Aga22058 --------------------------------------
Aga22055 --------------------------------------
Aga9751 --------------------------------------
DmeLSP2 --------------------------------------
Aga29840 --------------------------------------
OatHEX1.2 --------------------------------------
Aga16795 --------------------------------------
Aga4408 --------------------------------------
DmeLSP1b --------------------------------------
DmeLSP1a --------------------------------------
DmeLSP1g --------------------------------------
Aga31208 --------------------------------------
BmoSP2 --------------------------------------
HmeHC1 --------------------------------------
PamHC1 --------------------------------------
CseHC1 --------------------------------------
CmoHC1 --------------------------------------
SamEHP --------------------------------------
CacHC1 --------------------------------------
PmaHC1 --------------------------------------
ScuHC1 --------------------------------------
CmaHC1 --------------------------------------
NviHEX109 --------------------------------------
NviHEX102 --------------------------------------
AmeHEX110 --------------------------------------
AmeHEX70c --------------------------------------
CfeHEX2 KDTINESWLKENRMKDTMDENTMNL-------------
NviHEX94 MMQDIKKPSMMDMPEHSSMTMKTDYTMERMRDHNMMHV
NviHEX83 --------------------------------------
AmeHEX70a --------------------------------------
NviHEX81 --------------------------------------
AmeHEX70b --------------------------------------
NviHEX79 --------------------------------------
NviHEX75 --------------------------------------
TcaHEX1A -------------QGQYYKGQNFDSQYYHY--------
TcaHEX1B YPEQYYAQGSNYAQEQYYPSRSYERQDYYHY-------
TcaHEX2 --------------------------------------
TmoHEX2 --------------------------------------
TcaHEX3 --------------------------------------
TcaHEX4 --------------------------------------
TcaHEX5 --------------------------------------
RflHEXII --------------------------------------
RflHEXI RFSGMCCRILW---------------------------
LmiJHBS --------------------------------------
HzeHEX M-------------------------------------
TniJHSP2 V-------------------------------------
HceHEX1 M-------------------------------------
HceHEX2 --------------------------------------
HviHEX --------------------------------------
BmoSP1 --------------------------------------
CfuDAP2 --------------------------------------
Dme7320 --------------------------------------
DmeFBP1 --------------------------------------
Dme8100 --------------------------------------
